# Integrated Community Care Delivered by Public Health-Care and Social-Care Systems: Results from a Realist Synthesis

**DOI:** 10.5334/ijic.7042

**Published:** 2024-02-16

**Authors:** Jean-François Allaire, Paul Morin, Chantal Doré, Shelley-Rose Hyppolite, Marie Suzanne Badji, Hervé Tchala Vignon Zomahou

**Affiliations:** 1 Institut universitaire de première ligne en santé et services sociaux (IUPLSSS) du Centre intégré universitaire de santé et de services sociaux de l’Estrie –Centre hospitalier universitaire de Sherbrooke (CIUSSSE-CHUS), Hopital et centre d’hébergement D’Youville, Sherbrooke, QC, Canada; 2 Université de Sherbrooke, Canada; 3 School of Nursing and Researcher, Université de Sherbrooke, Canada; 4 Centre intégré universitaire de santé et de services sociaux de la Capitale Nationale, Canada; 5 Faculté de médecine, Université Laval, Canada; 6 Department of Social and Preventive Medicine, Faculty of Medicine, Université Laval, Quebec City, Quebec, Canada; 7 Institut national d’excellence en santé et services sociaux, Quebec, Québec, Canada

**Keywords:** integrated community care, health care, social care, public services, proximity, local area, Interventions en santé et services sociaux intégrées en proximité des communautés, soins de santé, soins sociaux, services publics, proximité, territoire

## Abstract

**Introduction:**

Integrated community care (ICC) is defined as an interweaving of health-care and social-care interventions deployed in spatial and relational proximity using an interdisciplinary and cross-sectoral approach. Consideration of territory scale and time scale are at the center of ICC practices. Its deployment in public health and social care networks (HSCN) can be complex due to their broad mandate, the complexity of their management, and accountability. Therefore, we aimed to describe ICC delivered by public HSCN to determine how, why, for whom, and in what circumstances ICC works and produces outcomes.

**Methods:**

A realist synthesis was conducted consisting of five steps consistent with realist synthesis standards (RAMESES projects) to produce configurations of Context – Mechanism – Outcomes (CMOc) and development of a middle-range explanatory theory of why and how the identified outcomes may have occurred.

**Results:**

In total, 26 studies were selected and used, as evidence, to support—either partially or fully—the production of CMOc based on the initial program theory. Nine unique CMO configurations were identified based on the data analyses and team discussion. ICC middle-range theory is informed by the CMO configurations identified.

**Discussion:**

This realist synthesis allowed us to identify the central mechanisms of ICC delivered by public HSCN and to produce a middle range theory. ICC is based on a specific philosophy and deployed by a professional agency oriented toward a community agency within a local system of interdisciplinary and cross-sectoral action.

**Conclusion:**

Our middle-range theory will provide a solid analytical framework as a foundation for ICC implementation and future research.

## Abstract (fr)

**Introduction:**

Les interventions en santé et services sociaux intégrées en proximité des communautés (IIPC) sont définies comme une imbrication d’interventions de soins de santé et de services sociaux à l’échelle du territoire et considérant la temporalité, déployées dans une proximité spatiale et relationnelle au moyen d’une approche interdisciplinaire et intersectorielle. Son déploiement dans les réseaux publics de santé et de soins sociaux (RSSS) peut s’avérer complexe en raison de l’étendue de leur mandat, de la complexité de leur gestion et de leur responsabilité. C’est pourquoi nous avons cherché à décrire les IIPC dispensées par les RSSS publics afin de déterminer comment, pourquoi, pour qui et dans quelles circonstances les IIPC fonctionnent et produisent des résultats.

**Méthodes utilisées:**

Une synthèse réaliste a été réalisée en cinq étapes conformes aux normes de synthèse réaliste (projet RAMESES) afin de produire des configurations Contexte – Mécanisme – Effets (CMOc) et de développer une théorie explicative de moyenne portée sur le pourquoi et le comment des résultats identifiés.

**Résultats:**

Au total, 26 études ont été sélectionnées et utilisées comme preuves pour étayer – partiellement ou totalement – la production de CMOc sur la base de la théorie initiale de programme. Neuf configurations uniques CMO ont été identifiées sur la base des analyses de données et des discussions de l’équipe. La théorie de moyenne portée des IIPC s’appuie sur les configurations CMO identifiées.

**Discussion:**

Cette synthèse réaliste nous a permis d’identifier les mécanismes centraux des IIPC déployées par les RSSS publics et de produire une théorie de moyenne portée. Les IIPC sont fondées sur une philosophie et le développement d’une capacité d’agir professionnelle mise en action vers le renforcement de la capacité d’agir de la communauté au sein d’un système local d’action interdisciplinaire et intersectoriel.

**Conclusion:**

L’utilisation de notre théorie de moyenne portée pour la mise en œuvre d’IIPC fournira un cadre analytique solide comme base pour des implantations ou des recherches futures.

## Introduction

Integrated community care (ICC) is defined as “an interweaving of localized and temporalized health and social care interventions provided in proximity (spatial and relational) in an interdisciplinary and cross-sectoral manner. ICC aims to improve physical and mental health, well-being and empowerment, as well as to facilitate access to and use of care, particularly among disadvantaged populations or those not served by the health and social care system” [1, p. 5]. An ICC intervention can be an individual, group, or collective intervention that uses the strengths and interests of individuals, communities, and partners as levers to meet the needs of the population [1, Appendix 1]. Moreover, it combines several intervention strategies: citizen participation, outreach and support services, joint intervention, networking actions, mobilization, and the empowerment of the population on social-capital components. The intervention can be carried out by various types of professionals such as social workers, physicians, and nurses [Appendix 1].

ICC can be distinguished from conventional care by its delivery based on four characteristics:

- The approach used: health care or social care centered on or integrating health and social care.
- The environments that deploy them: the public health-care and social-care network; the community, municipal, and private sectors; or through collaboration between these sectors.
- The target population: disadvantaged communities, addressing all or major population groups (e.g., seniors, families, residents of social-housing complexes, immigrants) of a local area in which vulnerable or marginalized (material or social disadvantages) populations live, while specifically taking into account populations on the fringes of the health-care and social-care system.
- The targeted outcomes: improve health and well-being of individuals and communities; health equity; social capital within the community; social networks; social cohesion; participation in co-production, improvements in the accessibility, availability, and continuity to health care and social care; and actions on the social determinants of health.

Deploying ICC in public health-care and social-care networks (HSCN) can be a complex task due to the type of outcomes targeted but also the type of management and accountability that ICC requires [2,3]. Indeed, in terms of management, HSCN tend to be centralized [4] with accountability based on quantitative indicators (number of interventions, number of users, duration and frequency of interventions, etc.) [5], while ICC gravitates toward what Levesque et al. [6] call decentralized, collaborative, citizen governance requiring accountability combining quantitative and qualitative indicators (improvement of well-being, individual and collective empowerment, social capital, etc.) focused on “meaning.” Moreover, beyond its deployment complexity, ICC is considered complex because the outcomes expected following its deployment are neither constant, immediate, nor systematically observable [1]. Indeed, outcome production can vary according to the deployment context (types of community setting, types of stakeholders, and types of approaches); the characteristics of individuals, groups, and populations; and the characteristics of the local area and temporality.

Thus, in order to better understand ICC and to encourage its practice in public HSCN, a realist synthesis project was conducted to document its functioning and to analyze the processes that produce the targeted outcomes. The published protocol [7] presents the details of the realist synthesis project. This paper presents the results of the realist synthesis.

## Methods

The realist synthesis was carried out in several stages, as mentioned in the published research protocol [7]. The research question was developed and progressively evolved during the selection and evaluation of publications. The selection process allowed reviewers to make sense of the research question; develop, refine, and test theories; and support conclusions about the mechanisms that generate outcomes in certain contexts. The final research question was: How, why, for whom, and in which contexts does the ICC deployed by public HSCN work and produce outcomes?

Realist synthesis contrasts with the more common systematic review. Pawson et al. [8,9,10,11] developed an approach in response to the question “…what works for whom in what circumstances, and in what respects?” [10]. Rather than limiting the scope of the synthesis to those with specific types of research designs with common indicators, the realist approach is based on a belief that any research method can contribute understanding and knowledge. Thus, a realist synthesis relies on the expertise of the reviewers to compile a summary of outcomes and build an explanatory theory (middle-range theory) of why and how these outcomes might have occurred. What is lost in replicability is gained in a comprehensive and rich explanation of a phenomenon. This approach used in the current synthesis uses the term “mechanism”, that should not be confused with the general use of the term in program evaluation, which refers to the activity or mode of operation of the program being evaluated [12]. In a realist approach, mechanisms are the way in which the actors (stakeholders as well as beneficiaries) use the resources made available by the intervention to achieve the change targeted by the intervention [13]. These mechanisms are sensitive to variations in context and are generally not directly observable [14]. Different steps consistent with realist synthesis standards [15] were followed: (A) identifying the initial program theory, (B) searching for evidence, (C) selecting documents, (D) extracting and organizing data, and (E) synthesizing evidence and drawing conclusions.

### Initial program-theory development

A rough theory on the functioning of ICC was developed in order to better understand implementation processes, that is, the mechanisms that can be activated by stakeholders and the process of producing outcomes. This rough theory was designed in the form of a realist program theory [16,17,18]. The theory was based on a document developed by our team on ICC practices [19] and went through an iterative process of refinement as the research progressed. The team engaged in the process was led by the main authors. Research-team members contributed to the discussion. We had a meeting on rough theory with the project’s advisory committee composed of researchers and health-care and social-care workers to hear their comments.

### Searching for evidence

The search process was designed with keywords (controlled or free vocabulary) from the PICOSS [Appendix 1] (Population/Intervention/Comparison/Outcome/Study design/Setting) [20] approach of Cochrane systematic reviews based on the research question. This process is based on a systematic review method and explained in the Appendix 1 and in the full search strategy [Appendix 2]. The literature search was conducted on Ovid Medline, Elsevier Embase, EBSCOhost CINAHL, Ovid PsycINFO, Proquest – Sociological Abstracts, Web of Science Core collection, ÉRUDIT (queries in English and French), and CAIRN (queries in English and French) for scientific studies. The research team was able to call upon its international network of contacts (mainly based in Canada, Italy, England and Scotland) to search for grey literature. However, in this category of publication, owing to limited resources, only publications written in French, English or Italian were included. The choice of Italian is explained by Italy’s advances in the field of ICC but also by the fact that one of the researchers understands this language. Various sources of grey literature known to the research team were also consulted.

### Selecting documents

Different steps were followed to select relevant papers based on inclusion and exclusion criteria (Table 1) whose choices are explained in the protocol of the review [7, see methods section]. Two reviewers—members of the research team—independently participated in selecting studies. Step 1: Conduct a pilot run on a sample of papers identified in the databases to reach a consensus on the selection criteria and to clarify them. Step 2: Independently rate the papers and abstracts as included, excluded, or unclear based on their titles and abstracts. The items went into the next step of assessment. Step 3: Perform the final selection by reading full texts using the same criteria (i.e., texts rated included or unclear in step 2). At the end of each step, any disagreements were resolved by discussion to reach a consensus. If a consensus could not be reached, two other members of the research team were called in to make a final decision. The flow diagram of the search results presented according to PRISMA 2020 standards is available in Appendix 1.

**Table 1:** Inclusion and exclusion criteria.


DIMENSION	INCLUSION CRITERIA	EXCLUSION CRITERIA

**Type of studies**	Empirical studies published in any language for scientific papers, and in English, French or also Italian for grey literature	Studies with a narrow challenge found only in a specific local area and abstracts of conferences without a follow-up full text were excluded

**Period of publications**	Initially, published between January 2003 and March 2019. Extended to February 2021	—

**Target population and area**	Linked with target populations: all or major groups in the population (e.g., seniors, families, residents of social-housing complexes) and vulnerable or marginalized (material or social disadvantages) populations within specific territories. Large population groups were also considered (e.g., the elderly, vulnerable families, the disadvantaged, marginalized populations with complex problems, etc.)	Papers related to specific needs of a limited population were excluded, as these studies often focused on specific issues that might not have represented an approach that could be generalized across a population (e.g., retired soldiers living with posttraumatic stress disorder in a specific neighborhood).

**Type of intervention**	Integrated community health and social care with the potential for universal, population-level outreach (available and accessible to all in a specific local geographic area).	—

**Intervention leadership**	Deployed by public health-care and social-care networks, with or without the collaboration of the community, other institutions, or private partners	Interventions deployed by the community or private sector


### Extracting and organizing data

Our approach to data extraction was based on various guides and past related systematic reviews [8,21,22,23]. We then iteratively refined our procedures according to the focus of our review. The reviewers initially extracted data [coding grid in Appendix 3] from a sample of four studies and discussed the data extracted with other members of the review team to improve the accuracy of data extraction. Data extraction was carried out in two sections: PICOSS characteristics [details in Appendix 1] and Context-Mechanism-Outcomes (CMO) configurations. We identified context elements, outcomes, and mechanisms in the text. Sometimes, the mechanisms had already been identified but, usually, we had to bring the mechanism to light, linking it to outcomes [15].

### Synthesizing evidence and drawing conclusions

The research team first summarized the results from the systematic review [Appendix 1]. Then, for the analysis of the results from the realist synthesis, the research team members used an iterative process to develop the explanatory mechanisms and CMO configurations. A mechanism that is real but hidden lies at the heart of the intervention process and is linked with outcomes and contextual elements [15]. The extracted data were used to test and refine our rough theories for each inferred mechanism, using the CMO structure. In each of the included publications, “nuggets of evidence” [15] were searched for in the extracted data to identify whether the inferred mechanism was in play and to understand its relationship with the associated context and outcomes using an actor-based approach (interveners, managers, governance). Nuggets of evidence were used to improve our rough theories and identify a new CMO configuration. Each CMO configuration consist of a context that can be favorable or not, a mechanism that can be activated by the context or not and outcomes that are produced when the mechanism is activated. The various CMO configurations were linked together to create a middle-range theory and to provide a perspective on ICC.

## Results

### Search results

Out of the 14,748 studies published between January 2003 and February 2021 (duplicate records = 5686; ineligible records = 9003), 52 papers were preselected for complete reading based on their titles and abstracts. After the papers had been completely read, 18 scientific papers were selected for inclusion in the synthesis [24,25,26,27,28,29,30,31,32,33,34,35,36,37,38,39,40,41] [Appendix 4].

Initially, 18 grey literature texts published between January 2003 and February 2021 were identified through the research team’s international network of contacts (mainly in Canada, England, Italy, and Scotland). Applying the inclusion criteria resulted in eight grey-literature documents being retained [42,43,44,45,46,47,48,49]. Four of these texts were treated concurrently with other texts [35,37,41,49]. All the details on the search results can be found in Appendix 1.

Regarding the realist synthesis, different mechanisms and CMO configurations emerged from the analysis. Feedback loops between team members helped to improve the CMO configuration. Nine mechanisms were identified and grouped according to three main themes: philosophy guiding ICC, professional agency adapted to ICC, and mechanisms related to the local system of interdisciplinary and cross-sectoral action.

### Key contextual elements

The contexts related to the conditions of ICC deployment and practice presented in the systematic review [Appendix 1] highlight some general findings found in the CMO configurations presented herein. For instance, the principal target populations of ICC practices were mainly socially vulnerable, remote, or unreached by services; economically impoverished; and with chronic health or mental-health problems. The main issues identified in relation to accessibility, availability, continuity, and quality are unanswered health-care and social-care needs, access problems, and few service points in the local area. These are key points linked to access to and availability of services in the local area. Quality and continuity issues were linked mainly to fragmented health care and social care delivered, lack of community-based interventions, access to discontinuous health-care and social-care pathways, limits of single-discipline practices, access to episodic and low-quality care, and challenges to patient engagement. These contextual elements are consistent with the ICC goals and targeted outcomes.

### Philosophy guiding ICC

The philosophy guiding ICC is considered as “a group of theories and ideas related to the understanding of a particular subject” [50] and a perspective adopted on intervention, management, and governance of ICC practices. Three CMO configurations are related to ICC philosophy.

#### CMO1: Willing to recognize the importance to understand the intervention area, the stakeholders involved, and area population

**Figure 1:**
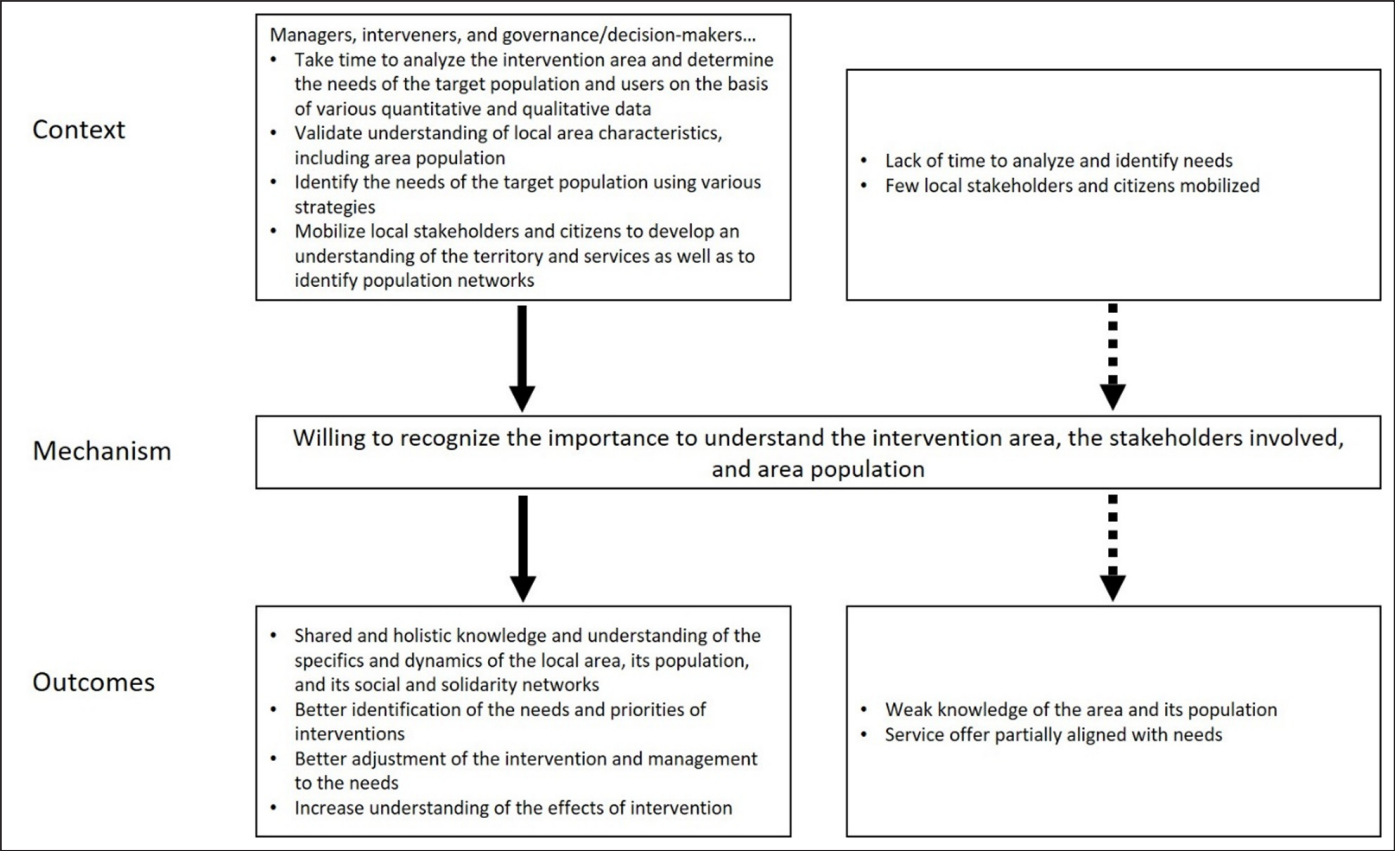
Context-Mechanism-Outcomes configuration 1.

When (C) managers and interveners take time (CMO2) to analyze the intervention area; determine the needs of the target population and users on the basis of various statistics (population data, community consultations, home services, hospital consultations, drugs…); validate their understanding of local-area characteristics, including its population (demographics, socio-sanitary, health, social and cultural factors); identify the needs of the target population using various strategies (surveys, meetings, citizen activities…); and mobilize local stakeholders and citizens to develop an understanding of the territory and services and to identify population networks (Figure 1).

*a willingness to recognize the importance to understand the territory of intervention, the stakeholders involved, and area population is present (M)*.

It is possible to observe a shared and holistic knowledge and understanding of the specifics and dynamics of the local area, its population, and its social and solidarity networks (O). This makes it possible to better identify the needs and priorities of interventions (places, populations, types of intervention…); to adjust the intervention and management to the needs and to better understand the effects of interventions, including on social capital and the social determinants of health.

#### CMO2: Recognize and understand the different perspectives on temporality, in interventions as well as in management and governance

Deploying an ICC team in a local area constitutes a change of practice (CMO4, CMO6, and CMO7) in a system that is sometimes segmented or hospital-centric. Understanding the dynamics of the local area (CMO1) and the principles and strategies to be implemented in ICC (CMO4) helps to deploy ICC in a context of limited time despite the complexity of the cases and the time needed to establish bonds. The temporality is different depending on the perspective of the various stakeholders or people in the local area and is the need to synchronize the actions of different actors and to synchronize the times on people’s needs [1].

*It is therefore necessary for interveners, managers, and governance to recognize and understand the different perspectives on temporality (M)*.

**Figure 2:**
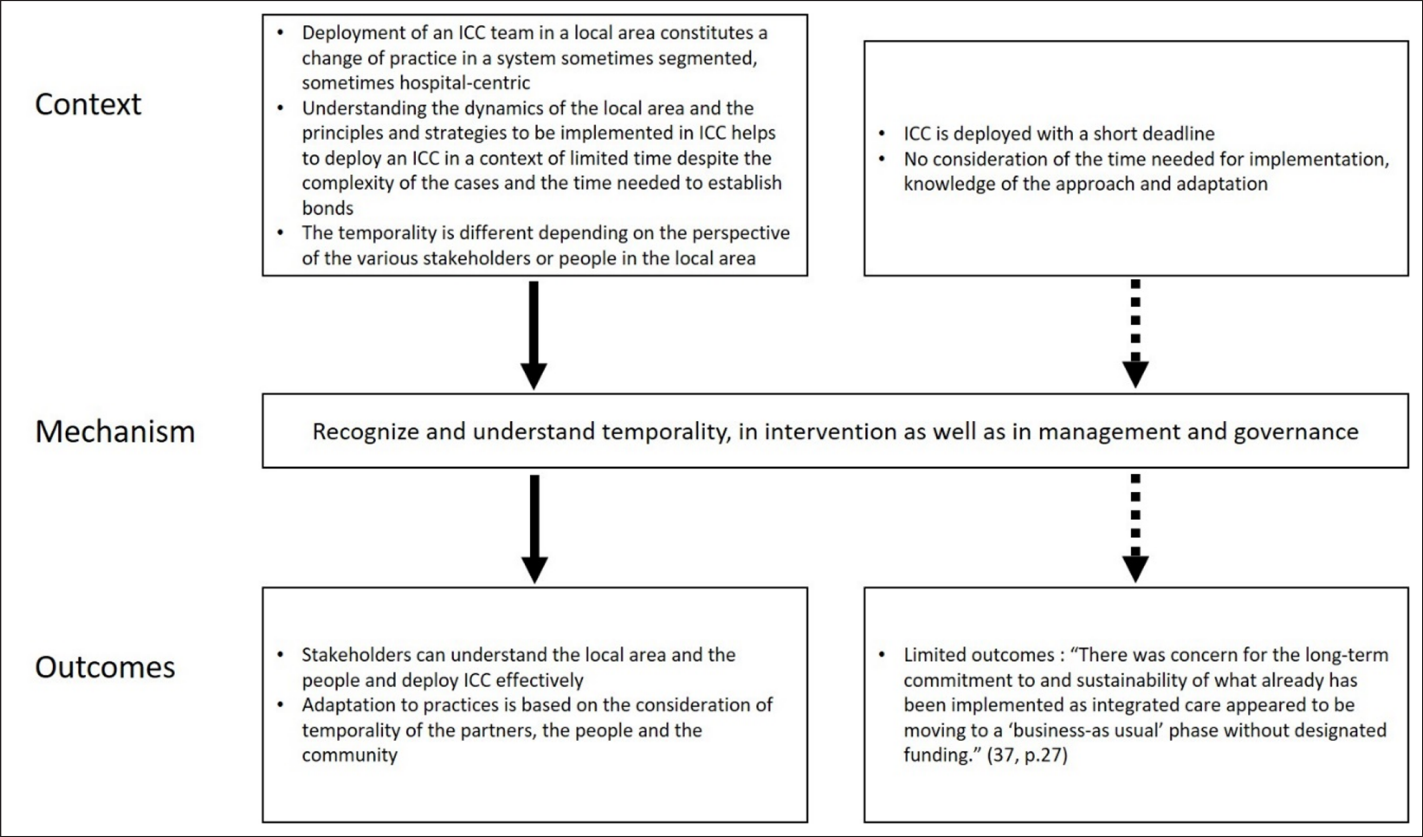
Context-Mechanism-Outcomes configuration 2.

When temporality is considered (O), stakeholders can understand the local area and the people in its complexities and deploy ICC effectively. Temporality of the partners, the people, and the community is also considered in adapting practices (Figure 2).

“Let things happen without forcing, keeping them from not happening and not insisting to make them happen” [46, p. 4].

#### CMO3: Actualize a collective and partnership learning culture

ICC implementation that is an individualized, group, and collective intervention adapted to the population’s realities, needs, or issues in a targeted community in an evolving context (change in population composition, new services, construction of social housing, emergence of security issues…) is linked to unstable public policies and funding and to the implementation of actions to test, monitor, and learn. In Trieste, “Reforms meet resistance and obstacles, either from actors involved in implementing projects, or from the recipients of the policies, that is, the inhabitants” [Translated from French, 47, p. 60].

When acting on the complex individual and collective realities of people requires reflection, co-development and co-intervention practices are sometimes put in place to share, to take into account the issues of time (CMO2) and place, confidentiality, liaison, data sharing, and the challenges of coordinating intervention between partners from different organizations.

“I really do believe…it’s bringing all the agencies together to brainstorm how we’re going to and listening to people’s expertise around the table” [Stakeholder, 26, p. 11]

*This context leads to actualizing a collective and partnership learning culture (M) based on a reflective, iterative, and respectful posture of the opinions and realities of the various stakeholders*.

**Figure 3:**
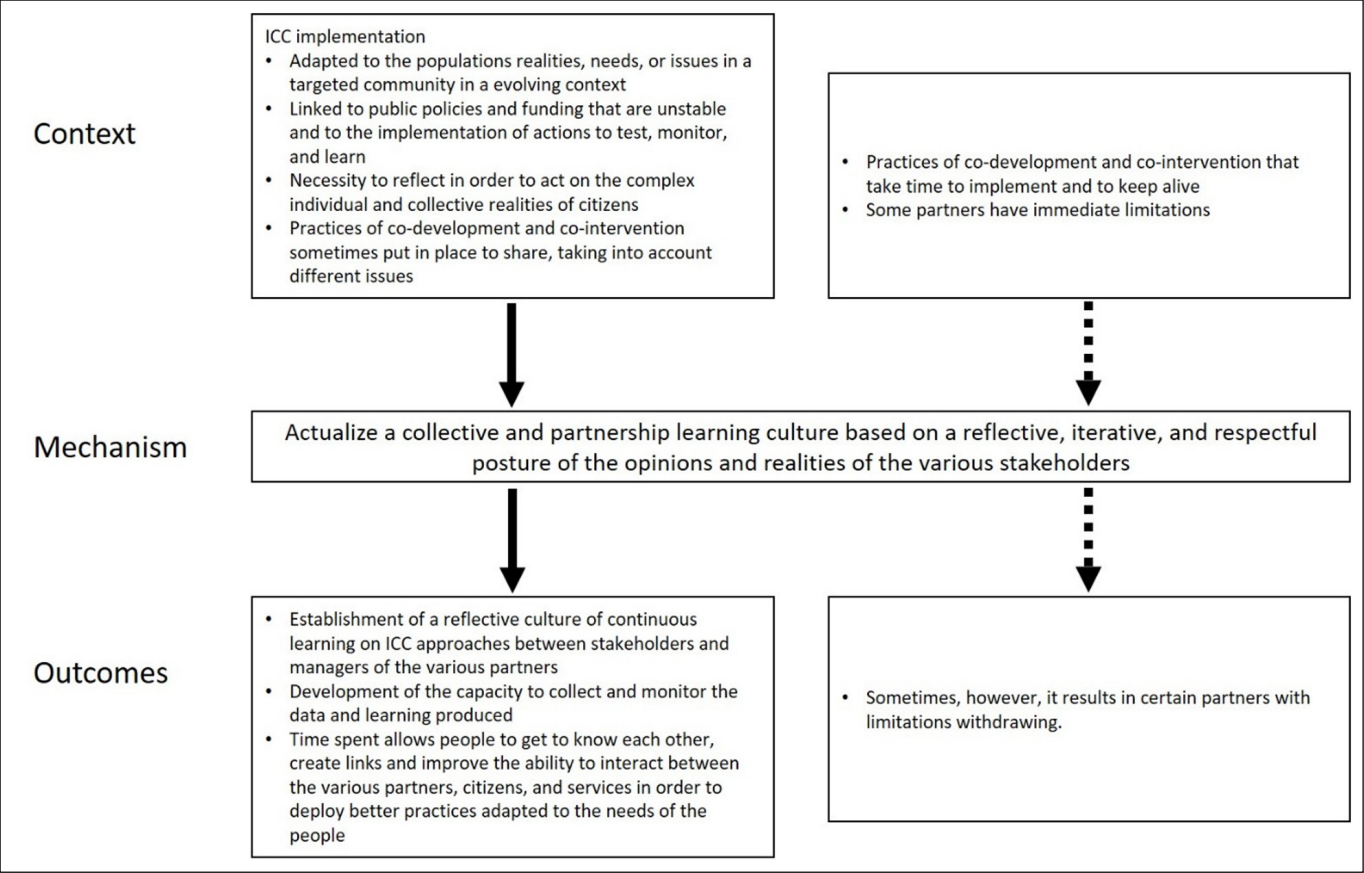
Context-Mechanism-Outcomes configuration 3.

The outcomes (O) are the establishment of actions enabling continuous learning on ICC approaches between stakeholders and managers of the various partners, especially public HSCN, and the development of the capacity to collect and monitor the data and learning produced. This leads to experimentation and innovation. The learning culture allows people to get to know each other, create links, and improves the ability to interact between the various partners, citizens, and services in order to deploy better practices adapted to the needs of the people. Sometimes, however, it results in certain partners with limitations (funding, mandate, human resources) withdrawing (Figure 3).

### From a professional agency to a community agency adapted to ICC

Practitioners exert influence, make choices, and take stances in ways that affect their work or their professional identities [51]. Community agency is considered the capacity for collective action in a specific community/local area [52] and is closely related to empowerment [53]. Professional agency oriented toward community agency is at the center of our middle-range theory of ICC.

#### CMO4: Recognize and understand the needs, strategies, and specificities of ICC interventions

When ICC takes a flexible, holistic, preventive, nonjudgmental approach, with low barriers to access to spatial and relational proximity, is adapted to local area context and community needs, is deployed with intervention and accountability tools adapted to the local area, and acts on various health determinants, interdisciplinarity, and cross-sectorality (C), …

*stakeholders, managers, and decision-makers recognize and understand the strategies and specificities of ICC (M)*.

**Figure 4:**
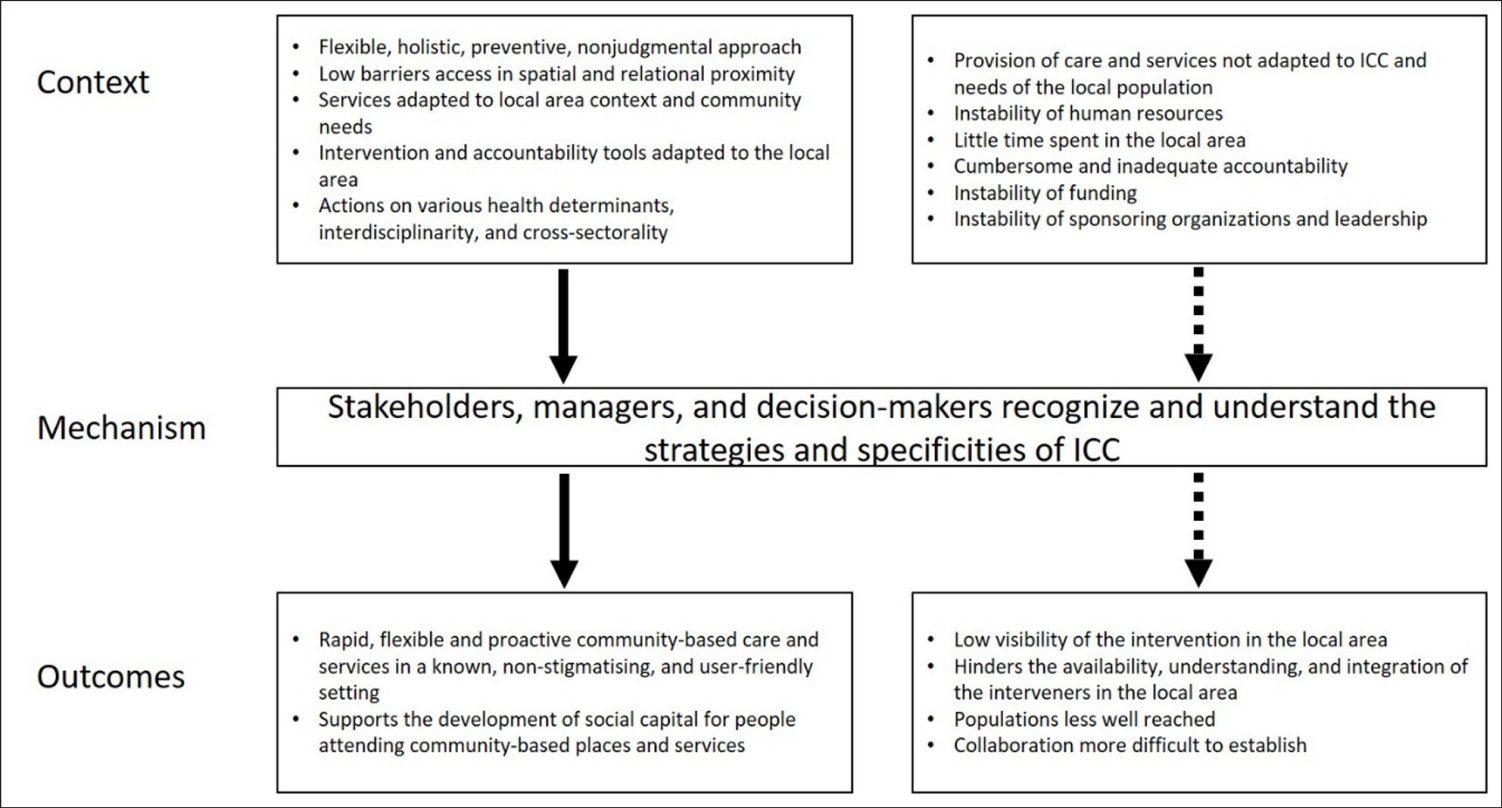
Context-Mechanism-Outcomes configuration 4.

This produces (O) rapid, flexible and proactive community-based care and services in a known, non-stigmatizing, and user-friendly setting (gathering places integrating services and promoting their continuity, bringing the population and institutions closer together), particularly for people not reached by the traditional public system, which supports the development of social capital for people who go to community-based places and services (Figure 4).

“In the first two years of operation, the clinic was able to decrease the number of monthly visits by high frequency patients (those who came to be seen more than 50 times a year) by almost 20%.” “Emergency room visits to the nearby hospital were decreased by 20%” [28, p. 6].

#### CMO5: Willing to promote the ICC practice and to take into consideration the local area in governance and management

When services are deployed close to the population, in a context of access to care and offering information that is often complex to understand for people, despite the varying degrees of autonomy offered to interveners and public policies that unequally recognize the importance of proximity to the population and the local area…

*the will to promote ICC practice and to take into consideration the local area by managers and governance actors is central (M)*.

**Figure 5:**
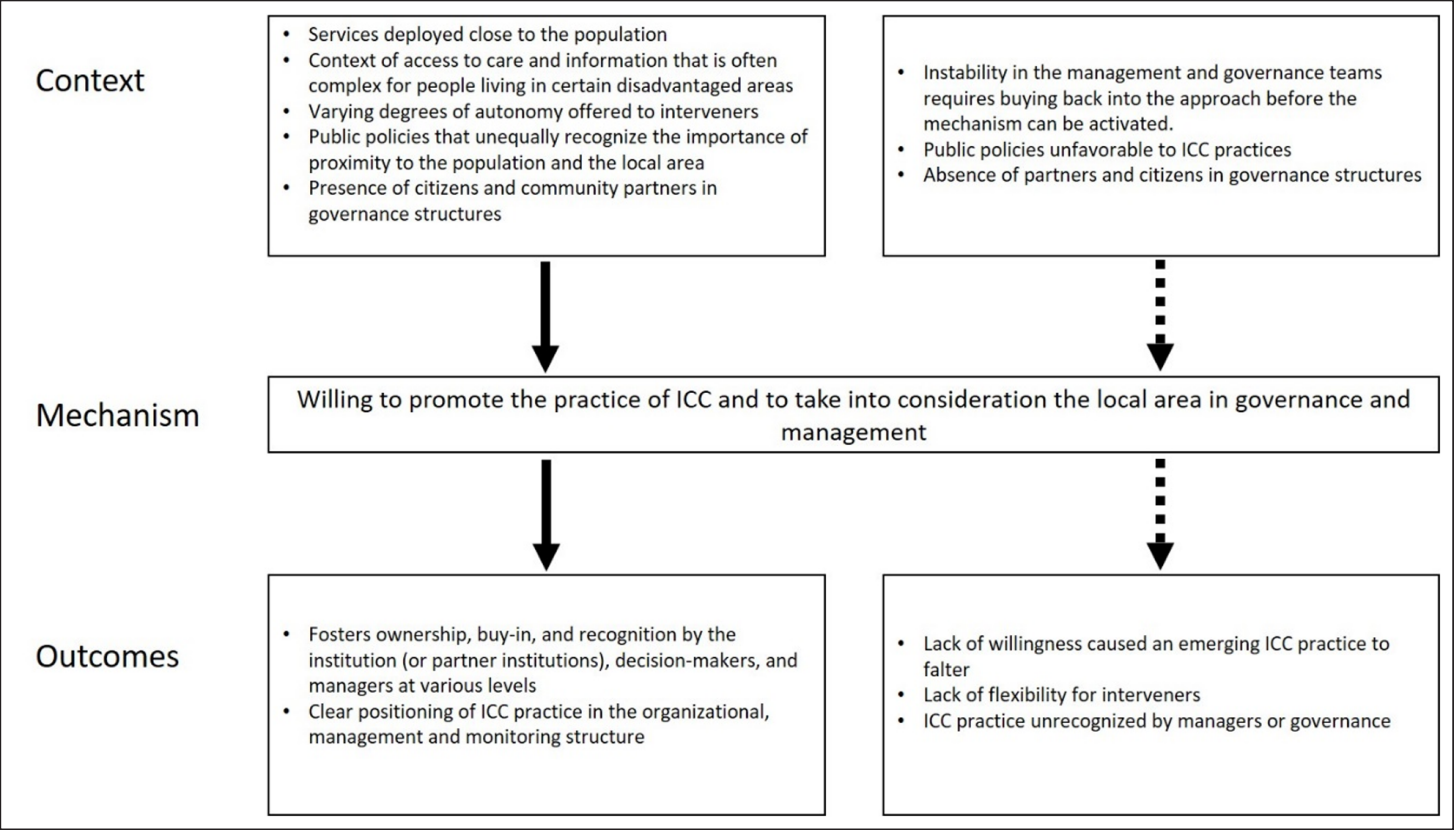
Context-Mechanism-Outcomes configuration 5.

This fosters ownership, buy-in, and recognition by public HSCN (or partner institutions), decision-makers and managers at various levels, as well as the clear positioning of ICC practice in the organizational, management, and monitoring structure (O). In some cases, this willingness was central for managers and decision-makers to anchor ICC practice in the service structure. In other cases, this lack of willingness caused the emerging ICC practice to falter. The presence of citizens and community partners in governance structures helps to activate the mechanism. A context of instability in the management and governance teams impacted the mechanism’s activation (Figure 5).

#### CMO6: Willing to embody an approach of people-centered care

When the development of community-based care and services that reach out to the population (CMO7) is adapted to the citizens/users and their relatives needs and capacities; respects their values, culture, expectations, and preferences at their own pace with a nonjudgmental approach, recognizing the knowledge and perspectives of each person and co-constructing services with cultural sensitivity with the population and local partners. Although it can be complex with relatives, the ICC team must have a…

*willingness to embody an approach of people-centered care with the people, families, and community (M)*.

**Figure 6:**
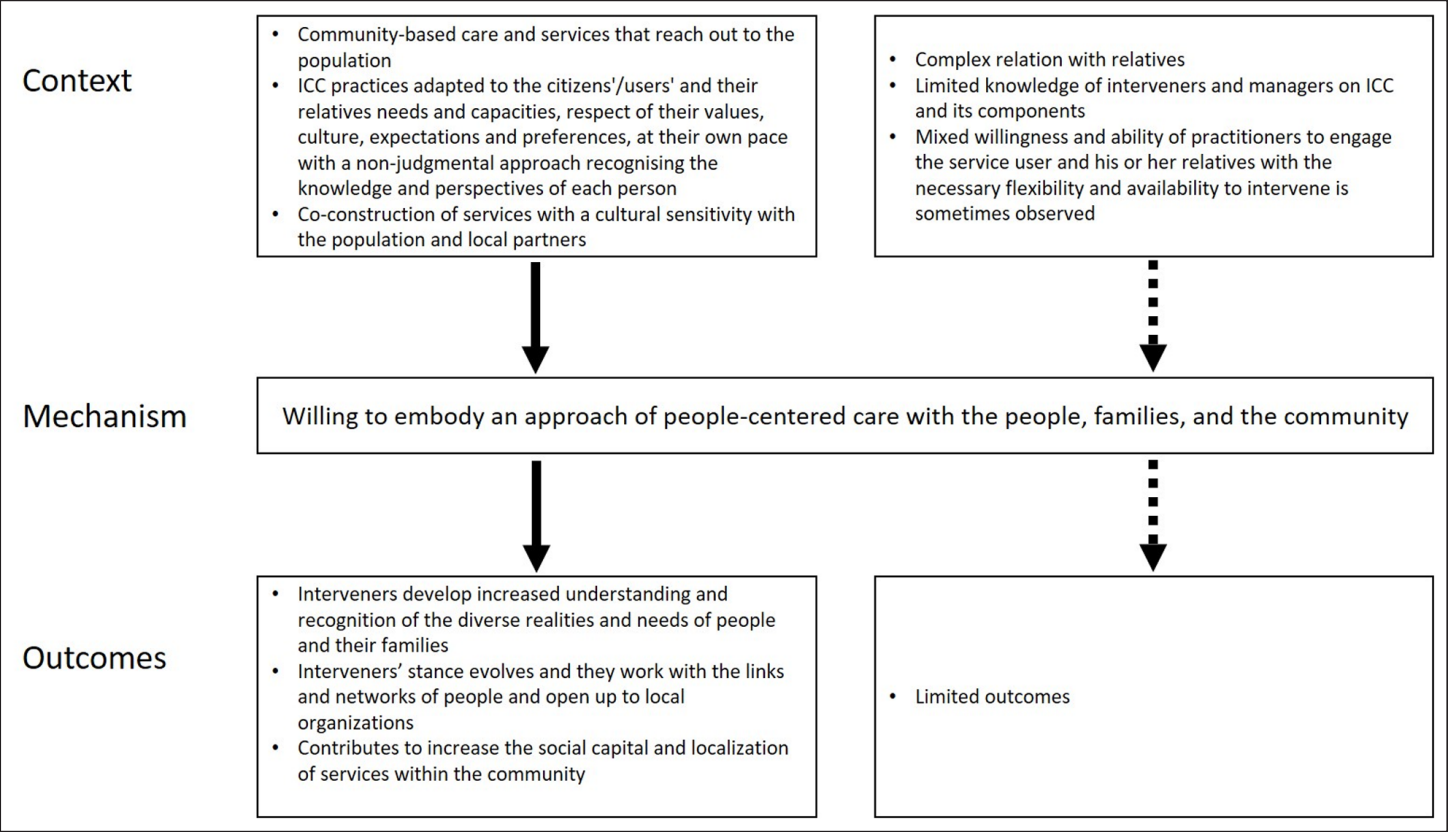
Context-Mechanism-Outcomes configuration 6.

Interveners (O) develop an increased understanding and recognition of the diverse realities and needs of people and their families. Their stance evolves and they work with the links and networks of people and open up to local organizations, which contributes to increase the social capital and localization of services within the community (Figure 6).

In Trieste, “microareas experimentation enlarge this philosophy […], face-to-face relationships with persons in the context they live make operators feel the need to plan specific interventions for each one: they must regard where a person lives, the way he/she lives, his/her social relationships, his/her income etc.” [46, p. 4].

“Do you trust them? Participant: It’s good and the point is you don’t have to worry too much about how you’re seeming to them, you can just be yourself, and they will try to help in the best way they can” [38, p. 10].

#### CMO7: Base its action on spatial and relational proximity, outreach, and an individual and collective empowerment approach

When ICC is deployed, it means that interveners must (a) deploy welcoming and accompanying actions and reach out to partners and the population where they can be found in the community (festivities, activities, shops…); (b) develop links and relationships (development of bonds with the population, self-help networks, empowerment…); and (c) focus on approaches centered on strengths, potentials, involvement, and resources of individuals, groups, and the community…

*ICC teams base their actions on an approach (posture) of proximity (spatial and relational) outreach and individual and collective empowerment (M)*.

“[…] she also came to me. That was the difference. Often, we isolate ourselves. We don’t want to talk about what we are going through, or we don’t want to disturb others. Even when things were not going well, she made sure that I was never left behind” [Translation from French, 44, p. 18].

**Figure 7:**
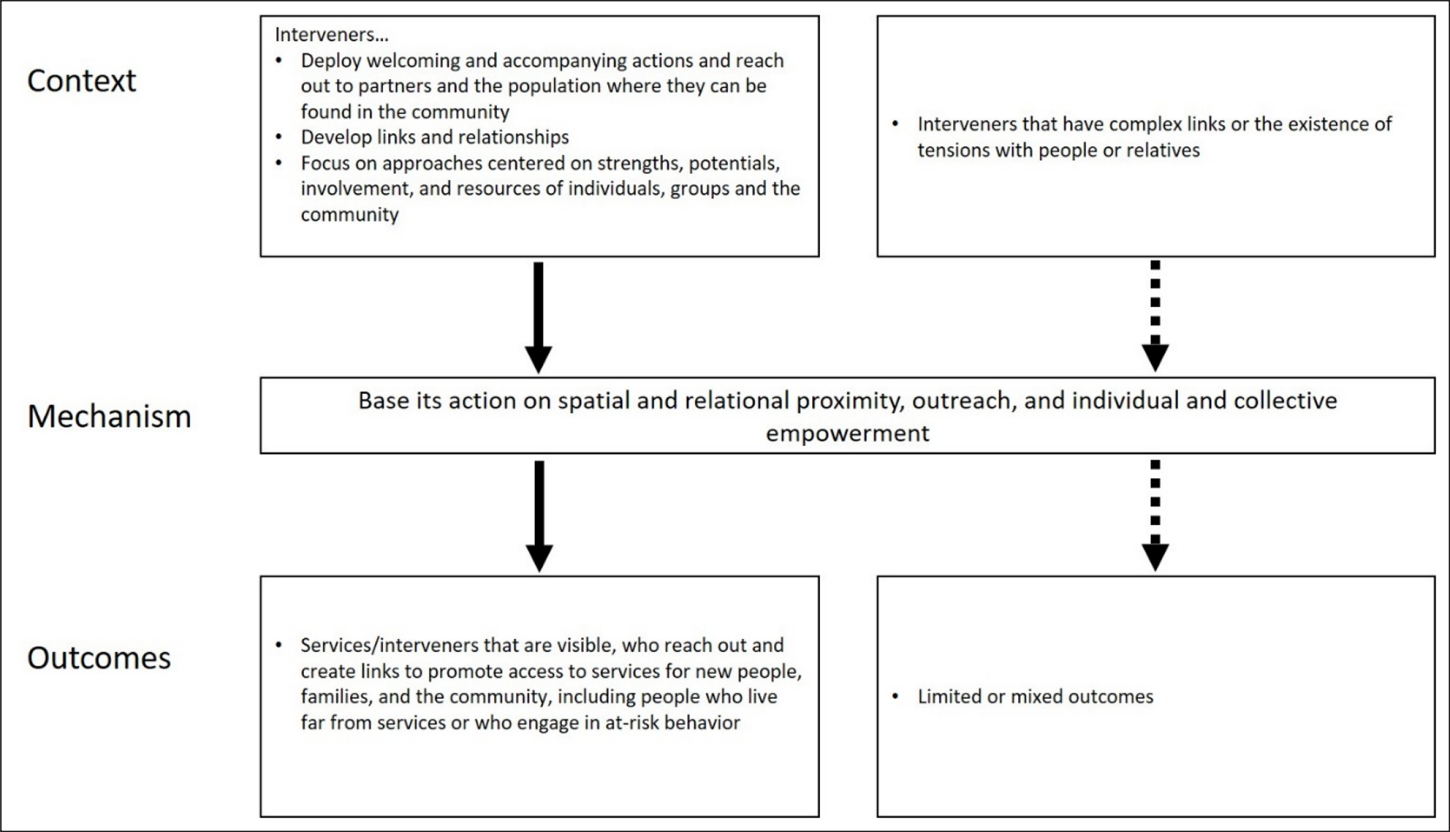
Context-Mechanism-Outcomes configuration 7.

This makes the services/stakeholders visible (O). It also reaches out and creates links, promoting access to services for new people, families, and the community, including people who live far from services or who engage in at-risk behavior (removal of physical or psychological barriers to access) (Figure 7).

### A local system of interdisciplinary and cross-sectoral action

A local system of action is a conjunction of actors on a local territory. “The local system of action is based on the links of proximity and trust between actors, a network linking the different actors…” [54, p. 73]. The system of action in ICC is deployed by stakeholders from various disciplines and partners from various sectors.

#### CMO8: Take a proactive approach to interdisciplinary teamwork in synergy with health and social care in proximity to the population

When (C) the provision of care is linked with outreach practices, the culture of a given community, and the needs of the local population, ICC implements interdisciplinary practices based on the collaboration of a set of interveners and managers (co-management of public services), based on sound knowledge of the local area (CMO1), relying on collective learning strategies (CMO3), and combining various types of expertise beyond exclusively health and social care in a long-term perspective (CMO2).

*Thus, taking a proactive approach to interdisciplinary teamwork in synergy with health and social care in proximity to the population is central (M)*.

**Figure 8:**
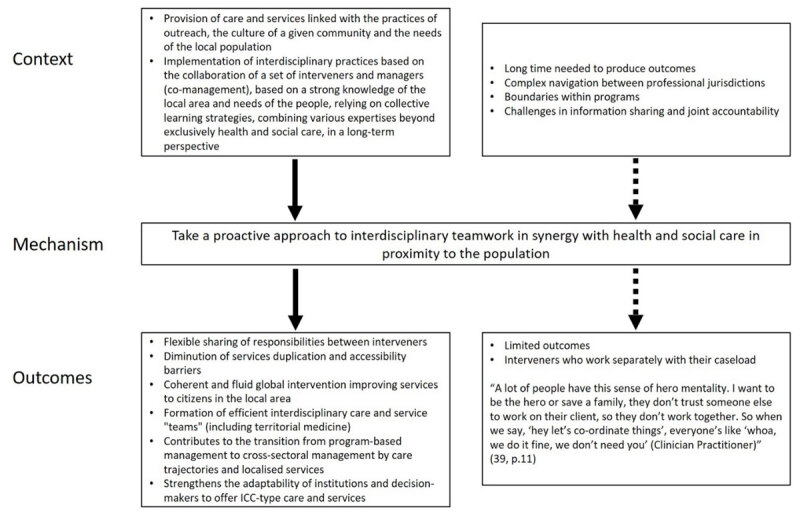
Context-Mechanism-Outcomes configuration 8.

The outcomes (O) observed are a flexible sharing of responsibilities between different kinds of interveners and reduction of service duplication and accessibility barriers, a coherent, fluid and global intervention improving services to people in the local area. This takes the shape of interdisciplinary public care and service “teams” (including territorial medicine) who have complementary skills and are motivated to collaborate, refer, and respond more effectively to needs (e.g., good service and prevention in the local area instead of recourse to the medical emergency room). The activation of the mechanism facilitates the transition from program-based management to cross-sectoral management through care trajectories and localized services. It strengthens the adaptability of institutions and decision-makers to offer ICC-type care and services (Figure 8).

#### CMO9: Willing to act in synergy on common targets with formal and informal cross-sectoral partners

When organizations from various public, community and private sectors (public HSCN, community organizations, social housing, public safety, local elected officials, social-economy organizations, or private companies) act on common issues related to revitalizing a local area—thus having a direct or indirect effect on the quality of life, health and safety—challenges appear in connecting the various services offered in the area. Such challenges include a culture of collaboration and trust in a context of different norms; organizational cultures; political or funding issues; instability of leaders, partners or decision-makers; different perceptions of ICC; specialized languages; and different time frames between partners (CMO2). Actions are sometimes co-constructed with partners, such as the creation of spaces for collective reflection and learning (CMO3), co-location of services (CMO4), and link-oriented management (agreements, communication mechanisms, co-construction of services).

*We observe a willingness to act in synergy on common targets with formal and informal cross-sectoral partners centered on ICC (M)*.

**Figure 9:**
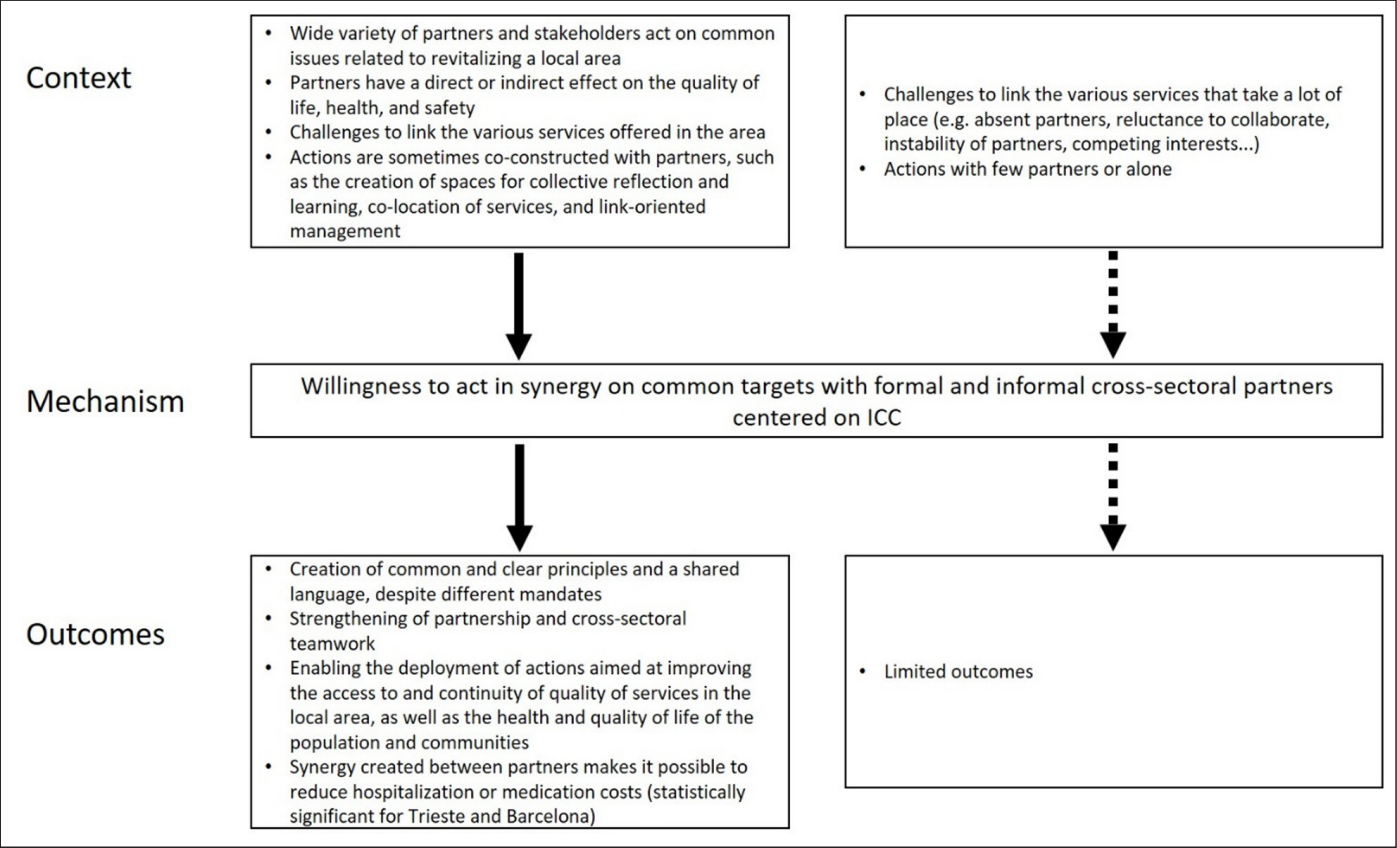
Context-Mechanism-Outcomes configuration 9.

The outcomes (O) identified are the creation of common and clear principles and a shared language, despite different mandates, and the strengthening of partnership and cross-sectoral teamwork (synergy, enduring links between stakeholders or managers, trust, exchange of information, sharing of expertise, agreements). This makes it possible to deploy actions aimed at improving access to and continuity of quality of services in the local area, as well as the health and quality of life of the population and communities. In addition, the synergy created between partners makes it possible to reduce hospitalization and medication costs (statistically significant for Trieste and Barcelona) (Figure 9).

## Discussion

**Figure 10:**
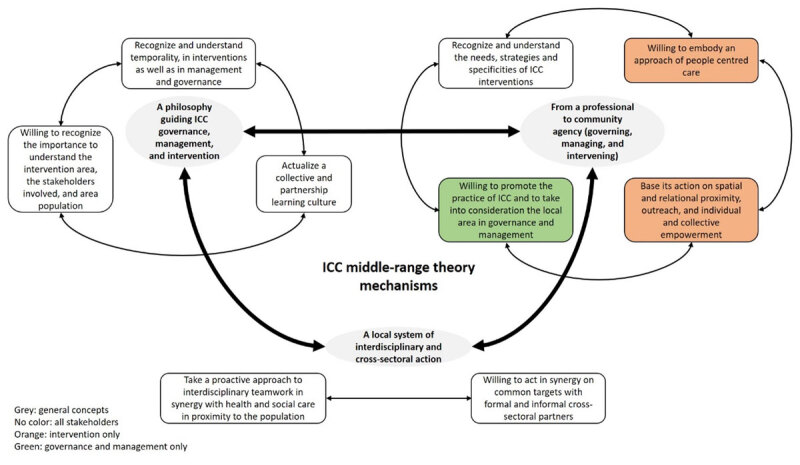
ICC Middle-range theory.

This realist synthesis allowed us to identify the central mechanisms of ICC delivered by public HSCN and to produce a middle-range theory presenting the connections between the mechanisms (Figure 10). The middle-range theory of ICC is based on a philosophy, a specific professional and community agency, and a local system of interdisciplinary and cross-sectoral action interacting with each other. In other words, ICC is based on a specific conception of adaptive and empowered action delivered within a local system with different partners and leaded in part or totally by public HSCN. This middle-range theory, with its nine mechanisms, can be used in different settings – local areas and population – and can shed lights on the possible evolution of the HSCN and associated services.

### Three components for an ICC middle-range theory

The philosophy guiding ICC is not always clearly conceptualized in the selected texts. Nevertheless, some recurring points mentioned in several papers include the importance of understanding the area of intervention, recognizing the importance of temporality, and actualizing a learning culture. ICC represents a cultural change for the public HSCN towards a holistic approach to interdisciplinary and cross-sectoral care in the local area (integrating housing, municipal, community, school, etc.). It takes time to implement the approach, to get to know the local area, and to build trust with the people and the partners. In a health-care and social-care system dealing with demands, pressure to perform, and political or financial issues, taking into account temporality is a complex mechanism [19]. Moreover, this cultural change based on learning and innovation leads to managing differently and combining intervention strategies that are not often used together in other approaches: it is “a countercurrent practice that requires managers and decision-makers to be open to the fact that we have methods that go off the beaten track” [Translated from French, 44, p. 26]. The actualization of a learning culture takes on its full meaning in this context in which trials, innovative actions, and new interdisciplinary and cross-sectoral linkages are developed, requiring reflection on the targets or outcomes achieved, posing challenges in certain contexts or developing cultural sensitivity in relation to specific populations (e.g., migrants, refugees…) [41].

The professional and community agency at the heart of flexibility combines complementary intervention approaches (strength-based approach, empowerment, people-centered care, spatial and relational proximity) that must be supported by an adapted management of the public HSCN that understands the specific needs of ICC. Several texts analyzed mention this aspect of combining intervention approaches, i.e., the concomitant activation of several mechanisms. It is this combination that leads to significant and lasting outcomes in the local area, such as components of improvement of the social capital and empowerment of citizens, improvement of trust in others and in the community, cooperation and creation of a mutual-aid culture between neighbors, participation and increase in individuals’ social networks, proactivity of citizens to act by themselves on personal situations (e.g., mental health, loneliness) or collective situations (e.g., housing conditions, access to a food supply). In addition, it is important to highlight the case of Trieste [32], which is the only application that produced a longitudinal and statistical analysis, brought out three statistically significant items:

- Improved positive perception of the community, extending even to those not receiving services
- Decreased use of hospital services, including psychiatric services
- Recognition of people’s leadership in their community

Lastly, rooting ICC in the local area brings partners from different organizations together. The obvious conclusion is that various stakeholders from the same institution or from different organizations work with the same people in the local area. It is important to link them together, whether they are interveners or managers. The complex nature of the situations faced in a context in which each actor needs to achieve concrete and measurable results makes the two CMO configurations on interdisciplinarity and cross-sectorality important to deploy, even if sometimes difficult to operationalize, especially in a contact of centralized public HSCN [55,56,57]. The public HSCN cannot act alone in undertaking preventive action on well-being and health inequalities [58]. The local area is an ideal place to link up stakeholders close to people’s needs and issues [59].

### Limitations

The principal limitation of the study is related to the kind of papers found in the literature search. The sample consisted mainly of qualitative and mixed-methods papers, with no randomized-control trial. To the best of our knowledge, no study has analyzed the deployment of ICC in comparable local areas. Another limitation is that scant information on the deployment of action itself in the local area is provided in most of the cases [Appendix 1]. Also, the selected texts were limited in variety (4 cases = 19 texts out of 26), which gave an extensive comprehension of the context of theses cases, but is weaker in terms of the variety of settings encountered that could have increased the strength of our middle-range theory.

This synthesis has different strengths. First, we used rigorous methodology to perform the different steps of the review, with several communications back and forth between the main authors and the research committee. Second, we consulted and involved a consultative committee, especially at the beginning of the review. With the pandemic situation and the impact on health-care and social-care professionals, the committee was not involved in analyzing the results. The scientific literature search was done in English and French. In addition, the combination of this realist synthesis with a systematic review gives strength to the results.

### Implications for future research and for services

The identified outcomes were mainly qualitative, although some quantitative outcomes were reported in two cases (Trieste and Barcelona). These outcomes are relevant for understanding how actions on social determinants of health and on social capital—in addition to offering services in local areas—lead to care and intervention in the community, at an early stage, rather than in hospitals. It could be interesting to expand de longitudinal analysis of ICC practices, which could increase the strength of the ICC middle-range theory.

Place-based approaches are linked to ICC, but cannot always be considered as ICC, depending on the specific approach. The term was used in some of the papers analyzed, but it could be interesting to deepen the understanding and similarities between the two terms.

The intent of a realist synthesis is important to remember: “A realist approach to literature review addresses real-world issues through findings that “speak directly” to decisions regarding the creation or revision of social or health programs or policies” [11, p. 169]. In this regard, using the middle-range theory as a reflective framework to support the implementation or continuous improvement of ICC practices, to explain the approach, and to obtain a clear commitment of each partner (political, financial…) constitutes a new opportunity lacking in the scientific literature.

## Conclusion

This realist synthesis refines the model proposed by our team [1, Appendix 6 for the previous model] by identifying the underlying mechanisms and their contribution to outcomes. Using our middle-range theory for future research will provide a solid analytical framework for testing and refining the theory. The middle-range theory will also be useful in planning and supporting ICC implementation regardless of the target population or of the characteristics of the local area. ICC can be relevant in different settings to improve services if the philosophy of ICC is embedded in its actions. Lastly, it is hoped that longitudinal studies on ICC will add to the understanding of the generation of outcomes.

## Additional files

The additional files for this article can be found as follows:

10.5334/ijic.7042.s1 Appendix 1. Systematic review.

10.5334/ijic.7042.s2 Appendix 2. Search strategy.

10.5334/ijic.7042.s3 Appendix 3. Data Coding Guide.

10.5334/ijic.7042.s4 Appendix 4. List of included and excluded studies.

10.5334/ijic.7042.s5 Appendix 5. MMAT.

10.5334/ijic.7042.s6 Appendix 6. First ICC model.
