# Supplementary material for: Integrated Community Care Delivered by Public Health-Care and Social-Care Systems: Results from a Realist Synthesis
Source: Int J Integr Care. 2024 Feb 16;24(1):11. doi: 10.5334/ijic.7042 (PMC10870956; doi:10.5334/ijic.7042)
Supplement: Appendix 1. — Systematic review. [file ijic-24-1-7042-s1.pdf]

Appendix 1. Systematic review of ICC deployed by public health and social care networks

1. Context of the systematic review appendix

Before the realist synthesis was conducted, we carried out this systematic review with the aim to identify and characterize the integrated community care interventions delivered by public health-care and social-care networks as well as their related outcomes.

2. Systematic review method used

This systematic review was conducted using the Cochrane systematic review methodology (8) and was reported according to Preferred Reporting Items for Systematic Reviews and Meta-Analyses (PRISMA) guidelines (9). Since it is a phase in our realist synthesis project, the systematic review protocol was published as a part of the project (7). The protocol was registered on Open Science Framework under the hyperlink osf.io/5wy2e.

2.1. Research questions and eligibility criteria

Before carrying out the realist synthesis, we conducted this systematic review aimed at identifying and characterizing the type of intervention, the type population and area served, the issues and strength, the type of actors engaged in integrated community care interventions delivered by public health-care and social-care systems and the identified related outcomes. Eligibility criteria were presented using a PICOSS approach (See details in Table 1 and Table 2).

Table 1. Inclusion and exclusion criteria

Dimension	*Inclusion criteria*	*Exclusion criteria*
Type of study	Empirical studies published in any language for scientific papers; grey literature limited to English, French, and Italian	Studies with a narrow challenge found only in a specific local area and conference abstracts not followed up with a full text were excluded.
Range of years	Initially, published between January 2003 and March 2019; extended to February 2021.	---
Target population and area	Linked to target populations: all or major population groups (e.g. seniors, families, residents of social-housing complexes) and vulnerable or marginalized (material or social disadvantages) populations within specific territories. Large population groups were also considered (e.g., the elderly, vulnerable families, the disadvantaged, marginalized populations with complex problems, immigrants...)	Papers related to specific needs of a limited population were excluded, as these studies often focused on specific issues that might not have represented an approach that could be generalized across a population (e.g., retired soldiers living with post-traumatic stress disorder in a specific neighborhood).
Type of intervention	Integrated-community health and social care with the potential for universal, population-level reach (available and accessible to all in a specific local geographical area)	---
Leadership of intervention	Deployed by public health-care and social-care networks, with or without the collaboration of the community, other institutions, or private partners	Interventions deployed by the community or private sector

2.2. Information sources and literature search

The literature search for scientific studies was conducted in Ovid Medline, Elsevier Embase, EBSCOhost CINAHL, Ovid PsycINFO, Proquest - Sociological Abstracts, Web of Science Core collection, ÉRUDIT (requests in English and French), CAIRN (requests in English and French), and grey-literature sources coming from a network of world contacts.

The search strategy in electronic databases was designed with keywords (controlled and free vocabularies) of some PICOSS elements of Cochrane systematic reviews: community-based care, disadvantaged territory, integrated care, community-based health care, and community-based social care (Table 2). The search strategy was developed in Medline by an information specialist and revised by a second information specialist using the PRESS tool (Peer Review of Electronic Search Strategies) (10). Then, the search strategy was discussed with the review-team members through an iterative process before their final approval. The final version of the search strategy was translated for use in all the electronic databases mentioned from the respective inception to 09/02/2021. Appendix 2 contains the full search strategy.

After the literature search had been carried out in the electronic databases, a manual search was performed in the grey literature and in the bibliographic references of relevant papers to identify additional papers. We included both formal papers published in peer-reviewed journals and grey literature (any unpublished or non-peer-reviewed literature in the public domain).

Table 2. PICOSS of the review

Research question component	Characteristics	Definitions
**Population**	Description, characteristics	- all or major groups of the population (e.g., seniors, families, residents of social housing complexes)<br>- that is home to vulnerable or marginalized populations (material or social disadvantages) within specific local areas
**Interventions**	Content and terms of delivery	- Localized community-based health care<br>- Localized community-based social care<br>- or both integrated at the local area level<br>- deployed by public health or social care networks, with or without the collaboration of community or private partners
**Comparator**	Common alternatives	- Regular health care and social services
**Outcome(s)**	Types of results or outcomes produced	- Improved health and well-being of individuals and communities<br>- Improved health equity<br>- Improved community social capital, social networks, social cohesion<br>- Participation in co-production<br>- Improved accessibility, availability and continuity of health care and social care<br>- Action on social determinants of health
**Setting**		- Public providers<br>- Local area

2.3. Study selection

Various steps were followed to select papers. Two reviewers—members of the research team—participated in selecting studies. Step 1: The two reviewers involved in study selection performed a trial run on a sample of papers identified in the databases. This trial run allowed them to reach a common understanding of the selection criteria and to clarify them. Step 2: The two reviewers independently rated the papers and abstracts as Included, Excluded, or Unclear, based on their titles and abstracts. The items rated Included and Unclear went into the next step of assessment. Step 3: The two reviewers did independently the selection by full texts using the same criteria. The reviewers attempted to resolve any

disagreements at the end of each step by discussion to reach a consensus. If a consensus could not be reached, two other members of the review team were called in to reach a final decision.

2.4. Data extraction

Our data extraction adhered to the various guides and related systematic reviews indicated above (11–14). The reviewers initially extracted data from a sample of four studies, discussed the data with other members of the review team, and used these discussions to guide subsequent data extraction. The extracted data included the characteristics of the studies detailed in a coding grid (Appendix 3):

- Characteristics of the publication: design, country, year of publication, …
- Characteristics of the actors of the intervention: initiators, promotors, cross-sectoral partners, …
- Characteristics of the target population: for whom
- Characteristics of the intervention area: by whom
- Characteristics of the context of the intervention: Access and availability challenges, Continuity and quality challenges, issues, …
- Characteristics of the activities and strategy intervention: Health-care activities, Social-care activities, …
- Data related to empirical configurations context-mechanism-outcome (CMOc) – for the realist synthesis

The pair of reviewers used a custom-designed standardized data-extraction form to extract the data. The form allowed the reviewers to systematize the information collected and ensure a consistent approach. The electronic grid was tested on a few studies prior to its use for all publications included in the systematic review. Data extraction was verified iteratively by the research team. Selected studies were read twice and the relevant data transferred to the data-extraction form.

2.5. Assessment of study methodological quality

Two reviewers used the Mixed Methods Appraisal Tool (MMAT, version 2018) to assess the methodological quality for qualitative, quantitative, and mixed studies (15). The MMAT is a validated tool that presents specific assessment criteria for each study design. Disagreements between reviewers were resolved iteratively by members of the research team. Two preliminary questions on the clarity of the research questions and the collected data were answered. A score qualifying the methodological quality of each text was given based on the answers to the five other MMAT questions. This score was based on the number of "yes" responses for each of the questions. Thus, all "yes" responses equated to excellent quality. One "no" or "do not know" responses equated to good quality. Two "no" or "do not know" responses equated to adequate quality. Lastly, three or more "no" or "do not know" responses equated to poor quality.

2.6. Data synthesis

The data extracted related to the characteristics of the studies were statistically analyzed by our biostatistician in collaboration with the lead authors. The process of study selection was analyzed using frequency counts. The extracted data were analyzed using descriptive statistics in R software. Results were synthesized in tables and narrative form with respect to the studies, populations, interventions, and outcome characteristics.

3. Selection results

3.1. Search and selection results

Eighteen scientific papers and eight grey-literature documents were selected for the synthesis. The search flow diagram (Figure 1)—inspired by the PRISMA2020 model flow chart (9)—describes the search flow process and the results. Appendix 4 provides a description of these 26 publications. The quality of the 26 selected papers was assessed by two reviewers from the research team using the 2018 version of the MMAT tool (Appendix 5).

3.1.1 Scientific papers

Out of the 14,748 studies published between January 2003 and February 2021 (duplicate records = 5696; ineligible records = 9003), 52 papers were preselected for complete reading based on their titles and key words. There were 12 conference abstracts of potential relevance identified. Authors of these conference abstracts have been contacted in order to obtain a complete paper or a report. Three corresponding and reference authors (Eastwood, Dalton, and Di Monaco) transferred four additional papers. Authors of the other conference abstracts failed to respond, so their papers were excluded after a second attempt to contact them. Using the snowball method, four additional studies (other sources) were selected from the studies preselected. After complete reading of the papers and quality assessment, 18 scientific papers were selected for inclusion in the synthesis (16–33).

Figure 1. Flowchart of the systematic review

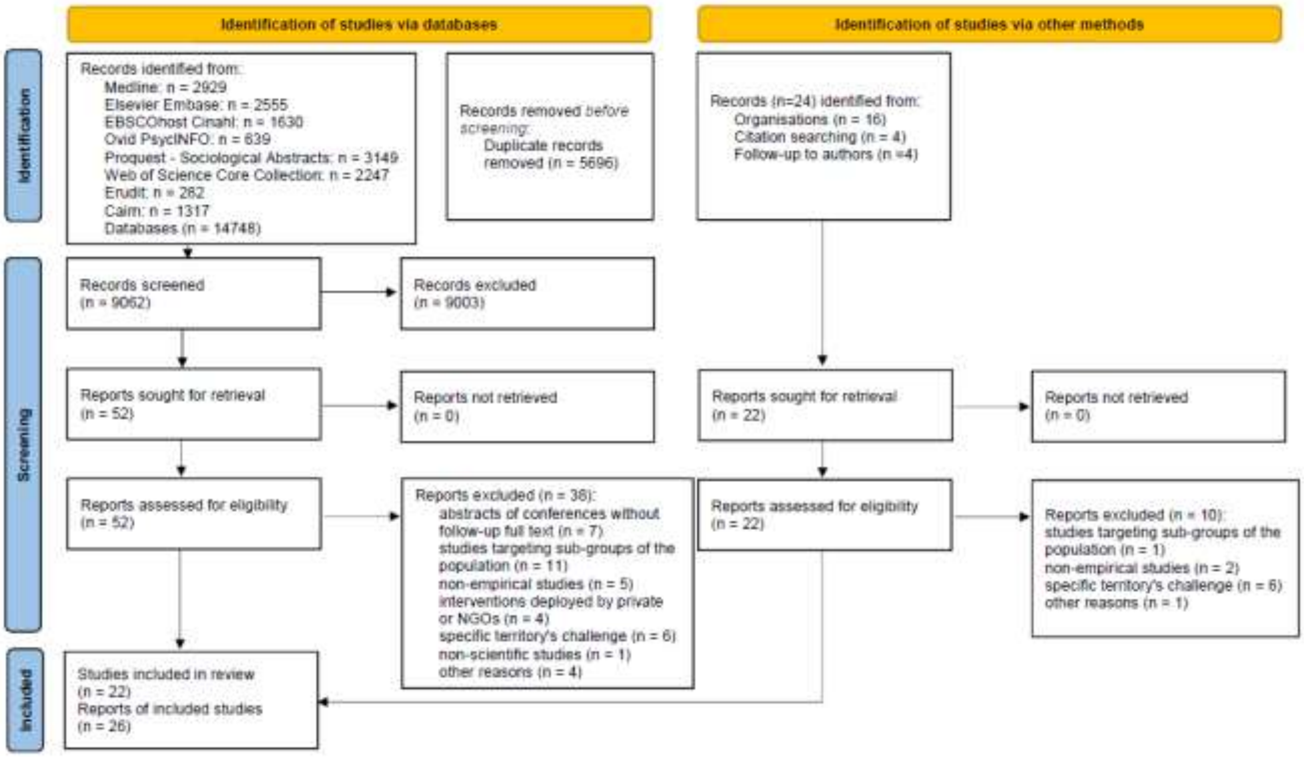

3.1.2. Grey literature

Initially, 18 texts published between January 2003 and February 2021 were identified in Google Scholar and by using the research team's international network of contacts (mainly in Canada, England, Italy, and Scotland) to supplement information in order to identify other relevant documents for the review. No duplicates were observed. Applying the snowball method to the selected grey-literature documents for their relevance to the realist review identified 2 additional documents for a total of 20. Reading the abstracts allowed us to select 17 publications. Applying the inclusion criteria (relevance and quality) resulted in eight grey-literature documents being retained (34–41); nine were excluded.

3.1.3. Evaluating the quality of the selected studies

After discussion between the quality evaluators and the whole team, the research team determined that two-thirds of the publications evaluated (i.e., 13 out of 16 scientific papers) were of very high quality (excellent or very good quality) (Appendix 5). The quality assessment criteria did not apply to two conference abstracts that were co-authored with associated scientific studies and included in the systematic review (22,33).

Only one publication was of medium or moderate (adequate) quality, based on the extent to which it met most of the assessment criteria, including the lack of a clear description of the data analysis method (17).

In contrast, two publications were considered to be of low or questionable quality, as they did not meet most of the criteria for assessing methodological quality (20,23). Neither article provided a clear description of the methods of data collection and analysis, although the qualitative approach used was appropriate for answering the research question.

3.1.4. Final selection

Of the 26 papers (18 scientific papers and 8 grey-literature publications) included in the systematic review, 8 with different designs relating to the same research and published by the same group of authors were treated concurrently in the analysis and synthesis. Two studies published by Eastwood et al. reporting a different but sequential multisectoral collaborative design process of interventions for vulnerable families were processed concurrently (one conference abstract (33) and one article (30)). In addition, three studies published by Dalton et al. were discussed concurrently (one conference abstract (22), one report (29), and one scientific paper (27)). A journal article (41) was also published concomitantly with the study published by Di Monaco et al. (26) on the Trieste case. Therefore, out of the 26 publications included in the systematic review, 4 were processed concurrently (27,29,33,41) with other publications. As a result, a total of 22 studies related to integrated community care and meeting the inclusion criteria were included in the systematic review (16–26,28,30–32,34–40).

3.2. Characteristics of the selected studies

Table 3 provides details on general characteristics of the selected studies. Consistent with the review inclusion criteria, the community-based and integrated health-care and/or social-care initiatives associated with 22 studies reported in the 26 publications included were deployed by the public health-care and social-care network, alone or in collaboration with community, private, political, institutional, or civic actors (Section 3.3 for details).

The selected studies came primarily from three countries: Australia (N=6) (17, 21, 27, 29–31), Canada (N=6) (15, 22, 34–36, 39), and Italy (N=5) (23, 25, 33, 37, 38). The remaining studies were from France (N=2) (18, 20), Japan (N=1) (17), Scotland (N=1) (25), and Spain (N=1) (20). The studies were mainly written in English

(N=16) (15–17, 19, 21–25, 27, 29–31, 33, 37, 38) or French (N=6) (18, 20, 34–36, 39). Four of the selected studies relate to community-based health care (18–20, 22), two to community-based social care (34, 35) and sixteen to integrated community care (15–17, 23–27, 29–31, 33, 36–39).

Most of the documents selected for the review were scientific papers (N=15) (15–20, 22–27, 29–31). Seven scientific reports from the grey-literature search were included (34–40). Lastly, two conference abstracts were processed concurrently with two scientific papers. Twelve studies took a qualitative-research approach (17, 24, 29–31, 33–39), five used mixed methods (15, 23, 25–27), and five opted for a descriptive study design (16, 18–20, 22).

Table 3. Characteristics of studies included (N=22)

Characteristics	N	Characteristics	No.
**Country of publication**			
Australia	6	**Language**	
Canada	6	English	16
Italy	5	French	6
France	2		
Japan	1	**Study design**	
Scotland	1	Qualitative study	12
Spain	1	Mixed design study	5
**Type of publication included (N=24; see note)**		Descriptive study	5
Scientific paper	15	**Primary intervention approach**	
Scientific report	7	Integrated community care	16
Conference abstract (see note)	2	Localized community health care	4
**Year of publication**		Localized community social care	2
Range	2005-2020		
Median	2017		

Note: Two conference abstracts were processed concurrently with a scientific paper or scientific report. Six of the seven scientific reports came from the grey-literature search.

3.3. Implementors characteristics

The stakeholders mainly identified in the studies that deployed ICC were (Table 4) ministries or public health-care entities (N=18) and ministries or public social-care entities (N=17). Half of the studies mentioned as implementors governmental or public organizations on the national, state, or regional level (N=11) and municipal or local governmental or public entities (N=9). Some studies mentioned public social-housing entities (N=6) or NGOs and community-based organizations (nonprofit cooperatives...) (N=5). Private corporations (family-practice groups, private clinics…) jointly deployed ICC in 3 cases. Other stakeholders acted more as partners in ICC, contributing in various ways.

The main partners involved in ICC identified in the review were ministries or public health-care entities (or integrated health and social care) (N=22); NGOs and community-based organizations (nonprofit cooperatives...) (N=20); municipal or local governmental or public entities (N=19); and ministries or public social-care entities (N=18). Moreover, three-quarters of the studies had cross-sectoral partners involved in their ICC: ministries or

public education entities (N=17); civic entities (neighborhood/seniors'/users' associations) (N=17); and public social-housing entities (N=14). Around half of the studies referred to partners in these sectors: private corporations (family-practice groups, private clinics…) (N=11), ministries or public entities in the area of public security (N=9); and governmental or public organizations on the national, state, or regional level (N=7). A quarter of the studies had these cross-sectoral partners involved in their ICC: Academic and research institutions (N=6), ministries or public entities in the areas of employment, poverty, or social solidarity (N=5); and social economy organizations (profit cooperatives…) (N=5). Lastly, other partners were involved in six cases (service users, families, or elected officials).

The professionals most frequently found involved in ICC were general practitioners (family physicians and paediatricians) (N=19); psychosocial workers (mental health, youth, elders, social workers…) (N=19); outreach workers (N=19); nurses (N=19); care-team managers (health and social care) (N=18); and others (mainly workers from other institutions (schools), non-profits, and volunteers) (N=18). In addition, certain categories of professionals were observed in about half of the cases such as health therapeutic services (physiotherapists, psychotherapists, occupational therapists...) (N=14); administrative and technical managers (N=12); medical specialists (N=10); and paramedics (N=8). Lastly, a few studies mentioned other kinds of professionals, such as researchers and student researchers (research and academic institutions) (N=5); neighbourhood pharmacists (N=4); community organizers and community-development workers (N=4).

Table 4. Characteristics of intervention actors

Characteristics	No. out of 22 studies	References
**The main actors deploying ICC**		
Ministries or public health-care entities	18	15-17, 20–25, 27, 29-31, 33, 36-39
Ministries or public social-care entities	17	16, 17, 21, 23-25, 27, 29-31, 33-39
Governmental or public organizations on the national, state, or regional level	11	15-17, 19-22, 27, 29-31
Municipal or local governments or public entities	9	16, 18, 20, 23, 25, 33, 37-39
Public entity of social housing	6	24,26,34,38-40
NGOs and community-based organizations (nonprofit cooperatives...)	5	17, 29-31, 39
Private corporations (family-practice groups, private clinics…)	3	15, 18, 19
**The intersectoral actors most frequently engaged in ICC**		
Ministries or public health-care entities	22	15-25, 27, 29-31, 33-39
NGOs and community-based organizations (nonprofit cooperatives...)	20	15-25, 27, 29-31, 33-39
Municipal or local governments or public entities	19	16-21, 23, 25, 27, 29-31, 33-39
Ministries or public social-care entities	18	16, 17, 21-25, 27, 29-31, 33-39

Characteristics	No. out of 22 studies	References
Ministries or public education entities	17	17, 19, 21-23, 25, 27, 29-31, 33-39
Civic entities (neighborhood/seniors'/users' associations)	17	15-17, 19, 21, 23, 25, 27, 29-31, 33-38
Public social-housing entities	14	17, 20, 23, 25, 29-31, 33-39
Private corporations (family-practice groups, private clinics…)	11	15-19, 22, 29-31, 35, 36
Ministries or public entities in the area of public security	9	22, 23, 25, 33-35, 37-39
Governmental or public organizations on the national, state, or regional level	7	15, 16, 18-20, 26, 27
Academic and research institutions	6	19-21, 26, 34, 35
Other (service users, families, or elected officials)	6	17, 20, 24, 29-31
Ministries or public entities in the areas of employment, poverty, or social solidarity	5	17, 29-31, 39
Social-economy organizations (nonprofit cooperatives…)	5	23, 25, 33, 37, 38
**The professionals who collaborate most frequently in the context of ICC**		
General practitioners (family physicians and pediatricians)	19	16-26,28,30-32,34,35,38,39
Psychosocial workers (mental health, youth, elders, social workers…)	19	16-18,20,23,24,26-28,30-32,34-40
Outreach workers	19	16-18,20,23,24,26–28,30-32,34-40
Nurses	19	16-26,28,30-32,34,35,38,39
Care-team managers (health and social care)	18	16-18,20,22,24,26,28,30-32,34-40
Other (mainly workers from other institutions (schools), nonprofits, and volunteers)	18	16-18,20,21,24-26,30-32,34-40
Health therapeutic services (physiotherapists, psychotherapists, occupational therapists...)	14	16,17,19-24,26,28,34,37-39
Administrative and technical managers	12	16,18-22,28,30-32,37,40
Medical specialists	10	16-21,30-32,40
Paramedics	8	17,19,21,24,26,34,38,39
Researchers and student researchers (research and academic institutions)	5	20,21,27,28,36
Neighborhood pharmacists	4	16,19-21
Community organizers and community-development workers	4	35-37,40

3.4. Characteristics of the population and local intervention area

The target populations in the local areas where ICC has been deployed were socially vulnerable individuals (single, divorced, or widowed) (N=20) and economically deprived individuals (low income, material poverty…) (N=19) (see Table 5). These general population characteristics were combined with other characteristics. In three-quarters of the cases, the target populations included people not reached by conventional health-care and social-care services and with chronic or complex conditions (N=16); families living in vulnerable situations with multiple and complex needs (N=15); or people with a mental-health problem (N=14). Around half of the studies had specific subgroups as the target population: cultural or ethnic groups (immigrants, indigenous, refugees, asylum seekers, racial/ethnic minorities) (N=13); the entire community (N=12), elderly population (N=12); people with major health problems and long-term illnesses (N=11); or people with a functional limitation (N=8).

Table 5. Characteristics of the targeted populations

Characteristics	No. out of 22	References
Socially vulnerable people (single, divorced, or widowed)	20	17,18,20-26,28,30-32,34-40
Economically deprived people (low income, material poverty…)	19	18,20-26,28,30-32,34-40
People not reached by conventional health-care and social-care services and with chronic or complex conditions (marginalized, homebound patients, the homeless, the roofless, illegal drug users, sex-trade workers...)	16	16,18,21-26,28,34-40
Families living in vulnerable situations with multiple and complex needs (children, mother under 20 years old, one parent with a dependency, mental health or intellectual disability problem, abuse/neglect, vulnerable parents, families of social housing)	15	18,22-24,26,28,30-32,35-40
People with a mental health problem	14	18,23,24,26,30-32,34-40
Cultural or ethnic group (immigrant, indigenous, refugee, asylum seeker, without permanent residence, without status, precarious immigration status, racial/ethnic minority)	13	18,21,24-26,30-32,34-36,38,39
The entire community	12	19-21,24-26,34-39
Elderly population (with social and economic deprivation, with or no illnesses and disabilities, with complex needs)	12	17,19-22,24-26,28,34,38,39
People with major health problems and long-term illnesses (chronically ill or bedridden people)	11	16,17,21,22,24-26,28,34,38,39
People with a functional limitation (disability or loss of autonomy)	8	17,19,24-26,34,38,39

The local areas in the studies were all places where people resided and lived on a daily basis (N=22) (Table 6). Other characteristics were present to some degree in all cases: availability of community resources (health and social care, social housing, meeting places...) (Yes=20, Some=2); economic services available in the area (restaurants, bars, banks, shops, supermarkets...) (Yes=18, Some=4) and availability of other public services (post office, police, schools, libraries, transportation, parks, paths…) (Yes=17, Some=5). Local area services were a little less frequent for these characteristics: availability of public health-care and social-care services (hospitals, health centers...) (Yes=10, Some=12); and availability of other health and social services (pharmacies, private clinics...) (Yes=8, Some=14).

Other characteristics were frequently mentioned but not in all the studies: urban and multiproblem local areas (deprived neighbourhoods, urban metropolitan areas…) (Yes=17); and densely populated (Yes=15, Some=1) and underserved communities (disadvantaged with respect to medical and social care) (Yes=11, Some=9). Lastly, around half of the studies mentioned characteristics related to rural or remote areas (distant and vast, small communities, isolated…) (Yes=9); and unpopulated (Yes=7, Some=2). Geographically perceived identity or heritage was mentioned in five studies (Yes=5).

Table 6. Intervention-area characteristics

Characteristics		No. out of 22	References
Place where people resided and lived on a daily basis	Yes	22	16-26,28,30-32,34-40
	Some	--	
Availability of community resources (health and social care, social housing, meeting places...)	Yes	20	16,18-20,22-26,28,30-32,34-40
	Some	2	17,21
Economic services available in the area (restaurants, bars, banks, shops, supermarkets...)	Yes	18	16,19-26,28,31,34-40
	Some	4	17,18,30,32
Availability of other public services (post office, police, schools, libraries, transportation, parks, paths…)	Yes	17	16,19-22,24-26,28,31,34-40
	Some	5	17,18,23,30,32
Available public health and social care services (hospitals, health centres, ...)	Yes	10	16-19,21,23,30-32,37
	Some	12	20,22,24-26,28,34-36,38-40
Availability of other health and social services (pharmacies, private clinics, ...)	Yes	8	16,19-21,31,35-37
	Some	14	17,18,22-26,28,30,32,34,38-40
Underserved communities (medically and social care disadvantaged communities)	Yes	11	17,21,23,24,26,31,34-36,38,39
	Some	9	18,19,22,25,28,30,32,37,40
Urban and multiproblem local areas (deprived neighborhoods, urban metropolitan area…)	Yes	17	16,18,20,21,24-26,30-32,34-40
	Some	--	
Densely populated	Yes	15	18,20,21,24-26,30-32,34-36,38-40
	Some	1	37
Rural or remote areas (distant and vast, small communities, isolated…)	Yes	9	17-19,22,23,28,30-32
	Some	--	

Unpopulated	Yes	7	17-19,23,30-32
	Some	2	27,28
Geographically perceived identity or heritage	Yes	5	19,20,27,28,37
	Some	--	

3.5. Strengths and issues related to health and social-care services

Analysis of the texts revealed various strengths and issues, as given below, beginning with the issues related to access, availability, quality, and continuity and followed by the strengths and issues related to implementation, practice, and management.

In regard to access and availability (Table 7), all or almost all studies identified unanswered health-care and social-care needs (N=22); access problems (too diverse, difficult to access, complex to understand, substantial structural barriers…) (N=21); and few service points available (lack of service providers…) (N=20). The studies frequently brought up other issues: failure to take care of patients in vulnerable health situations (N=18); funding challenges (N=17); lack of human resources for outreach services (N=15); and health care and social care geographically distant from the people the health system has difficulty reaching (N=13). Lastly, fewer than half of the studies mentioned other issues: limited number of social, training, and recreational spaces (N=8); strategic and governance managers lack of knowledge or understanding about services (N=7); challenge of staff renewal (N=4); not enough opportunities for professional help support and collective reflections (N=3); lack of support from local elected representatives (N=2); and inadequate and insecure care environments for vulnerable people with chronic and complex needs and a criminal record (N=1).

The issues related to continuity and quality (Table 8) related mainly to fragmented health care and social care delivery (N=22); lack of community-based interventions (N=20); access to discontinuous health-care and social-care pathways (n=19); limits of single-discipline practices (N=19); access to episodic or low-quality care (N=18); and challenges to patient engagement (N=18). Half of the studies mentioned these issues: reconciling the complex needs of patients with the requirements of professionals and health authorities (N=11); inflexible intervention practices (N=10); and limited competences for acting on complex realities (N=10). Some studies mentioned unclear knowledge of principles and strategies of proximity intervention (N=7) and inflexible management practices or accountability poorly adapted to the specificity of proximity interventions (N=6).

Table 7. Issues related to health-care and social-care access, availability, continuity, and quality

Access and availability	No. out of 22	References
Unanswered health-care and social-care needs	22	16-26,28,30-32,34-40
Access problems (too diverse, difficult to access, complex to understand, substantial structural barriers…)	21	16-24,26,28,30-32,34-40
Few service points available (lack of service providers…)	20	17-26,28,30-32,34-36,38-40
Failure to take care of patients in vulnerable health situations (elderly and disabled persona, chronically ill, bedridden people...)	18	17,18,22-26,28,30-32,34-40
Funding challenges	17	17-19,21,23-26,30-32,34-36,38-40
Lack of human resources for outreach services (outreach health-care and social-care workers)	15	18,19,21,23,24,26,30-32,34-36,38-40
Health care and social care geographically distant from the people the health system has difficulty reaching (unattached patients, marginalized, with complex or highly specialized needs, living on the street or homeless, illegal immigrants, paperless persons, drug addicts, administrative hassles, language barriers, financial issues…)	13	16,22-24,26,28,34-40
Limited number of social, training, and recreational spaces	8	24-26,34,36-39
strategic and governance managers lack of knowledge or understanding about services	7	20,21,23,27,28,35,36
Challenge of staff renewal (unfavourable working conditions for professionals)	4	19,21,36,37
Not enough opportunities for professional help support and collective reflections	3	21,36,37
Lack of support from local elected representatives (financial, materials…)	2	20,21
Inadequate and insecure care environment for vulnerable people with chronic and complex needs and a criminal record (colocation of common services, such as public health-care clinics sharing the same building with police; one building housing many different agencies and services)	1	23
**Continuity and quality**	**No. out of 22**	
Fragmented health care and social care delivery (uncoordinated care between primary-care services in the same area: integration multiple professionals and partners, hospitals, emergency rooms, pharmacies, and other sources of care)	22	16-26,28,30-32,34-40
Lack of community-based interventions	20	17-24,26,28,30-32,34-40
Access to discontinuous health-care and social-care pathways (continuity: financial and operational challenges)	19	17-19,21-26,28,30-32,34-36,38-40

**Access and availability**	**No. out of 22**	**References**
Limits of single-discipline practices (complex needs)	19	16-19,21,22,24,26,28,30-32,34-40
Access to episodic or low-quality care (quality)	18	16-18,22-26,28,30-32,34-36,38-40
Challenges to patient engagement	18	16-18,21-24,26,28,30-32,34-36,38-40
Reconciling the complex needs of patients with the requirements of professionals and health authorities	11	16,18,20-23,25,28,30-32
Inflexible intervention practices (not adapted to the realities and needs)	10	23-26,34-39
Limited competences to act on complex realities (individuals and groups)	10	18,21-23,28,30-32,35,36
Unclear knowledge of principles and strategies of proximity intervention	7	21,23,27,28,35-37
Inflexible management practices or accountability poorly adapted to the specificity of proximity interventions	6	20,21,27,28,35,36

The analysis of the studies brought out strengths and issues related to implementation, practices, and management (Table 8). In some studies, a contextual item was considered a strength; for others, an issue. Almost all studies mentioned organizing health care and social care organization to match realities and needs (N=20) as strengths. Community-based care (strength – S=18, Issue – I=4); location of health-care and social-care delivery (S=18); mobilization of resources (S=18, I=3); interdisciplinary collaboration (S=17, I=4), cross-sectoral partnerships (S=17, I=3); and personalized and flexible interventions (S=15, I=4) were mostly considered as characteristics related to strengths. Understanding lifestyles and population needs (S=12, I=5); sustainability of health-care and social-care delivery (S=11, I=4); and understanding of the intervention territory (S=11, I=4) were considered as a strength by two-thirds of the studies and as an issue by one-third. Temporality (S=10, I=3) was seen as both a strength and an issue. Some studies mentioned replacement of health-care and social-care workers as a strength (S=4).

Table 8. Strengths and issues related to implementation, practices, and management

Characteristics		No. out of 22	References
Organizing health care and social care to match realities and needs (development of a continuum of care and services, organizational innovation, and cultural change: patient education, care coordination, and preventive care provided by a team…)	**Strength**	20	16-21,23-26,30-32,34-40
	**Issue**	--	
Community-based care (integration of health care and social care, coordination between systems covering different geographical areas or offering different levels of service intensity…)	**Strength**	18	16-19,21,24-26,30-32,34-40
	**Issue**	4	20,23,27,28
Location of health-care and social-care delivery (accessible, user-friendly, and safe environment for users…)	**Strength**	18	16-19,21,24-26,30-32,34-40
	**Issue**	--	
Mobilization of resources (financial, material, and technical)	**Strength**	18	16-19,21,23,24,26,30-32,34-40
	**Issue**	3	20,27,28
Interdisciplinary collaboration	**Strength**	17	16–19,21,24,26,30-32,34-40
	**Issue**	4	20,23,27,28
Cross-sectoral partnerships (involvement of several sectors, stable partnerships, active community participation in deciding on care provision and resource allocation, patient engagement, cross-sectorial coordination, establishment of facilitating structures…)	**Strength**	17	16–19,21,24,26,30-32,34-40
	**Issue**	3	20,27,28
Personalized and flexible interventions (people-centered care, outreach approach...)	**Strength**	15	17,18,24-26,30-32,34-40
	**Issue**	4	20,23,27,28
Understanding lifestyles and population needs (residents' needs, professional expectations, social capital, civic engagement, empowerment, demographic structure and dynamics…)	**Strength**	12	16,18,24,26,30-32,34-36,38,39
	**Issue**	5	19,21,23,37,40
Sustainability of health care and social care delivery	**Strength**	11	19,21,24-26,34-36,38-40
	**Issue**	4	16,20,27,28
Understanding of the intervention territory (geographic, experienced, perceived, and conceived)	**Strength**	11	18,24,26,30-32,34-36,38,39
	**Issue**	4	19,21,37,40
Temporality (service delivery, patient involvement...)	**Strength**	10	17,24,26,34-40
	**Issue**	3	16,22,28
Replacement of health-care and social-care workers (professionals and managers)	**Strength**	4	19,21,36,37
	**Issue**	--	

3.6. Management and intervention activities in the context of ICC

The analysis of the studies identified various types of activity (Table 9). Some were observed for both health care and social care, while others only for one or the other. When both social care and health care were mentioned in the selected studies, connection to intervention resources (Social care – SC= 18, Health care – HC=21); preventive care (SC=18, HC=19); information and awareness (SC=18, HC=18); self-care/empowerment support (SC=16, HC=19); consultation (SC=15, HC=20); and care promotion (SC=18, HC=18) came up frequently. The following only occurred with reference to social care: psychosocial support and coaching (SC=17); rehabilitation assistance (SC=15); empowerment in life skills (SC=14); and support for collective mobilization (SC=12). These activities only occurred with reference to health care: medical and nursing care (HC=19); tracking and testing (HC=15); and home health care (HC=9). Lastly, 4 cases were related to pleading and advocacy (SC=3, HC=1).

Table 9. Most frequently observed health-care and social-care activities in the context of ICC

Activities		No. out of 22	References
Connection to intervention resources (referrals to other specialty services intern and extern)	Social care	18	16-18,22,24-26,28,30-32,34-40
	Health care	21	16-26,28,30-32,34,35,37-40
Preventive care	Social care	18	16-18,22,24-26,28,30-32,34-40
	Health care	19	16-18,20-24,26,28,30-32,34,35,37-40
Information and awareness (care professionals and target population)	Social care	18	16-18,22,24-26,28,30-32,34-40
	Health care	18	16-18,20-22,24,26,28,30-32,34,35,37-40
Self-care and empowerment support	Social care	16	16-18,24-26,30-32,34-40
	Health care	19	16-21,23-26,30-32,34,35,37-40
Consultation	Social care	15	16-18,24-26,30-32,34-40
	Health care	20	16-26,28,30-32,34,35,38-40
Care promotion	Social care	18	16-18,22,24-26,28,30-32,34-40
	Health care	18	16-18,20-24,26,28,30-32,34,35,37-39
Medical and nursing care	Social care	--	
	Health care	19	16-26,28,30-32,34,35,38,39
Psychosocial support and coaching (group or individual)	Social care	17	16,18,22,24-26,28,30-32,34-40
	Health care	--	
Rehabilitation assistance	Social care	15	16-18,24-26,30-32,34-36,38-40
	Health care	--	
Tracking and testing	Social care	--	
	Health care	15	16-18,20,21,23-26,30-32,34,38,39
Empowerment in life skills	Social care	14	18,24-26,30-32,34-40
	Health care	--	
Support for collective mobilization (social or recreational)	Social care	12	16,17,24-26,34-40
	Health care	--	
Home health care	Social care	--	
	Health care	9	16-18,24-26,30-32
Pleading and advocacy	Social care	3	35,36,40

(4). Analysis of the results

4.1. Summary of evidence

Paper selection was limited by three important criteria: the local area, the target population (many papers with very specific target populations), and an intervention deployed by the public health-care and social-care network. Most of the selected literature came from four cases: one from Trieste in Italy (5 papers), two in Australia (4 papers and 5 papers), and one in Canada (2 papers). Some cases focused more on intervention, while others were more focused on governance and service organization. The latter cases provided little information about the interventions as such. Also, this review combines peer-reviewed papers and grey literature. It itself, it is a strength, be we found a smaller number of grey literature texts than peer-reviewed papers. The key words used could be an explanation, since terms related to ICC are not always constant. We found the following: 1 – ICC were deployed by public health-care and social-care networks with cross-sectoral action; 2 - targeted population are consistent with the objective of reducing social and health inequities; 3 - identified issues were consistent with the issues of access, availability, continuity, and quality of health care and social care; and 4 - management and intervention activities were linked with a holistic view of ICC services.

4.2. Explanatory hypotheses supported by the literature

The ICC practices identified were deployed by health-care and social-care networks. While other partners were involved in the deployment of half the cases, cross-sectoral action was seen in the local area. This set of organizations and stakeholders is consistent with the ICC objective of addressing the social determinants of health and social capital in the local area, which requires a broad-based approach.

The main target populations of the ICC reported on were consistent with the objective of reducing social and health inequities. These populations were mainly socially vulnerable, remote, or unreached by services, economically impoverished, with chronic health problems or mental-health issues. Such populations frequently need more care and services than advantaged populations (42,43). Taking preventive action with these populations can improve their living conditions and reduce expenditure by health-care and social-care systems (44).

The main issues identified in relation to accessibility, availability, continuity, and quality are consistent with our theoretical contextual elements that led to the establishment of ICC (1). Unanswered health-care and social-care needs, access problems, and few service points in local area are key points linked with access to and availability of services in the local area. Quality and continuity issues were linked mainly with fragmented health care and social care delivered, lack of community-based interventions, access to discontinuous health-care and social-care pathways, limits of single-discipline practices, access to episodic and low-quality care, and challenges to patient engagement. All these challenges for public health care and social care are the driving forces behind the establishment of ICC. These issues were often mentioned as general barriers to care and services (5,42).

The strengths and issues raised in the systematic review related to the deployment, practices, and management of ICC concern fundamental components of the ICC model presented by Thiam et al. (1). Matching care organization with realities and needs, community-based care, location of health-care and social-care delivery, mobilization of resources in proximity, interdisciplinary collaboration, cross-sectoral partnership, and personalized and flexible intervention were mostly considered as characteristics related to strengths. These contextual elements are the foundation to the mechanisms presented in the realist synthesis that this systematic review support.

Lastly, both health care and social care were frequently mentioned in connection with intervention resources, preventive care, information and awareness, self-care and empowerment support, consultation and care promotion as the most frequent management and intervention activities. These activities are linked with a holistic view of ICC services, not centered solely on direct care, but open to a larger definition of health and well-being and health equity principles (43,45,46).

4.3. Limitations and strengths

The principal limitation of the study is related to the kind of papers retrieved in the literature search. The sample was comprised of mainly qualitative and mixed-methods papers, with no randomized-control trial. In addition, the number of strong cases retrieved is limited. To the best of our knowledge, no study has been undertaken to analyze the deployment of ICC in comparable local areas. Another limitation is that scant information on the deployment of action itself in the local area is provided in these cases. The selected studies primarily describe the integration, management, and governance processes leading to the deployment of ICC in a local area. One case in Australia, one in Japan, and one in France were selected for their relevance in describing the governance and management changes to integrate care at the local area level. Furthermore, the data presented are to be considered with the realist synthesis.

This review has different strengths. First, we used rigorous methodology to perform the different steps of the review, with several communications back and forth between the main authors and the research committee. Thus, our results are both comprehensive and reproducible. Second, we consulted and involved a consultative committee, especially at the beginning of the review. With the pandemic situation and the impact on health-care and social-care professionals, the committee was not involved in analyzing the results. The scientific literature search was done in English and French. In addition, the combination of this systematic review with a realist synthesis gives strength to the results.

5. Conclusion

Although the consulted literature is limited and included a small number of relevant studies, we found that the characteristics and contextual elements of ICC selected in the review were consistent with ICC model (1). We also succeeded in listing and categorizing different issues, strengths, and activities. Our findings could be useful in underscoring the importance of adapting care services to the local area and populations that are hard to reach or impoverished.

ICC is an innovation with great potential for addressing conditions leading to social and health inequalities. These practices, however, require a different management and intervention philosophy, as well as a posture and action aimed at integrating services and professionals in cross-sectoral action. These components are explored in the middle-range theory emerging from the realist synthesis.

6. References

1. Thiam Y, Allaire JF, Morin P, Hyppolite SR, Doré C, Zomahoun HTV, et al. A Conceptual Framework for Integrated Community Care. Int J Integr Care. 2021 Feb 10;21(1):5.

2. Roeg D, van de Goor I, Garretsen H. Towards Structural Quality Indicators for Intensive Community-Based Care Programmes for Substance Abusers. Community Ment Health J. 2008 Dec;44(6):405–15.

3. Roeg D, van de Goor I, Garretsen H. Towards quality indicators for assertive outreach programmes for severely impaired substance abusers: concept mapping with Dutch experts. Int J Qual Health Care. 2005 Jun 1;17(3):203–8.

4. Larivière C. Les risques de la nouvelle gestion publique pour l'intervention sociale [The risks of the New Public Management to social intervention] [Internet]. Premier congrès international francophone du service social; 2005 Jul [cited 2020 Jun 16]. Available from: http://www.rrasmq.com/documents/Activites/RN_2010_Les-risques-NGP.pdf

5. Levesque JF, Harris MF, Russell G. Patient-centred access to health care: conceptualising access at the interface of health systems and populations. Int J Equity Health. 2013;12(1):18.

6. Doré C, Lévesque N, Hyppolite SR, Maillet L, Bourque D, Maltais D, et al. L'intervention de proximité : proposition d'un dispositif de reddition de comptes pertinent [Proximity intervention: proposal for a relevant accountability system]. Nouvelles pratiques sociales. 2022, 33(1): 112-133.

7. Allaire JF, Thiam Y, Morin P, Zomahoun HTV, Rheault N, Lacasse F, et al. Integrated Community Care Delivered by Public Health Care and Social Care Systems: Protocol for a Realist Synthesis. Int J Integr Care. 2021 Oct 26;21(4):2.

8. Higgins J, Thomas J, Chandler J, Cumpston M, Li T, Page M, et al., editors. Cochrane Handbook for Systematic Reviews of Interventions Version 6.2 (updated February 2022) [Internet]. Cochrane; 2022 [cited 2022 Apr 25]. Available from: https://training.cochrane.org/handbook

9. Page MJ, McKenzie JE, Bossuyt PM, Boutron I, Hoffmann TC, Mulrow CD, et al. The PRISMA 2020 statement: an updated guideline for reporting systematic reviews. BMJ. 2021 Mar 29;n71.

10. McGowan J, Sampson M, Salzwedel DM, Cogo E, Foerster V, Lefebvre C. PRESS Peer Review of Electronic Search Strategies: 2015 Guideline Statement. J Clin Epidemiol. 2016 Jul;75:40–6.

11. Wong G, Greenhalgh T, Westhorp G, Buckingham J, Pawson R. RAMESES publication standards: realist syntheses. BMC Med. 2013;11:21.

12. O'Dwyer LA, Baum F, Kavanagh A, Macdougall C. Do area-based interventions to reduce health inequalities work? A systematic review of evidence. Crit Public Health. 2007 Dec 1;17(4):317–35.

13. Institut national d'excellence en santé et en services sociaux (Québec), Renaud J, Martin V, Dagenais P. Les normes de production des revues systématiques: guide méthodologique [Production Standards for Systematic Reviews: A Methodological Guide]. Montréal: Institut national d'excellence en santé et en services sociaux (INESSS); 2013.

14. Hudon C, Chouinard MC, Pluye P, El Sherif R, Bush PL, Rihoux B, et al. Characteristics of Case Management in Primary Care Associated With Positive Outcomes for Frequent Users of Health Care: A Systematic Review. Ann Fam Med. 2019 Sep;17(5):448–58.

15. Hong QN, Fàbregues S, Bartlett G, Boardman F, Cargo M, Dagenais P, et al. The Mixed Methods Appraisal Tool (MMAT) version 2018 for information professionals and researchers. Educ Inf. 2018 Dec 18;34(4):285–91.

16. Shukor AR, Edelman S, Brown D, Rivard C. Developing Community-Based Primary Health Care for Complex and Vulnerable Populations in the Vancouver Coastal Health Region: HealthConnection Clinic. Perm J. 2018;22:18–010.

17. Hatano Y, Matsumoto M, Okita M, Inoue K, Takeuchi K, Tsutsui T, et al. The Vanguard of Community-based Integrated Care in Japan: The Effect of a Rural Town on National Policy. Int J Integr Care Electron Resour. 2017 Apr 27;17(2):2.

18. Tennant E, Miller E, Costantino K, De Souza D, Coupland H, Fotheringham P, et al. A critical realist evaluation of an integrated care project for vulnerable families in Sydney, Australia. BMC Health Serv Res. 2020 Oct 31;20(1):995.

19. Hamiti A. Territorialisation des soins primaires : initiatives des collectivités et des professionnels de santé sur le Massif central pour une autre gouvernance [Territorialisation of primary care: initiatives by local authorities and health professionals in the Massif Central for a different governance]. Norois. 2014;233(4):55–66.

20. Segura A, Miller FA, Foz G, Oriol y Bosch A. Towards unity for health in the Barceloneta: An innovative experience in community-based primary health care. Educ Health Change Learn Pract [Internet]. 2007;20(2). Available from: http://www.embase.com/search/results?subaction=viewrecord&from=export&id=L47516830

21. Fournier C. Concevoir une maison de santé pluri-professionnelle : paradoxes et enseignements d'une innovation en actes [Designing a multi-professional health centre: paradoxes and lessons learned from an innovation in action]. Sci Soc Santé. 2014;32(2):67–95.

22. Dalton H, Perkins D, Goodwin N, Hendry A, Read D, Booth A, et al. Using the Project Integrate Framework for assessing progress towards care integration: results from a formative evaluation of a complex intervention in Central Coast Local Health District, NSW Australia. Int J Integr Care IJIC. 2018;18:1–2.

23. Self B, Peters H. Street outreach with no streets: in a new take on a proven program, a rural street nurse is taking an outreach program to the highways and byways of the B.C. interior, reaching people who otherwise might not have adequate access to services. Can Nurse. 2005;101(1):20–4.

24. Castriotta L, Giangreco M, Cogliati-Dezza MG, Spano M, Atrigna E, Ehrenfreund C, et al. Measuring the impact of a social programme on healthcare: a 10-year retrospective cohort study in Trieste, Italy. BMJ Open. 2020 Jul 23;10(7):e036857.

25. Leask CF, Bell J, Murray F. Acceptability of delivering an adapted Buurtzorg model in the Scottish care context. Public Health. 2020 Feb;179:111–7.

26. Di Monaco R, Pilutti S, d'Errico A, Costa G. Promoting health equity through social capital in deprived communities: a natural policy experiment in Trieste, Italy. SSM - Popul Health. 2020 Dec;12:100677.

27. Dalton H, Read DMY, Booth A, Perkins D, Goodwin N, Hendry A, et al. Formative Evaluation of the Central Coast Integrated Care Program (CCICP), NSW Australia. Int J Integr Care. 2019 Aug 27;19(3):15.

28. Read DMY, Dalton H, Booth A, Goodwin N, Hendry A, Perkins D. Using the Project INTEGRATE Framework in Practice in Central Coast, Australia. Int J Integr Care. 2019 Jun 21;19(2):10.

29. Dalton H, Read D, Handley T, Booth A, Davies K, Goodwin N, et al. Central Coast Integrated Care Program : Formative Evaluation Report [Internet]. Centre for Rural and Remote Mental Health (CRRMH); 2018 p. 48. Available from: https://www.crrmh.com.au/content/uploads/CCICP20Evaluation20Report20180313_FINAL.pdf

30. Eastwood J. Designing an Integrated Care Initiative for Vulnerable Families: Operationalization of realist causal and programme theory, Sydney Australia. Int J Integr Care IJIC. 2018;18:1–2.

31. Eastwood J, Barmaky S, Hansen S, Millere E, Ratcliff S, Fotheringham P, et al. Refining Program Theory for a Place-Based Integrated Care Initiative in Sydney, Australia. Int J Integr Care Electron Resour. 2020 Sep 28;20(3):13.

32. Eastwood JG, Dewhurst S, Hansen S, Tennant E, Miller E, Moensted ML, et al. Care Coordination for Vulnerable Families in the Sydney Local Health District: What Works for Whom, under What Circumstances, and Why? Int J Integr Care. 2020 Dec 2;20(4):22.

33. Eastwood J, Miller E. The Healthy Homes and Neighbourhoods Integrated Care Initiative. Int J Integr Care IJIC. 2018;18:1–2.

34. Bricocoli M, Marchigiani E. Growing old in cities. Council housing estates in Trieste as laboratories for new perspectives in urban planning. Eur Spat Res Policy. 2012;19(1):49–64.

35. Morin P, Ducreux E, Allaire JF, Bossé PL, Gagné AA. Évaluation du déploiement des intervenants dédiés et des changements associés au sein de l’équipe-quartier [Evaluation of the deployment of dedicated workers and associated changes in the neighbourhood team]. Sherbrooke, Canada: Institut universitaire de première ligne en santé et services sociaux du Centre intégré universitaire en santé et services sociaux de l’Estrie – Centre hospitalier universitaire de Sherbrooke; 2017 p. 56.

36. Morin P, Benoît M, Dallaire N, Doré C, LeBlanc J. L’Intervention de quartier au CSSS-IUGS [Neighbourhood intervention at the CSSS-IUGS]. Sherbrooke, Canada: Centre de santé et de services sociaux - Institut universitaire de gériatrie de Sherbrooke.; 2011.

37. Allaire JF, Morin P, Maltais D, Doré C. Évaluation de l’implantation de l’équipe de proximité de Lac-Mégantic [Evaluation of the implementation of the Lac-Mégantic proximity team]. Institut universitaire de première ligne en santé et services sociaux du Centre intégré universitaire en santé et services sociaux de l’Estrie – Centre hospitalier universitaire de Sherbrooke; 2021.

38. Bifulco L, Bricocoli M, Monteleone R. The reflective Institution and the city. Space based policies upsetting organisations in Trieste. In Politecnico di Milano; 2008.

39. De Titta A. Trieste. La sécurité comme effet collatéral [Trieste. Security as a collateral effect]. In: Villes en observation Politiques locales de sécurité urbaine en Italie [Cities under observation Local urban security policies in Italy]. p. 43–74. (Collection « Recherches » du PUCA).

40. Morin P, Leloup X, Girard D, Meilleur R, Gromaire P, Carrier S, et al. L'intervention intersectorielle en santé et services sociaux dans ses liens avec les dimensions de l'habitation et le logement social [Cross-sectoral intervention in health and social services in relation to housing and social housing]. 2015.

41. Colle P. La comunità che fa salute le microaree di Trieste per l'equità [The community that makes health : Trieste's microareas for equity]. Trieste Prima [Internet]. 2018 Jun 15; Available from: https://www.triesteprima.it/partner/asuits/la-comunita-che-fa-salute-le-microaree-di-trieste-per-l-equita.html

42. Wilkinson R, Marmot M. Social Determinants of Health: The Solid Facts. World Health Organization; 2003. 34 p.

43. Braveman P. What are Health Disparities and Health Equity? We Need to Be Clear. Public Health Rep. 2014 Jan;129(1_suppl2):5–8.

44. Wang F, Wang JD. Investing preventive care and economic development in ageing societies: empirical evidences from OECD countries. Health Econ Rev. 2021 Dec;11(1):18.

45. World Health Organization. It's time to build a fairer, healthier world for everyone, everywhere : Health equity and its determinants [Internet]. 2021 [cited 2022 Jul 15]. Available from: https://cdn.who.int/media/docs/default-source/world-health-day-2021/health-equity-and-its-determinants.pdf?sfvrsn=6c36f0a5_1&download=true

46. National Collaborating Centre for Determinants of Health. Let's talk: Health equity [Internet]. Antigonish, NS: National Collaborating Centre for Determinants of Health, St. Francis Xavier University; 2013 [cited 2022 Jul 15]. Available from: https://nccdh.ca/images/uploads/Lets_Talk_Health_Equity_English.pdf
