# Supplementary material for: Integrated Community Care Delivered by Public Health-Care and Social-Care Systems: Results from a Realist Synthesis
Source: Int J Integr Care. 2024 Feb 16;24(1):11. doi: 10.5334/ijic.7042 (PMC10870956; doi:10.5334/ijic.7042)
Supplement: Appendix 2. — Search strategy. [file ijic-24-1-7042-s2.pdf]

Systematic review and Realistic synthesis: Integrated Community care delivered by public health care and social care systems

Search strategy

Health and Social Services Systems, Knowledge Translation and Implementation component of the Quebec SPOR-SUPPORT Unit
Institut universitaire de premiere ligne en sante et services sociaux du CIUSSS de l'Estrie - CHUS

9 février 2021

Nathalie Rheault, M.S.I., Information Specialist
Francis Lacasse, Information Specialist
Hervé Zomahoun, PhD
Delwende-Fatim-Laure Jasmin-Sawadego
Jean-François Allaire
Yacine Thiam, PhD
Paul Morin, PhD

Table des matières

Ovid Medline (2021-02-02) ... 4
Embase (embase.com) (2021-02-03) ... 6
EBSCOhost Cinahl (2021-02-04) ... 9
Ovid PsycINFO (2021-02-04) ... 13
Proquest - Sociological Abstracts (2021-02-04) ... 17
Web of Science Core Collection (2021-02-04) ... 22
Érudit (2021-02) ... 24
Request No 1 : English ... 24
Request No 2 : English -Update ... 25
Request no 3 : French ... 26
Request no 4 : French - Update ... 27
Cairn ... 28
Request in English ... 28
Request in English-Update ... 29
Request in French ... 29
Request in French-Update ... 30
Consulted References ... 31

Search strategy

Research questions:

For the systematic review: *to identify and characterize the integrated community care interventions delivered by public health care and social care systems as well as outcomes related*.

For the realistic synthesis: How, why, for whom, and in what contexts do ICCs deployed by the public health and social care work and produce outcomes?

Population: all or major groups of the population (e.g., seniors, families, residents of social housing complexes) that is home to vulnerable or marginalized populations (material or social disadvantages) within specific local areas
Intervention: Localized community-based health care, Localized community-based social care, or both integrated at the local area level
Comparator: Not applicable
Outcomes: Improved health and well-being of individuals and communities, improved health equity, improved community social capital, social networks, social cohesion, participation in co-production, improved accessibility, availability, and continuity of health care and social care, action on social determinants of health
Setting: Public providers and local area
Study design: Not applicable

Databases

1. Medline
2. Embase
3. CINAHL
4. PsycINFO
5. Sociological Abstracts
6. Web of Science
7. Érudit
8. Cairn

Grey Literature

1. Google Scholar

Ovid Medline (2021-02-02)

Concepts	Search strategy keywords	Number	# Results
Community care - Interventions et soins de proximite (Controlled vocabulary)	Not available	-	-
Community care - Interventions et soins de proximite (Free vocabulary)	(("place based" or "community based" or "area based" or local or proxim* or district* or zone* or territor* or neighbo?rhood) adj3 (approach* or care or healthcare or initiative* or innovation* or intervention* or program* or "health service*" or "social service*" or "healthcare service*" or "care service*")).ti,ab,kw,kf.	#1	38 086
	((outreach* or "out-reach*" or "reaching out") adj3 (approach* or care or healthcare or initiative* or innovation* or intervention* or program* or "health service*" or "social service*" or "healthcare service*" or "care service*")).ti,ab,kw,kf.	#2	4 052
Community care - Interventions et soins de proximite Total	1 or 2	#3	41 817
Deprived area - Territoire défavorisé (Controlled vocabulary)	Vulnerable Populations/ or exp Poverty/ or Unemployment/ or Minority Groups/ or exp disabled persons/ or exp ethnic groups/ or working poor/ or Refugees/ or Healthcare Disparities/ or health status disparities/ or exp Aged/	#4	3 445 946
Deprived area - Territoire défavorisé (Free vocabulary)	("cultural communit*" or depriv* or disabled or disadvantaged or disparit* or elder* or hard-to-reach or inequalit* or inequit* or "low income" or marginal* or poverty or refugee* or senior* or sensitive* or underserved or unemploy* or vulnerable*).ti,ab,kw,kf	#5	1 479 366
	(("at-risk" or poor) adj2 (population* or child* or group* or person* or wom?n or m?n)).ti,ab,kw,kf.	#6	46 767
	((minority or ethnic* or racial) adj2 (group or groups or population*)).ti,ab,kw,kf.	#7	56 062

Concepts	Search strategy keywords	Number	# Results
	((aged or old* or ageing or aging) adj2 (adult* or people or person# or population*)).ti,ab,kw,kf.	#8	210 511
	(social adj2 (disaffiliation or distance or exclusion or isolation or loneliness)).ti,ab,kw,kf	#9	11 866
	5 or 6 or 7 or 8 or 9	#10	1 723 597
Territoire défavorisé	4 or 10	#11	4 660 354
Integrated care (Controlled vocabulary)	exp "Delivery of Health Care, Integrated"/ or exp Comprehensive Health Care/ or intersectoral collaboration/ or Cooperative Behavior/ or interdisciplinary communication/ or Interinstitutional Relations/	#12	372 522
Integrated care (Free vocabulary)	((collabor* or comprehensive* or cooperat* or coordinat* or co-ord* or "cross disciplin*" or "cross sector*" or "cross institution*" or interagenc* or "inter agenc*" or integrat* or interdisciplin* or "inter disciplin*" or interinstitut* or "inter institut*" or "inter organi?ation*" or interorgani?ation* or interprofession* or "inter profession*" or intersector* or "inter sector*" or multidisciplin* or multi or partnership*) adj2 (care or deliver* or health* or management or model* or program* or service* or system?)).ti,ab,kw,kf.	#13	171 409
	(multicare or multiclinic or multiprogram* or multiservice or multicare or "multi team" or multiteam or "shared care").ti,ab,kw,kf.	#14	1 872
	13 or 14	#15	172 894
Integrated care	12 or 15	#16	515 408
Total	3 and 11 and 16	#17	3 111
Filter for date	limit 17 to yr="2003 -Current"	#18	2 486
Update	limit 18 to yr="2019 -Current"	#19	443

Concepts	Search strategy keywords	Number	# Results
This search strategy does not retrieve the following articles: 1) Lopez, P. M., Islam, N., Feinberg, A., Myers, C., Seidl, L., Drackett, E., ... & Wyka, K. (2017). A place-based community health worker program: feasibility and early outcomes, New York City, 2015. *American journal of preventive medicine*, *52*(3), S284-S289.			

Embase (embase.com) (2021-02-03)

Concepts	Search strategy keywords	Number	# Results
Community care - Interventions et soins de proximite (Controlled vocabulary)	Not available	-	-
Community care - Interventions et soins de proximite (Free vocabulary)	(("place based" or "community based" or "area based" or local or proxim* or district* or zone* or territor* or neighbo*rhood) NEAR/3 (approach* or care or healthcare or initiative* or innovation* or intervention* or program* or "health service*" or "social service*" or "healthcare service*" or "care service*")):ti,ab,kw	#1	51 252
	((outreach* or "out-reach*" or "reaching out") NEAR/3 (approach* or care or healthcare or initiative* or innovation* or intervention* or program* or "health service*" or "social service*" or "healthcare service*" or "care service*")):ti,ab,kw	#2	5 978
Community care - Interventions et soins de proximite Total	#1 or #2	#3	56 765
Deprived area - Territoire défavorisé (Controlled vocabulary)	'aged'/de OR 'aged hospital patient'/de OR 'aging'/exp OR 'cultural deprivation'/de OR 'disabled person'/exp OR 'disparities'/de OR 'disparity'/de OR 'ethnic group'/exp OR 'frail elderly'/de OR 'inequalities'/de OR 'inequalities women'/de OR 'inequality'/de OR 'institutionalized elderly'/de OR 'loneliness'/exp OR 'lowest income group'/exp OR 'medically underserved'/de OR 'minority group'/de OR 'poverty'/de OR 'refugee'/exp OR 'social exclusion'/exp OR 'social isolation'/exp OR 'unemployment'/de OR 'very elderly'/de OR 'vulnerable population'/de OR 'working poor'/de	#4	3 750 411

Concepts	Search strategy keywords	Number	# Results
Deprived area - Territoire défavorisé (Free vocabulary)	("cultural communit*" or depriv* or disabled or disadvantaged or disparit* or elder* or "hard-to-reach" or inequalit* or inequit* or "low income" or marginal* or poverty or refugee* or senior* or sensitive* or underserved or unemploy* or vulnerable*):ti,ab,kw	#5	1 905 667
	(("at-risk" or poor) NEAR/2 (population* or child* or group* or person* or wom*n or man or men)):ti,ab,kw	#6	91 553
	((minority or ethnic* or racial) NEAR/2 (group or groups or population*)):ti,ab,kw	#7	72 156
	((aged or old* or ageing or aging) NEAR/2 (adult* or people or person or persons or population*)):ti,ab,kw	#8	280 919
	(social NEAR/2 (disaffiliation or distance or exclusion or isolation or loneliness)):ti,ab,kw	#9	16 060
	#5 or #6 or #7 or #8 or #9	#10	2 249 458
Territoire défavorisé	#4 or #10	#11	5 286 690
Integrated care (Controlled vocabulary)	'collaboration'/de or 'collaborative care team'/de OR 'cooperation'/exp OR 'coordination'/de OR 'integrated health care system'/de OR 'integration'/de OR 'interprofessional'/de OR 'interprofessional collaboration'/de OR 'intersectoral collaboration'/de OR 'multidisciplinary team'/de OR 'partnership'/de	#12	104 236
Integrated care (Free vocabulary)	((collabor* or comprehensive* or cooperat* or coordinat* or co-ord* or "cross disciplin*" or "cross sector*" or "cross institution*" or interagenc* or "inter agenc*" or integrat* or interdisciplin* or "inter disciplin*" or interinstitut* or "inter institut*" or "inter organi*ation*" or interorgani*ation* or interprofession* or "inter profession*" or intersector* or "inter sector*" or multidisciplin* or multi or partnership*) NEAR/2 (care or deliver* or health* or management or model* or program* or service* or system or systems)):ti,ab,kw	#13	233 214
	(multicare or multiclinic or multiprogram* or multiservice or multicare or "multi team" or multiteam or "shared care"):ti,ab,kw	#14	3 133
	#13 or #14	#15	235 542

Concepts	Search strategy keywords	Number	# Results
Integrated care	#12 or #15	#16	322 284
Total	#3 and #11 and #16	#17	2 431
Filter for date	#3 AND #11 AND #16 AND [2003-2019]/py	#18	2 165
Update	#3 AND #11 AND #16 AND [17-9-2019]/sd NOT [5-2-2021]/sd	#19	390

EBSCOhost Cinahl (2021-02-04)

Concepts	Search strategy keywords	Number	# Results
Community care - Interventions et soins de proximite (Controlled vocabulary)	Not available	-	-
Community care - Interventions et soins de proximite (Free vocabulary)	TI (("place based" or "community based" or "area based" or local or proxim* or district* or zone* or territor* or neighbo#rhood) N2 (approach* or care or healthcare or initiative* or innovation* or intervention* or program* or "health service*" or "social service*" or "healthcare service*" or "care service*"))<br><br>OR AB (("place based" or "community based" or "area based" or local or proxim* or district* or zone* or territor* or neighbo#rhood) N2 (approach* or care or healthcare or initiative* or innovation* or intervention* or program* or "health service*" or "social service*" or "healthcare service*" or "care service*"))<br><br>OR SU (("place based" or "community based" or "area based" or local or proxim* or district* or zone* or territor* or neighbo#rhood) N2 (approach* or care or healthcare or initiative* or innovation* or intervention* or program* or "health service*" or "social service*" or "healthcare service*" or "care service*"))	#1	20 191
	TI ((outreach* or "out-reach*" or "reaching out") N2 (approach* or care or healthcare or initiative* or innovation* or intervention* or program* or "health service*" or "social service*" or "healthcare service*" or "care service*"))<br><br>OR AB ((outreach* or "out-reach*" or "reaching out") N2 (approach* or care or healthcare or initiative* or innovation* or intervention* or program* or "health service*" or "social service*" or "healthcare service*" or "care service*"))<br><br>OR SU ((outreach* or "out-reach*" or "reaching out") N2 (approach* or care or healthcare or initiative* or innovation* or intervention* or program* or "health service*" or "social service*" or "healthcare service*" or "care service*"))	#2	2 546
Community care - Interventions et	S1 or S2	#3	22 540

Concepts	Search strategy keywords	Number	# Results
soins de proximite Total			
Deprived area - Territoire défavorisé (Controlled vocabulary)	(MH "Aged+") OR (MH "Aging+") OR (MH "Cultural Deprivation") OR (MH "Disabled+") OR (MH "Ethnic Groups+") OR (MH "Healthcare Disparities") OR (MH "Health Status Disparities") OR (MH "Loneliness") OR (MH "Medically Underserved") OR (MH "Medically Underserved Area") OR (MH "Minority Groups") OR (MH "Poverty") OR (MH "Psychosocial Deprivation") OR (MH "Refugees") OR (MH "Social Isolation+") OR (MH "Social Isolation (NANDA)") OR (MH "Social Isolation (Saba CCC)") OR (MH "Unemployment") OR (MH "Vulnerability")	#4	1 070 438
Deprived area - Territoire défavorisé (Free vocabulary)	TI ("cultural communit*" or depriv* or disabled or disadvantaged or disparit* or elder* or hard-to-reach or inequalit* or inequit* or "low income" or marginal* or poverty or refugee* or senior* or sensitive* or underserved or unemploy* or vulnerable*)<br><br>OR AB ("cultural communit*" or depriv* or disabled or disadvantaged or disparit* or elder* or hard-to-reach or inequalit* or inequit* or "low income" or marginal* or poverty or refugee* or senior* or sensitive* or underserved or unemploy* or vulnerable*)<br><br>OR SU ("cultural communit*" or depriv* or disabled or disadvantaged or disparit* or elder* or hard-to-reach or inequalit* or inequit* or "low income" or marginal* or poverty or refugee* or senior* or sensitive* or underserved or unemploy* or vulnerable*)	#5	450 662
	TI (("at-risk" or poor) N1 (population* or child* or group* or person* or wom#n or m#n))<br><br>OR AB (("at-risk" or poor) N1 (population* or child* or group* or person* or wom#n or m#n))<br><br>OR SU (("at-risk" or poor) N1 (population* or child* or group* or person* or wom#n or m#n))	#6	68 130
	TI ((minority or ethnic* or racial) N1 (group or groups or population*))<br><br>OR AB ((minority or ethnic* or racial) N1 (group or groups or population*))<br><br>OR SU ((minority or ethnic* or racial) N1 (group or groups or population*))	#7	51 403

Concepts	Search strategy keywords	Number	# Results
	TI ((aged or old* or ageing or aging) N1 (adult* or people or person# or population*))<br><br>OR AB ((aged or old* or ageing or aging) N1 (adult* or people or person# or population*))<br><br>OR SU ((aged or old* or ageing or aging) N1 (adult* or people or person# or population*))	#8	123 523
	TI ((social) N1 (disaffiliation or distance or exclusion or isolation or loneliness))<br><br>OR AB ((social) N1 (disaffiliation or distance or exclusion or isolation or loneliness))<br><br>OR SU ((social) N1 (disaffiliation or distance or exclusion or isolation or loneliness))	#9	12 935
	S5 or S6 or S7 or S8 or S9	#10	638 953
Territoire défavorisé	S4 or S10	#11	1 352 932
Integrated care (Controlled vocabulary)	(MH "Collaboration") OR (MH "Cooperative Behavior") OR (MH "Health Care Delivery, Integrated") OR (MH "Interinstitutional Relations") OR (MH "Interprofessional Relations") OR (MH "Multidisciplinary Care Team") OR (MH "Shared Services, Health Care")	#12	130 207
Integrated care (Free vocabulary)	TI ((collabor* or comprehensive* or cooperat* or coordinat* or co-ord* or "cross disciplin*" or "cross sector*" or "cross institution*" or interagenc* or "inter agenc*" or integrat* or interdisciplin* or "inter disciplin*" or interinstitut* or "inter institut*" or "inter organi#ation*" or interorgani#ation* or interprofession* or "inter profession*" or intersector* or "inter sector*" or multidisciplin* or multi or partnership*) N1 (care or deliver* or health* or management or model* or program* or service* or system#))<br><br>OR AB ((collabor* or comprehensive* or cooperat* or coordinat* or co-ord* or "cross disciplin*" or "cross sector*" or "cross institution*" or interagenc* or "inter agenc*" or integrat* or interdisciplin* or "inter disciplin*" or interinstitut* or "inter institut*" or "inter organi#ation*" or interorgani#ation* or interprofession* or "inter profession*" or intersector* or "inter sector*" or multidisciplin* or multi or partnership*) N1 (care or deliver* or health* or management or model* or program* or service* or system#))<br><br>OR SU ((collabor* or comprehensive* or cooperat* or coordinat* or co-ord* or "cross disciplin*" or "cross sector*" or "cross institution*" or interagenc* or "inter agenc*" or integrat* or interdisciplin* or "inter disciplin*" or interinstitut* or "inter institut*" or "inter organi#ation*" or interorgani#ation* or interprofession* or "inter profession*" or intersector*	#13	126 686

Concepts	Search strategy keywords	Number	# Results
	or "inter sector*" or multidisciplin* or multi or partnership*) N1 (care or deliver* or health* or management or model* or program* or service* or system#))		
	TI (multicare or multiclinic or multiprogram* or multiservice or multicare or "multi team" or multiteam or "shared care")<br><br>OR AB (multicare or multiclinic or multiprogram* or multiservice or multicare or "multi team" or multiteam or "shared care")<br><br>OR SU (multicare or multiclinic or multiprogram* or multiservice or multicare or "multi team" or multiteam or "shared care")	#14	1 084
	S13 or S14	#15	127 430
Integrated care	S12 or S15	#16	193 554
Total	S3 and S11 and S16	#17	1 596
Filter for date	Opérateurs de restriction - Date de publication: 20030101-	#18	1 441
Update	Opérateurs de restriction - Date de publication: 201909-	#19	189

Ovid PsycINFO (2021-02-04)

Concepts	Search strategy keywords	Number	# Results
Community care - Interventions et soins de proximite (Controlled vocabulary)	Not available	-	-
Community care - Interventions et soins de proximite (Free vocabulary)	(("place based" or "community based" or "area based" or local or proxim* or district* or zone* or territor* or neighbo?rhood) adj3 (approach* or care or healthcare or initiative* or innovation* or intervention* or program* or "health service*" or "social service*" or "healthcare service*" or "care service*")).ti.<br><br>or (("place based" or "community based" or "area based" or local or proxim* or district* or zone* or territor* or neighbo?rhood) adj3 (approach* or care or healthcare or initiative* or innovation* or intervention* or program* or "health service*" or "social service*" or "healthcare service*" or "care service*")).ab.<br><br>or (("place based" or "community based" or "area based" or local or proxim* or district* or zone* or territor* or neighbo?rhood) adj3 (approach* or care or healthcare or initiative* or innovation* or intervention* or program* or "health service*" or "social service*" or "healthcare service*" or "care service*")).id.	#1	17 056
	((outreach* or "out-reach*" or "reaching out") adj3 (approach* or care or healthcare or initiative* or innovation* or intervention* or program* or "health service*" or "social service*" or "healthcare service*" or "care service*")).ti.<br><br>or ((outreach* or "out-reach*" or "reaching out") adj3 (approach* or care or healthcare or initiative* or innovation* or intervention* or program* or "health service*" or "social service*" or "healthcare service*" or "care service*")).ab.<br><br>or ((outreach* or "out-reach*" or "reaching out") adj3 (approach* or care or healthcare or initiative* or innovation* or intervention* or program* or "health service*" or "social service*" or "healthcare service*" or "care service*")).id.	#2	2 330
Community care - Interventions et	1 or 2	#3	19 218

Concepts	Search strategy keywords	Number	# Results
soins de proximite Total			
Deprived area - Territoire défavorisé (Controlled vocabulary)	exp aging/ or cross cultural differences/ or at risk populations/ or cultural deprivation/ or exp disabilities/ or disadvantaged/ or health disparities/ or exp homeless/ or lower income level/ or minority groups/ or poverty/ or poverty areas/ or exp "racial and ethnic differences"/ or "race and ethnic discrimination"/ or exp "racial and ethnic groups"/ or refugees/ or exp social deprivation/ or unemployment/	#4	413 653
Deprived area - Territoire défavorisé (Free vocabulary)	("cultural communit*" or depriv* or disabled or disadvantaged or disparit* or elder* or hard-to-reach or inequalit* or inequit* or "low income" or marginal* or poverty or refugee* or senior* or sensitive* or underserved or unemploy* or vulnerable*).ti.<br><br>or ("cultural communit*" or depriv* or disabled or disadvantaged or disparit* or elder* or hard-to-reach or inequalit* or inequit* or "low income" or marginal* or poverty or refugee* or senior* or sensitive* or underserved or unemploy* or vulnerable*).ab.<br><br>or ("cultural communit*" or depriv* or disabled or disadvantaged or disparit* or elder* or hard-to-reach or inequalit* or inequit* or "low income" or marginal* or poverty or refugee* or senior* or sensitive* or underserved or unemploy* or vulnerable*).id.	#5	420 092
	(("at-risk" or poor) adj2 (population* or child* or group* or person* or wom?n or m?n)).ti.<br><br>or (("at-risk" or poor) adj2 (population* or child* or group* or person* or wom?n or m?n)).ab.<br><br>or (("at-risk" or poor) adj2 (population* or child* or group* or person* or wom?n or m?n)).id.	#6	22 309
	((minority or ethnic* or racial) adj2 (group or groups or population*)).ti.<br><br>or ((minority or ethnic* or racial) adj2 (group or groups or population*)).ab.<br><br>or ((minority or ethnic* or racial) adj2 (group or groups or population*)).id.	#7	33 937
	((aged or old* or ageing or aging) adj2 (adult* or people or person# or population*)).ti.<br><br>or ((aged or old* or ageing or aging) adj2 (adult* or people or person# or population*)).ab.<br><br>or ((aged or old* or ageing or aging) adj2 (adult* or people or person# or population*)).id.	#8	107 248

Concepts	Search strategy keywords	Number	# Results
	(social adj2 (disaffiliation or distance or exclusion or isolation or loneliness)).ti.<br><br>or (social adj2 (disaffiliation or distance or exclusion or isolation or loneliness)).ab.<br><br>or (social adj2 (disaffiliation or distance or exclusion or isolation or loneliness)).id.	#9	15 488
	5 or 6 or 7 or 8 or 9	#10	550 835
Territoire défavorisé	4 or 10	#11	793 283
Integrated care (Controlled vocabulary)	collaboration/ or cooperation/ or integrated services/ or interdisciplinary research/ or interdisciplinary treatment approach/	#12	38 430
Integrated care (Free vocabulary)	((collabor* or comprehensive* or cooperat* or coordinat* or co-ord* or "cross disciplin*" or "cross sector*" or "cross institution*" or interagenc* or "inter agenc*" or integrat* or interdisciplin* or "inter disciplin*" or interinstitut* or "inter institut*" or "inter organi?ation*" or interorgani?ation* or interprofession* or "inter profession*" or intersector* or "inter sector*" or multidisciplin* or multi or partnership*) adj2 (care or deliver* or health* or management or model* or program* or service* or system?)).ti.<br><br>or ((collabor* or comprehensive* or cooperat* or coordinat* or co-ord* or "cross disciplin*" or "cross sector*" or "cross institution*" or interagenc* or "inter agenc*" or integrat* or interdisciplin* or "inter disciplin*" or interinstitut* or "inter institut*" or "inter organi?ation*" or interorgani?ation* or interprofession* or "inter profession*" or intersector* or "inter sector*" or multidisciplin* or multi or partnership*) adj2 (care or deliver* or health* or management or model* or program* or service* or system?)).ab.<br><br>or ((collabor* or comprehensive* or cooperat* or coordinat* or co-ord* or "cross disciplin*" or "cross sector*" or "cross institution*" or interagenc* or "inter agenc*" or integrat* or interdisciplin* or "inter disciplin*" or interinstitut* or "inter institut*" or "inter organi?ation*" or interorgani?ation* or interprofession* or "inter profession*" or intersector* or "inter sector*" or multidisciplin* or multi or partnership*) adj2 (care or deliver* or health* or management or model* or program* or service* or system?)).id.	#13	67 897

Concepts	Search strategy keywords	Number	# Results
	(multicare or multiclinic or multiprogram* or multiservice or multicare or "multi team" or multiteam or "shared care").ti.<br><br>or (multicare or multiclinic or multiprogram* or multiservice or multicare or "multi team" or multiteam or "shared care").ab.<br><br>or (multicare or multiclinic or multiprogram* or multiservice or multicare or "multi team" or multiteam or "shared care").id.	#14	643
	13 or 14	#15	68 397
Integrated care	12 or 15	#16	98 577
Total	3 and 11 and 16	#17	710
Filter for date	limit 17 to yr="2003 -Current"	#18	562
Update	limit 18 to yr="2019 -Current"	#19	77

Proquest - Sociological Abstracts (2021-02-04)

Concepts	Search strategy keywords	Number	# Results
Community care - Interventions et soins de proximite (Controlled vocabulary)	Not available	-	-
Community care - Interventions et soins de proximite (Free vocabulary)	ti(("place based" OR "community based" OR "area based" OR "local" OR proxim* OR district* OR zone* OR territor* OR neighbo*rhood) AND (approach* OR care OR healthcare OR initiative* OR innovation* OR intervention* OR program* OR "health service*" OR "social service*" OR "healthcare service*" OR "care service*"))<br><br>OR ab(("place based" OR "community based" OR "area based" OR "local" OR proxim* OR district* OR zone* OR territor* OR neighbo*rhood) AND (approach* OR care OR healthcare OR initiative* OR innovation* OR intervention* OR program* OR "health service*" OR "social service*" OR "healthcare service*" OR "care service*"))<br><br>OR su(("place based" OR "community based" OR "area based" OR "local" OR proxim* OR district* OR zone* OR territor* OR neighbo*rhood) AND (approach* OR care OR healthcare OR initiative* OR innovation* OR intervention* OR program* OR "health service*" OR "social service*" OR "healthcare service*" OR "care service*"))	#1	45 257
	ti((outreach* OR "out-reach*" OR "reaching out") AND (approach* OR care OR healthcare OR initiative* OR innovation* OR intervention* OR program* OR "health service*" OR "social service*" OR "healthcare service*" OR "care service*"))<br><br>OR ab((outreach* OR "out-reach*" OR "reaching out") AND (approach* OR care OR healthcare OR initiative* OR innovation* OR intervention* OR program* OR "health service*" OR "social service*" OR "healthcare service*" OR "care service*"))<br><br>OR su((outreach* OR "out-reach*" OR "reaching out") AND (approach* OR care OR healthcare OR initiative* OR innovation* OR intervention* OR program* OR "health service*" OR "social service*" OR "healthcare service*" OR "care service*"))	#2	1 609

Concepts	Search strategy keywords	Number	# Results
Community care - Interventions et soins de proximite Total	1 or 2	#3	46 494
Deprived area - Territoire défavorisé (Controlled vocabulary)	MAINSUBJECT.EXACT("Aging") OR MAINSUBJECT.EXACT.EXPLODE("Cultural Groups") OR MAINSUBJECT.EXACT.EXPLODE("Deprivation") OR MAINSUBJECT.EXACT("Disadvantaged") OR MAINSUBJECT.EXACT("Elderly") OR MAINSUBJECT.EXACT.EXPLODE("Inequality") OR MAINSUBJECT.EXACT("Loneliness") OR MAINSUBJECT.EXACT.EXPLODE("Low Income Areas") OR MAINSUBJECT.EXACT("Marginality") OR MAINSUBJECT.EXACT("Minority Groups") OR MAINSUBJECT.EXACT.EXPLODE("Poverty") OR MAINSUBJECT.EXACT("Refugees") OR MAINSUBJECT.EXACT("Seniority") OR MAINSUBJECT.EXACT("Social Closure") OR MAINSUBJECT.EXACT("Social Distance") OR MAINSUBJECT.EXACT("Social Isolation") OR MAINSUBJECT.EXACT("Unemployment") OR MAINSUBJECT.EXACT("Vulnerability")	#4	216 926
Deprived area - Territoire défavorisé (Free vocabulary)	ti("cultural communit*" OR depriv* OR disabled OR disadvantaged OR disparit* OR elder* OR hard-to-reach OR inequalit* OR inequit* OR "low income" OR marginal* OR poverty OR refugee* OR senior* OR sensitive* OR underserved OR unemploy* OR vulnerable*)<br><br>OR ab("cultural communit*" OR depriv* OR disabled OR disadvantaged OR disparit* OR elder* OR hard-to-reach OR inequalit* OR inequit* OR "low income" OR marginal* OR poverty OR refugee* OR senior* OR sensitive* OR underserved OR unemploy* OR vulnerable*)<br><br>OR su("cultural communit*" OR depriv* OR disabled OR disadvantaged OR disparit* OR elder* OR hard-to-reach OR inequalit* OR inequit* OR "low income" OR marginal* OR poverty OR refugee* OR senior* OR sensitive* OR underserved OR unemploy* OR vulnerable*	#5	237 779
	ti(("at-risk" OR "poor") AND (population* OR child* OR group* OR person* OR wom*n OR man OR men))<br><br>OR ab(("at-risk" OR "poor") AND (population* OR child* OR group* OR person* OR wom*n OR man OR men))<br><br>OR su(("at-risk" OR "poor") AND (population* OR child* OR group* OR person* OR	#6	26 897

Concepts	Search strategy keywords	Number	# Results
	wom*n OR man OR men))		
	ti((minority OR ethnic* OR racial) AND (group OR groups OR population*))<br><br>OR ab((minority OR ethnic* OR racial) AND (group OR groups OR population*))<br><br>OR su((minority OR ethnic* OR racial) AND (group OR groups OR population*))	#7	131 615
	ti((aged OR old* OR ageing OR aging) AND (adult* OR people OR person OR persons OR population*))<br><br>OR ab((aged OR old* OR ageing OR aging) AND (adult* OR people OR person OR persons OR population*))<br><br>OR su((aged OR old* OR ageing OR aging) AND (adult* OR people OR person OR persons OR population*))	#8	59 151
	ti((social) AND (disaffiliation OR distance OR exclusion OR isolation OR loneliness))<br><br>OR ab((social) AND (disaffiliation OR distance OR exclusion OR isolation OR loneliness))<br><br>OR su((social) AND (disaffiliation OR distance OR exclusion OR isolation OR loneliness))	#9	24 364
	5 or 6 or 7 or 8 or 9	#10	395 510
Territoire défavorisé	4 or 10	#11	450 823
Integrated care (Controlled vocabulary)	MAINSUBJECT.EXACT("Cooperation") OR MAINSUBJECT.EXACT("Coordination") OR MAINSUBJECT.EXACT("Interdisciplinary Approach") OR MAINSUBJECT.EXACT("Interprofessional Approach")	#12	15 014
Integrated care (Free vocabulary)	ti((collabor* OR comprehensive* OR cooperat* OR coordinat* OR co-ord* OR "cross disciplin*" OR "cross sector*" OR "cross institution*" OR interagenc* OR "inter agenc*" OR integrat* OR interdisciplin* OR "inter disciplin*" OR interinstitut* OR "inter institut*" OR "inter organi*ation*" OR interorgani*ation* OR interprofession* OR "inter profession*" OR intersector* OR "inter sector*" OR multidisciplin* OR multi OR partnership*) AND (care OR deliver* OR health* OR management OR model* OR program* OR service* OR system OR systems))	#13	93 359

Concepts	Search strategy keywords	Number	# Results
	OR ab((collabor* OR comprehensive* OR cooperat* OR coordinat* OR co-ord* OR "cross disciplin*" OR "cross sector*" OR "cross institution*" OR interagenc* OR "inter agenc*" OR integrat* OR interdisciplin* OR "inter disciplin*" OR interinstitut* OR "inter institut*" OR "inter organi*ation*" OR interorgani*ation* OR interprofession* OR "inter profession*" OR intersector* OR "inter sector*" OR multidisciplin* OR multi OR partnership*) AND (care OR deliver* OR health* OR management OR model* OR program* OR service* OR system OR systems))<br><br>OR su((collabor* OR comprehensive* OR cooperat* OR coordinat* OR co-ord* OR "cross disciplin*" OR "cross sector*" OR "cross institution*" OR interagenc* OR "inter agenc*" OR integrat* OR interdisciplin* OR "inter disciplin*" OR interinstitut* OR "inter institut*" OR "inter organi*ation*" OR interorgani*ation* OR interprofession* OR "inter profession*" OR intersector* OR "inter sector*" OR multidisciplin* OR multi OR partnership*) AND (care OR deliver* OR health* OR management OR model* OR program* OR service* OR system OR systems))		
	ti(multicare OR multiclinic OR multiprogram* OR multiservice OR multicare OR "multi team" OR multiteam OR "shared care")<br><br>OR ab(multicare OR multiclinic OR multiprogram* OR multiservice OR multicare OR "multi team" OR multiteam OR "shared care")<br><br>OR su(multicare OR multiclinic OR multiprogram* OR multiservice OR multicare OR "multi team" OR multiteam OR "shared care")	#14	94
	13 or 14	#15	93 417
Integrated care	12 or 15	#16	100 471
Total	3 and 11 and 16	#17	4 113
Filter for date	17 AND pd(20030101-20211231)	#18	3 149

Concepts	Search strategy keywords	Number	# Results
Update	17 AND pd(20190918-20211231)	#19	

Web of Science Core Collection (2021-02-04)

Concepts	Search strategy keywords	Number	# Results
Community care - Interventions et soins de proximite (Free vocabulary)	TS=(("place based" or "community based" or "area based" or local or proxim* or district* or zone* or territor* or neighborhood or neighbourhood) NEAR/2 (approach* or care or healthcare or initiative* or innovation* or intervention* or program* or "health service*" or "social service*" or "healthcare service*" or "care service*"))	#1	58 898
	TS=((outreach* or "out-reach*" or "reaching out") NEAR/2 (approach* or care or healthcare or initiative* or innovation* or intervention* or program* or "health service*" or "social service*" or "healthcare service*" or "care service*"))	#2	5 328
Community care - Interventions et soins de proximite Total	#1 or #2	#3	63 925
Deprived area - Territoire défavorisé (Free vocabulary)	TS=("cultural communit*" or depriv* or disabled or disadvantaged or disparit* or elder* or hard-to-reach or inequalit* or inequit* or "low income" or marginal* or poverty or refugee* or senior* or sensitive* or underserved or unemploy* or vulnerable*)	#4	2 510 825
	TS=(("at-risk" or poor) NEAR/1 (population* or child* or group* or person* or woman or women or man or men))	#5	47 896
	TS=((minority or ethnic* or racial) NEAR/1 (group or groups or population*))	#6	67 739
	TS=((aged or old* or ageing or aging) NEAR/1 (adult* or people or person$ or population*))	#7	291 689
	TS=((social) NEAR/1 (disaffiliation or distance or exclusion or isolation or loneliness))	#8	22 311
	#4 or #5 or #6 or #7 or #8	#9	2 816 802
Integrated care (Free vocabulary)	TS=((collabor* or comprehensive* or cooperat* or coordinat* or co-ord* or "cross disciplin*" or "cross sector*" or "cross institution*" or interagenc* or "inter agenc*" or integrat* or interdisciplin* or "inter disciplin*" or interinstitut* or "inter institut*" or "inter	#10	468 124

Concepts	Search strategy keywords	Number	# Results
	organisation*" or "inter organization*" or interorganisation* or interorganization* or interprofession* or "inter profession*" or intersector* or "inter sector*" or multidisciplin* or multi or partnership*) NEAR/1 (care or deliver* or health* or management or model* or program* or service* or system$))		
	TS=(multicare or multiclinic or multiprogram* or multiservice or multicare or "multi team" or multiteam or "shared care")	#11	3 919
	#10 or #11	#12	471 442
Total	#3 and #9 and #12	#13	1 178
Filter for date	#3 and #9 and #12<br>Indexes=SCI-EXPANDED, SSCI, A&HCI, CPCI-S, CPCI-SSH, ESCI Timespan=2003-2021	#14	1 069

Érudit (2021-02)

Given the limited computing power of the Érudit database, we had to run two queries to arrive at a result equivalent to those of the other databases. The results were then merged in Endnote and common duplicates between the two queries were removed.

Request No 1 : English

Date : 2021-02-04

Concepts	Search strategy keywords	Operator	# Results
Community care - Interventions et soins de proximite (Free vocabulary)	(("place based" OU "community based" OU "area based" OU local OU proxim* OU district* OU zone* OU territor* OU neighborhood OU neighbourhood OU outreach* ou "out-reach*" ou "reaching out") ET (approach* OU care OU healthcare OU initiative* OU innovation* OU intervention* OU program* OU "health service*" OU "social service*" OU "healthcare service*" OU "care service*"))<br>Dans…Titre,résumé, mots-clés	ET	80
Deprived area - Territoire défavorisé (Free vocabulary)	"cultural community" OU "cultural communities" OU depriv* OU disabled OU disadvantaged OU disparit* OU elder* OU hard-to-reach OU inequalit* OU inequit* OU "low income" OU marginal* OU poverty OU refugee* OU senior* OU sensitive* OU underserved OU unemploy* OU vulnerable* OU (("at-risk" OU poor) ET (population* OU child* OU group* OU person* OU woman OU women OU man OU men)) OU ((minority OU ethnic* OU racial) ET (group OU groups OU population*)) OU ((aged OU old* OU ageing OU aging) ET (adult* OU people OU person OU persons OU population*)) OU ((social) ET (disaffiliation OU distance OU exclusion OU isolation OU loneliness))<br>Dans…Titre,résumé, mots-clés	ET	
Integrated care (Free vocabulary)	((collabor* OU comprehensive* OU cooperat* OU coordinat* OU co-ord* OU "cross disciplin*" OU "cross sector*" OU "cross institution*" OU interagenc* OU "inter agenc*" OU integrat* OU interdisciplin* OU "inter disciplin*" OU interinstitut* OU "inter institut*" OU "inter organi?ation*" OU interorgani?ation* OU interprofession* OU "inter profession*" OU intersector* OU "inter sector*" OU multidisciplin* OU multi OU partnership*) ET (care OU deliver* OU health* OU management OU model* OU program* OU service* OU system?)) OR multicare OU multiclinic OU multiprogram* OU multiservice OU multicare OU "multi team" OU multiteam OU "shared care"<br>Dans…Titre,résumé, mots-clés	ET	

Concepts	Search strategy keywords	Operator	# Results
Year or range of years	De 2003 à 2021		

Request No 2 : English -Update

Date : 2021-02-05

Concepts	Search strategy keywords	Operator	# Results
Community care - Interventions et soins de proximite (Free vocabulary)	(("place based" OU "community based" OU "area based" OU local OU proxim* OU district* OU zone* OU territor* OU neighborhood OU neighbourhood OU outreach* ou "out-reach*" ou "reaching out") ET (approach* OU care OU healthcare OU initiative* OU innovation* OU intervention* OU program* OU "health service*" OU "social service*" OU "healthcare service*" OU "care service*"))<br>Dans…Titre,résumé, mots-clés	ET	14
Deprived area - Territoire défavorisé (Free vocabulary)	"cultural community" OU "cultural communities" OU depriv* OU disabled OU disadvantaged OU disparit* OU elder* OU hard-to-reach OU inequalit* OU inequit* OU "low income" OU marginal* OU poverty OU refugee* OU senior* OU sensitive* OU underserved OU unemploy* OU vulnerable* OU (("at-risk" OU poor) ET (population* OU child* OU group* OU person* OU woman OU women OU man OU men)) OU ((minority OU ethnic* OU racial) ET (group OU groups OU population*)) OU ((aged OU old* OU ageing OU aging) ET (adult* OU people OU person OU persons OU population*)) OU ((social) ET (disaffiliation OU distance OU exclusion OU isolation OU loneliness))<br>Dans…Titre,résumé, mots-clés	ET	
Integrated care (Free vocabulary)	((collabor* OU comprehensive* OU cooperat* OU coordinat* OU co-ord* OU "cross disciplin*" OU "cross sector*" OU "cross institution*" OU interagenc* OU "inter agenc*" OU integrat* OU interdisciplin* OU "inter disciplin*" OU interinstitut* OU "inter institut*" OU "inter organi?ation*" OU interorgani?ation* OU interprofession* OU "inter profession*" OU intersector* OU "inter sector*" OU multidisciplin* OU multi OU partnership*) ET (care OU deliver* OU health* OU management OU model* OU program* OU service* OU system?)) OR multicare OU multiclinic OU multiprogram* OU multiservice OU multicare OU "multi team" OU multiteam OU "shared care"<br>Dans…Titre,résumé, mots-clés	ET	

Concepts	Search strategy keywords	Operator	# Results
Year or range of years	De 2019 à 2021		

Request no 3 : French

Date : 2021-02-05

Concepts	Search strategy keywords	Operator	# Results
Community care - Interventions et soins de proximite (Free vocabulary)	(communaut* OU district* OU faubourg* OU local* OU proacti* OU proxim* OU region* OU quartier* OU territor* OU voisin* OU zone*) ET (approch* OU demarche* OU initiative* OU innovation* OU intervention* OU lutte* OU médecine* OU "prise en charge" OU program* OU santé OU service* OU soin*)<br>Dans…Titre,résumé, mots-clés	AND	173
Deprived area - Territoire défavorisé (Free vocabulary)	aine* OU appauvri* OU chomage* OU chomeu* OU "communaute culturelle" OU "communautes culturelles" OU defavor* OU demuni* OU denuement OU desavantag* OU "difficile à atteindre" OU "difficile à joindre" OU disparit* OU "faible revenu" OU "faibles revenus" OU handicap* OU inegalit* OU iniquit* OU invalide* OU "mal desservi" OU "mal desservis" OU marginal* OU misere* OU pauvret* OU precarite* OU refugie* OU vulnerab* OU ((risque OU pauvre*) ET (population* OU enfant* OU groupe* OU personne* OU femme* OU homme*)) OU ((minoritaire* OU ethnique* OU racial*) ET (groupe* OU population*)) OU ((age OU ages OU vieillisant*) ET (adulte* Ou personne* OU population*)) OU ((social) ET (desaffiliation* OU distance* OU exclusion* Ou isolation* OU isolement* OU marginalisation*))<br>Dans…Titre,résumé, mots-clés	AND	
Integrated care (Free vocabulary)	((collabor* OU complet* OU cooperat* OU coordonn* OU exhaustif* OU global OU integre* OU interdisciplina* OU interinstitution* OU interorganisation* OU interprofessionn* OU intersectoriel* OU inter-sectoriel* OU multidisciplina* OU partenariat* OU pluridisciplina* OU transdisciplina*) ET (administration OU distribu* OU gestion* OU management OU modele* OU program* OU sante OU service* OU soin* OU système?)) OU multisoin* OU multiclinique* OU policlinique* OU multiprogramme* OU multiservice OU "multi-service" OU multiservices OU "multi-services"<br>Dans…Titre,résumé, mots-clés	AND	

Concepts	Search strategy keywords	Operator	# Results
Year or range of years	De 2003 à 2021		

Request no 4 : French - Update

Date : 2021-02-05

Concepts	Search strategy keywords	Operator	# Results
Community care - Interventions et soins de proximite (Free vocabulary)	(communaut* OU district* OU faubourg* OU local* OU proacti* OU proxim* OU region* OU quartier* OU territor* OU voisin* OU zone*) ET (approch* OU demarche* OU initiative* OU innovation* OU intervention* OU lutte* OU médecine* OU "prise en charge" OU program* OU santé OU service* OU soin*)<br>Dans…Titre,résumé, mots-clés	AND	15
Deprived area - Territoire défavorisé (Free vocabulary)	aine* OU appauvri* OU chomage* OU chomeu* OU "communaute culturelle" OU "communautes culturelles" OU defavor* OU demuni* OU denuement OU desavantag* OU "difficile à atteindre" OU "difficile à joindre" OU disparit* OU "faible revenu" OU "faibles revenus" OU handicap* OU inegalit* OU iniquit* OU invalide* OU "mal desservi" OU "mal desservis" OU marginal* OU misere* OU pauvret* OU precarite* OU refugie* OU vulnerab* OU ((risque OU pauvre*) ET (population* OU enfant* OU groupe* OU personne* OU femme* OU homme*)) OU ((minoritaire* OU ethnique* OU racial*) ET (groupe* OU population*)) OU ((age OU ages OU vieillisant*) ET (adulte* Ou personne* OU population*)) OU ((social) ET (desaffiliation* OU distance* OU exclusion* Ou isolation* OU isolement* OU marginalisation*))<br>Dans…Titre,résumé, mots-clés	AND	
Integrated care (Free vocabulary)	((collabor* OU complet* OU cooperat* OU coordonn* OU exhaustif* OU global OU integre* OU interdisciplina* OU interinstitution* OU interorganisation* OU interprofessionn* OU intersectoriel* OU inter-sectoriel* OU multidisciplina* OU partenariat* OU pluridisciplina* OU transdisciplina*) ET (administration OU distribu* OU gestion* OU management OU modele* OU program* OU sante OU service* OU soin* OU système?)) OU multisoin* OU multiclinique* OU policlinique* OU multiprogramme* OU multiservice OU "multi-service" OU multiservices OU "multi-services"<br>Dans…Titre,résumé, mots-clés	AND	
Year or range of years	De 2019 à 2021		

Cairn

Due to the limited computing power of the Cairn database, we had to run four queries and limit ourselves to the use of one concept (Interventions and Community Care). The results were then merged in Endnote and common duplicates between the four queries were removed.

Request in English

Date : 2021-02-09

Concepts	Search strategy keywords	Operator	# Results
Community care - Interventions et soins de proximite (Free vocabulary)	Dans Titre d'article/chapitre : ("place based" OU "community based" OU "area based" OU local OU proxim* OU district* OU zone* OU territor* OU neighborhood OU neighbourhood OU outreach* OU "out-reach*" OU "reaching out") W/2 (approach* OU care OU healthcare OU initiative* OU innovation* OU intervention* OU program* OU "health service*" OU "social service*" OU "healthcare service*" OU "care service*")	ET	55
Filtre de date	Dans Année de parution : Entre 2003 et 2021		

Date : 2021-02-09

Concepts	Search strategy keywords	Operator	# Results
Community care - Interventions et soins de proximite (Free vocabulary)	Dans Résumé: ("place based" OU "community based" OU "area based" OU local OU proxim* OU district* OU zone* OU territor* OU neighborhood OU neighbourhood OU outreach* OU "out-reach*" OU "reaching out") W/2 (approach* OU care OU healthcare OU initiative* OU innovation* OU intervention* OU program* OU "health service*" OU "social service*" OU "healthcare service*" OU "care service*")	ET	178
Filtre de date	Dans Année de parution : Entre 2003 et 2019		

Request in English-Update

Date : 2021-02-09

Concepts	Search strategy keywords	Operator	# Results
Community care - Interventions et soins de proximite (Free vocabulary)	Dans Titre d'article/chapitre : ("place based" OU "community based" OU "area based" OU local OU proxim* OU district* OU zone* OU territor* OU neighborhood OU neighbourhood OU outreach* OU "out-reach*" OU "reaching out") W/2 (approach* OU care OU healthcare OU initiative* OU innovation* OU intervention* OU program* OU "health service*" OU "social service*" OU "healthcare service*" OU "care service*")	ET	11
Filtre de date	Dans Année de parution : Entre 2019 et 2021		

Date : 2021-02-09

Concepts	Search strategy keywords	Operator	# Results
Community care - Interventions et soins de proximite (Free vocabulary)	Dans Résumé: ("place based" OU "community based" OU "area based" OU local OU proxim* OU district* OU zone* OU territor* OU neighborhood OU neighbourhood OU outreach* OU "out-reach*" OU "reaching out") W/2 (approach* OU care OU healthcare OU initiative* OU innovation* OU intervention* OU program* OU "health service*" OU "social service*" OU "healthcare service*" OU "care service*")	ET	23
Filtre de date	Dans Année de parution : Entre 2019 et 2021		

Request in French

Date : 2021-02-09

Concepts	Search strategy keywords	Operator	# Results
Community care - Interventions et soins de proximite (Free vocabulary)	Dans Titre d'article/chapitre : (communaut* OU district* OU faubourg* OU local* OU proacti* OU proxim* OU region* OU quartier* OU territor* OU voisin* OU zone*) W/2 (approch* OU demarche* OU initiative* OU innovation* OU intervention* OU lutte* OU médecine* OU "prise en charge" OU program* OU santé OU service* OU soin*)	ET	264
Filtre de date	Dans Année de parution : Entre 2003 et 2019		

Date : 2021-02-09

Concepts	Search strategy keywords	Operator	# Results
Community care - Interventions et soins de proximite (Free vocabulary)	Dans Résumé: (communaut* OU district* OU faubourg* OU local* OU proacti* OU proxim* OU region* OU quartier* OU territor* OU voisin* OU zone*) W/2 (approch* OU demarche* OU initiative* OU innovation* OU intervention* OU lutte* OU médecine* OU "prise en charge" OU program* OU santé OU service* OU soin*)	ET	666
Filtre de date	Dans Année de parution : Entre 2003 et 2019		

Request in French-Update

Date : 2021-02-09

Concepts	Search strategy keywords	Operator	# Results
Community care - Interventions et soins de proximite (Free vocabulary)	Dans Titre d'article/chapitre : (communaut* OU district* OU faubourg* OU local* OU proacti* OU proxim* OU region* OU quartier* OU territor* OU voisin* OU zone*) W/2 (approch* OU demarche* OU initiative* OU innovation* OU intervention* OU lutte* OU médecine* OU "prise en charge" OU program* OU santé OU service* OU soin*)	ET	42
Filtre de date	Dans Année de parution : Entre 2019 et 2021		

Date : 2021-02-09

Concepts	Search strategy keywords	Operator	# Results
Community care - Interventions et soins de proximite (Free vocabulary)	Dans Résumé: (communaut* OU district* OU faubourg* OU local* OU proacti* OU proxim* OU region* OU quartier* OU territor* OU voisin* OU zone*) W/2 (approch* OU demarche* OU initiative* OU innovation* OU intervention* OU lutte* OU médecine* OU "prise en charge" OU program* OU santé OU service* OU soin*)	ET	78
Filtre de date	Dans Année de parution : Entre 2019 et 2021		

Consulted References

Baker PRA, Francis DP, Soares J, Weightman AL, Foster C. Community wide interventions for increasing physical activity. Cochrane Database of Systematic Reviews 2015, Issue 1. Art. No.: CD008366. DOI: 10.1002/14651858.CD008366.pub3.

Balogh R, McMorris CA, Lunsky Y, Ouellette-Kuntz H, Bourne L, Colantonio A, Gonçalves-Bradley DC. Organising healthcare services for persons with an intellectual disability. Cochrane Database of Systematic Reviews 2016, Issue 4. Art. No.: CD007492. DOI: 10.1002/14651858.CD007492.pub2.

Jia, L., Yuan, B., Huang, F., Lu, Y., Garner, P., & Meng, Q. (2014). Strategies for expanding health insurance coverage in vulnerable populations. Cochrane Database of Systematic Reviews, (11).

Kruis AL, Smidt N, Assendelft WJJ, Gussekloo J, Boland MRS, Rutten-van Mölken M, Chavannes NH. Integrated disease management interventions for patients with chronic obstructive pulmonary disease. Cochrane Database of Systematic Reviews 2013, Issue 10. Art. No.: CD009437. DOI: 10.1002/14651858.CD009437.pub2.

Nieuwboer, M. S., van der Sande, R., van der Marck, M. A., Olde Rikkert, M. G., & Perry, M. (2019). Clinical leadership and integrated primary care: A systematic literature review. European Journal of General Practice, 25(1), 7-18.

Rutebemberwa E, Kinengyere AA, Ssengooba F, Pariyo GW, Kiwanuka SN. Financial interventions and movement restrictions for managing the movement of health workers between public and private organizations in low- and middle-income countries. Cochrane Database of Systematic Reviews 2014, Issue 2. Art. No.: CD009845. DOI: 10.1002/14651858.CD009845.pub2.

Sadler, E., Potterton, V., Anderson, R., Khadjesari, Z., Sheehan, K., Butt, F., ... & Sandall, J. (2019). Service user, carer and provider perspectives on integrated care for older people with frailty, and factors perceived to facilitate and hinder implementation: A systematic review and narrative synthesis. PloS one, 14(5), e0216488.

Vold, J. H., Aas, C., Leiva, R. A., Vickerman, P., Chalabianloo, F., Løberg, E. M., ... & Fadnes, L. T. (2019). Integrated care of severe infectious diseases to people with substance use disorders; a systematic review. BMC infectious diseases, 19(1), 306.

Zonneveld, N., Driessen, N., Stüssgen, R. A. J., Minkman, M. N., (2018). Values of integrated care : A Systematic Review. International Journal of Integrated Care, 18(4), 1-12.
