# Supplementary material for: Integrated Community Care Delivered by Public Health-Care and Social-Care Systems: Results from a Realist Synthesis
Source: Int J Integr Care. 2024 Feb 16;24(1):11. doi: 10.5334/ijic.7042 (PMC10870956; doi:10.5334/ijic.7042)
Supplement: Appendix 3. — Data Coding Guide. [file ijic-24-1-7042-s3.pdf]

Appendix 3. Coding grid

Data Coding Guide

(First version: December 2020 - Updated: May 2021)

The design of this data coding guide is essentially inspired by the research question, its PICOs (Population/Intervention/Comparison/Outcome/setting) of the systematic review and the initial rough theories constituted in a form of CMO (Context-Mechanism-Effect) configurations for the realistic synthesis.

As more than two-thirds (2/3) of the writings are published in English, the data will be extracted directly in this language in order to reduce discordances related to the translation or interpretation of texts.

Given the complexity of community-based interventions in health and social care, the empirical data extracted are structured around criteria analysis divided as characteristics groups. These characteristics are declined in variables (or components) that are subject to be mutually inclusive and exhaustive modalities, each category taking into account with all the possible answers. Within a specific section, several modes associated with the same component may be selected simultaneously, in line with the multifaceted complexity of ICC.

The first section of the guide characterises the typology of the different publications (scientific studies and grey literature) used in the systematic review on ICC (community-based interventions deployed by the public health care and social care network, alone or in partnership). The sections on the intervention, its operation, contexts, mechanisms and outcomes specify the different components of the evidence extracted from the selected publications.

A data extraction grid has been designed to support the extraction process, particularly of contextual data (1st part of the codification guide, for the systematic review). The empirical statements of the CMO configurations are directly recorded in an additional tool to simplify the analysis of causal relations related to the evidence extracted from selected publications and considered as evidence (2nd part of the codification guide, for the realistic synthesis).

Within a realistic logic (realistic synthesis), the mechanism is at the heart of the intervention process. The mechanism is real, but hidden. It is underlying (happens in the heads of the actors) and therefore not directly observable. Indeed, the relevant mechanisms cannot be identified without reference to the outcomes just as important aspects of the context cannot be identified without reference to the mechanisms. Therefore, data related to mechanism will be at the heart of the extraction process and will ultimately provide elements to answer the research question: how, why, for whom, and in what contexts do ICCs deployed by the public health and social care work and produce outcomes?

The definition of the preliminary code structure is based on a deductive approach that integrates concepts already well known in the literature associated with integrated community care (ICC).

Appendix 3. Coding grid

PART 1 - CONTEXTUALS DATAS

**VARIABLE OF INTEREST** (or component)	**NAME OF VARIABLES**	**DESCRIPTION**	**MODES**	**COMMENTS** (explanation or clarification)
**Section Characteristics of the publication**				
**no_publication**	Code number	Code number associated with the publication included.	**enter the code number associated with the included publication**	
**author**	First author	Name of the main author.	**describe as indicated in the publication**	
**year**	Year	Year of publication.	**describe as indicated in the publication**	
**title**	Title	Title of the publication.	**describe as indicated in the publication**	
**language**	Publication language	Specify the language of publication of the full article.	**English**<br>**French**<br>**Italian**<br>**Other** (specify)	
**extractors**	Datas extractors	Write the initials of the people who do the data extraction and coding.	Enter the initials of data extractors	
**country**	Country	Country where the study was carried out.	**Australia**<br>**Canada**<br>**Spain**<br>**United states of America**<br>**France**<br>**Italy**<br>**Japan**<br>**England**<br>**Scotland**<br>**Transnational**<br>**Other** (specify)	Write **nd** if not declared.
**period**	Time period	Describe the period to which the publication refer.	**health crisis**<br>**systemic reform**<br>**change of political regime**<br>**other** (specify)	Write **ns** if not specified.
**source**	Source	Specify the source of selection of the retained publication.	**databases**<br>**snowball method**<br>**other** (specify)	
**publication**	Type of publication included	Describe the type of publication included.	**scientific paper**<br>**scientific report**	**Source** : Quatrième Conférence internationale sur la

Appendix 3. Coding grid

VARIABLE OF INTEREST (or component)	NAME OF VARIABLES	DESCRIPTION	MODES	COMMENTS (explanation or clarification)
		- Scientific article: article published in a peer-reviewed scientific journal or magazine.<br>- Grey Literature: What is produced by all levels of government, public education and research, commerce and industry, in paper or digital format, and which is not controlled by commercial publishing	**other report**<br>**conference abstract**<br>**thesis or memoir**<br>**other grey literature**	littérature grise (Fourth International Conference on Grey Literature), Washington, DC, octobre 1999
**qualitative_study**	Type of qualitative study	Category of the qualitative scientific study included.	**description study**<br>**case study**<br>**exploratory study**<br>**framework study**<br>**policy or guideline document**<br>**qualitative evaluation**<br>**research protocol**<br>**other** (specify)	**Source** : Hong et coll. (2018) Mixed Methods Appraisal Tool et Royer (2016) Peut-on fixer une typologie des méthodes qualitatives?<br><br>Write na if not applicable.
**mixed_study**	Type of mixed study	Categories of mixed scientific study included.	**descriptive study**<br>**case study**<br>**research and evaluation protocol**<br>**evaluative research**<br>**other** (specify)	**Source** : Hong et coll. (2018) Mixed Methods Appraisal Tool<br><br>Write na if not applicable.
**design**	Type of design	Describe the type of design of **scientific studies only**.	**before-and-after (BA) studies**<br>**cross-sectional study**<br>**focus group study**<br>**interview study**<br>**longitudinal studies**<br>**meta-analysis**<br>**non-participant observation**<br>**participant observation**<br>**pre–post study**<br>**survey**<br>**systematic review**<br>**not scienific publication**<br>**other (specify)**	**Source** : Methods for the development of NICE public health guidance (third edition): Process and methods - Appendix D Glossary of study designs https://www.nice.org.uk/process/pmg4/chapter/appendix-d-glossary-of-study-designs
**grey_litterature**	Type of grey	Categories of grey	**ministries or public entities**	Write **na** if not

Appendix 3. Coding grid

VARIABLE OF INTEREST (or component)	NAME OF VARIABLES	DESCRIPTION	MODES	COMMENTS (explanation or clarification)
	literature	literature included.	(reference framework, policy or orientation document, public report, action plan, etc.).<br>**academic** (evaluation report, support guide, scientific opinion, etc.).<br>**private sector** (private foundations, professional associations, etc.)<br>**community-based organisations**<br>**civil society** (civil and citizen organisations)<br>**other** (specify)	applicable.
**approach**	Intervention approach	Describe the privileged intervention approach.	**localized community health care**<br>**localized community social care**<br>**integrated community- care**<br>**other** (specify)	
**focus**	Local intervention focus (axis)	Describe the preferred area of intervention.	**implementing** an intervention<br>**intervening** in proximity<br>**managing** an intervention<br>**other** (specify)	
**Section Characteristics of the actors of the intervention** (BY WHOM: ACTORS)				
**initiators**	Intervention initiators	Describe the public and private sector, community and civil society actors instigating the intervention.	**world** organization<br>**transnational** corporation<br>government or public organisation of **nation - state - region**<br>**municipal - local** government or public entity<br>ministry or public entity of **healthcare**<br>ministry or public entity of **social care**<br>ministry or public entity of **employment - poverty - social solidarity**<br>ministry or public entity of **education**<br>ministry or public entity of **public security**<br>public entity of **social housing**<br>**academic and research** institutions<br>**NGOs** and **community-based** organisations (non-profit cooperatives. ...)<br>**civic entities** (neighbourhood-	Write **nd** if not declared.

Appendix 3. Coding grid

**VARIABLE OF INTEREST** (or component)	**NAME OF VARIABLES**	**DESCRIPTION**	**MODES**	**COMMENTS** (explanation or clarification)
			senior-user's associations)<br>**social economy** organisations (profit cooperatives, …)<br>**private** corporations (family physician group, private clinics, **…)**<br>**other** (specify)	
**promotors**	Promoters of intervention	Describe public and private sector, community and civil society actors (health care and social services providers, care managers, elected representatives, others technicians and professionals) leading the intervention.	**world** organization<br>**transnational** corporation<br>government or public organisation of **nation - state - region**<br>**municipal - local** government or public entity<br>ministry or public entity of **healthcare**<br>ministry or public entity of **social care**<br>ministry or public entity of **employment - poverty - social solidarity**<br>ministry or public entity of **education**<br>ministry or public entity of **public security**<br>public entity of **social housing**<br>**academic and research** institutions<br>**NGOs** and **community-based** organisations (non-profit cooperatives. ...)<br>**civic entities** (neighbourhood-senior-user's associations)<br>**social economy** organisations (profit cooperatives, …)<br>**private** corporations (family physician group, private clinics, **…)**<br>**other** (specify)	Write **nd** if not declared.
**financers**	Financers of intervention	Describe funding sources of the intervention.	**world** organization<br>**transnational** corporation<br>government or public organisation of **nation - state - region**<br>**municipal - local** government or public entity<br>ministry or public entity of **healthcare**<br>ministry or public entity of **social care**	Write **nd** if not declared.

Appendix 3. Coding grid

VARIABLE OF INTEREST (or component)	NAME OF VARIABLES	DESCRIPTION	MODES	COMMENTS (explanation or clarification)
			ministry or public entity of **employment - poverty - social solidarity**<br>ministry or public entity of **education**<br>ministry or public entity of **public security**<br>public entity of **social housing**<br>**academic and research** institutions<br>**NGOs** and **community-based** organisations (non-profit cooperatives. ...)<br>**civic entities** (neighbourhood- senior-user's associations)<br>**social economy** organisations (profit cooperatives, …)<br>**private** corporations (family physician group, private clinics, **…)**<br>**other** (specify)	
**cross_sectorals_ partners**	Cross-sectoral partners of intervention	Describe the partners of the intervention: public and private sector, community and civil society groups engaged in cross-sectoral action to address health and its social determinants.	**world** organization<br>**transnational** corporation<br>government or public organisation of **nation - state - region**<br>**municipal - local** government or public entity<br>ministry or public entity of **healthcare**<br>ministry or public entity of **social care**<br>ministry or public entity of **employment - poverty - social solidarity**<br>ministry or public entity of **education**<br>ministry or public entity of **public security**<br>public entity of **social housing**<br>**academic and research** institutions<br>**NGOs** and **community-based** organisations (non-profit cooperatives. ...)<br>**civic entities** (neighbourhood- senior-user's associations)<br>**social economy** organisations (profit cooperatives, …)	Write **nd** if not declared.

Appendix 3. Coding grid

VARIABLE OF INTEREST (or component)	NAME OF VARIABLES	DESCRIPTION	MODES	COMMENTS (explanation or clarification)
			**private** corporations (family physician group, private clinics, **…)**<br>**other** (specify)	
**collaborators**	Collaborators (people) working as providers, managers or researchers within the health and social care network	Describe the various professionals, managers and researchers from the public network, the other sector who work collaboratively in the intervention.	**general practitioners** (family physicians and paediatricians)<br>**medical specialists**<br>**neighbourhood pharmacists**<br>**paramedics**<br>**nurses**<br>**health therapeutic services** (physiotherapists, psychotherapists, ergotherapists, ...)<br>**community animators/organizers**<br>**psychosocial workers** (mental health, youth, elders, social workers, …)<br>**outreach workers**<br>**development workers**<br>**researchers/students-researchers** (research and academic institutions)<br>**care team managers** (health and social care)<br>**administrative and technical manager**<br>**other** (specify)	Write **nd** if not declared.
**Section Characteristics of the target population** (FOR WHOM: BACKGROUND)				
**for_whom**	Target population	All or important groups of the population of the targeted intervention area	**all people** of the community<br>**economically deprived people** (low income, material poverty, …)<br>**social vulnerable people** (single, divorced or widowed)<br>**people with major health problems and long-term illnesses** (chronically ill or bedridden people)<br>**people with a functional limitation** (disability or loss of autonomy)<br>**people with a mental health problem**<br>**elderly population** (with social and economic deprivation, with or no illnesses and disabilities, with complex needs)<br>**people not reached by the traditional health and social care**	Write **nd** if not declared.

Appendix 3. Coding grid

VARIABLE OF INTEREST (or component)	NAME OF VARIABLES	DESCRIPTION	MODES	COMMENTS (explanation or clarification)
			**services and with chronic and complex conditions** (marginalized, homebound patients, homeless, roofless, without permanent residence, without status, precarious immigration status, illegal drug users, sex trade workers, ...)<br>**cultural or ethnic group** (immigrant, indigenous, refugee, asylum seeker, racial/ethnic minority, ...)<br>**families living in vulnerable situations with multiple and complex needs** (children under 17 years old, mother under 20 years old, one parent with a dependency, mental health or intellectual disability problem, abuse/neglect, vulnerable single parents, residents of social housing, ...)<br>**other** (specify)	
**Section Characteristics of the intervention area** (WHERE - BACKGROUND)				
**by_whom**	Local area	Describe the intervention area in terms of the services availability , particularly public services.	**underserved communities** (medically and social care disadvantaged communities)<br>**rural or remote areas** (distant and vast, small community, isolated, ...)<br>**urban and multi-problematic local area** (deprived neighborhood, urban metropolitan area, ...)<br>**densely populated**<br>**unpopulated**<br>**available public health and social care services** (hospitals, health centres, ...)<br>**availability of other health and social services** (pharmacies, private clinics, ...)<br>**availability of community resources** (health and social care, social housing, place meeting, ...)<br>**other availability public services** (post office, police, school, library, transport, park, path, ...)	Write "yes" if there are many, "no" if there are none and "a little" if there are not enough.<br><br>Write **nd** if not declared.

Appendix 3. Coding grid

VARIABLE OF INTEREST (or component)	NAME OF VARIABLES	DESCRIPTION	MODES	COMMENTS (explanation or clarification)
			**economic services available in the area** (restaurants, bars, banks, shops, supermarket, ...)<br>**place where people reside and live on a daily basis**<br>**geographically perceived identity**<br>**heritage**<br>**other** (specify)	
**Section Characteristics of the context of the intervention** (IN WHAT CONTEXT)				
**general_context**	General context of intervention	Describe the external factors that may influence the intervention (barriers and facilitators).	**institutional barriers** (institutional framework)<br>**institutional facilitators** (institutional framework)<br>**political barriers** (political support)<br>**political facilitators** (political support)<br>**economic barriers** (economic constraints)<br>**economic facilitators**<br>**environmental barriers** (natural barriers, ecosystems)<br>**environmental facilitators** (natural barriers, ecosystems)<br>**social barriers** (cohesion and interpersonal relations)<br>**social facilitators** (cohesion and interpersonal relations)<br>**cultural barriers** (cultural influence)<br>**cultural facilitators** (cultural influence)<br>**demographic barriers** (structure and dynamics)<br>**demographic facilitators** (structure and dynamics)<br>**other** (specify)	Write **nd** if not declared.
**challenge_care_acces_availibility**	Problematic situations of access and availability	Describe the problematic situations related to the access and availability of care and services on which the intervention wants to act in order to meet the needs of the population (need to be	**few service points available** (lack of service providers, ...)<br>**limited number** of social, training and recreational spaces<br>**not enough spaces** for professional help support and collective reflections<br>**lack of support** from local elected representatives (financial, materials,	Write **nd** if not declared.

Appendix 3. Coding grid

VARIABLE OF INTEREST (or component)	NAME OF VARIABLES	DESCRIPTION	MODES	COMMENTS (explanation or clarification)
		met or fulfilled, problem to be solved and situation to be improved).	…)<br>**lack of knowledge or understanding** of strategic and governance managers on services in proximity to populations<br>**problematic gateways** (too diverse, difficult to access, complex to understand, substantial structural barriers**, …**)<br>**challenge of renewing professionals** (non-favorable working conditions for professionals)<br>**lack of resources for outreach services** (outreach health and social care workers)<br>**inadequate and insecure care environment for vulnerable people with chronic and complex needs and a criminal record** (co-location of services common: ex.: public health clinics sharing a same building with police, one building housing for many different agencies and services)<br>**healthcare and social care geographically distant from the people who are difficult to reach by the health system** (unattached patients, marginalized, with complex or highly specialized needs, living on the street or homeless, illegal immigrants, paperless, drug addicts, administrative hassles, language barriers, financial issues, …)<br>**failure to take care of patients in vulnerable health situations** (elderly and disabled persona, chronically ill, bedridden people, ...)<br>**unanswered** health care and social care needs<br>**financing challenge**<br>**other** (specify)	
**challenge_care_continuity_quality**	Problematic situations of continuity and	Describe the problematic situations related to the	**access to episodic and low-quality care** (quality)<br>**access to discontinuous healthcare**	Write **nd** if not declared.

Appendix 3. Coding grid

**VARIABLE OF INTEREST** (or component)	**NAME OF VARIABLES**	**DESCRIPTION**	**MODES**	**COMMENTS** (explanation or clarification)
	quality	continuity and quality of health and social care services on which the intervention wants to act in order to meet the needs of the population (need to be met or filled, problem to be solved and situation to be improved).	**and social care trajectories** (continuity : financial and operational challenges)<br>**access to fragmented healthcare and social care delivered** (uncoordinated care between primary care on a same area : integration multiple professionals and partners, hospitals, emergency rooms, pharmacies and other sources of care)<br>**lack of community-based intervention**<br>**unflexible intervention practices** (not adapted to the realities and needs)<br>**limits of mono-disciplinary practices** (complex needs)<br>**limited competences to act on complex realities** (individuals and groups)<br>**conciliate** the complex needs of patients with the requirements of professionals and health authorities<br>**unclear knowledge of principles and strategies** of proximity intervention<br>**unflexible management practices/accountability poorly adapted** to the specificity of proximity intervention<br>**patient engagement challenge**<br>**other** (specify)	
**issues**	Intervention issues (strengths, weaknesses or not applicable)	Describe the issues related to the implementation, practice and management of community-based intervention.	**understanding of the territory of intervention** (geographic, lived, perceived and conceived)<br>**understanding lifestyles and needs of the population** (residents' needs, professional expectations, social capital, civic engagement, empowerment, demographic structure and dynamics, …)<br>**supply of health care and social care organization matching with realities and needs** (development of	Write "**yes**" if force, "**no**" if challenge and "**na**" if not applicable.

Appendix 3. Coding grid

VARIABLE OF INTEREST (or component)	NAME OF VARIABLES	DESCRIPTION	MODES	COMMENTS (explanation or clarification)
			a continuum of care and services, organizational innovation and cultural change : patient education, care coordination, preventive care provided by a team,…)<br>**community-based care** (integration of health care and social care, coordination between systems that cover different geographical areas or different level on service intensity, …)<br>**sustainability** of health care and social care delivery<br>**place of health care and social care delivery** (accessible, convivial and safe environment for users, …)<br>**mobilization of resources** (financial, material and technical)<br>**renewal of healthcare et social care workers** (professionals and managers)<br>**interdisciplinary collaboration** (team working, coordination of interdisciplinary practices, mutual aid, …)<br>**cross-sectoral partnership** (involvement of several sectors, stable partnership, active community participation in deciding on care provision and resource allocation, patient engagement, cross-sectoral coordination, establishment of facilitating structures, …)<br>**personalised and flexible intervention** (people-centred care, outreach approach, ...)<br>**temporality** question (services delivery, patient involvement, ...)<br>**other** (specify)	
**Section Characteristics of the activities and strategy intervention** (HOW)				
**health_care_activities**	Health care activities of ICC	Describe the health activities or outputs associated to the ICC	**consultation**<br>**tracking/testing**<br>**medical and nursing care**	Write **nd** if not declared.

Appendix 3. Coding grid

**VARIABLE OF INTEREST** (or component**)**	**NAME OF VARIABLES**	**DESCRIPTION**	**MODES**	**COMMENTS** (explanation or clarification)
			**care promotion**<br>**preventive care**<br>**information and sensitization** (care professionals and target population)<br>**connection to intervention resources** (referrals to other specialty services intern and extern)<br>**self-care/empowerment support**<br>**other** (specify)	
**social_care_activities**	Social care activities of intervention approach	Describe the social activities or outputs associated to the ICC	**consultation**<br>**care promotion**<br>**prevention care**<br>**information and sensitization** (care professionals and target population)<br>**filling out forms** (financial support, …)<br>**connection to intervention resources** (referrals to other specialty services intern and extern)<br>**psychosocial support and coaching** (group or individual)<br>**support for collective mobilization** (social or recreational)<br>**pleading and advocacy**<br>**life competence support**<br>**rehabilitation assistance**<br>**self-care/empowerment support**<br>**other** (specify)	Write **nd** if not declared.
**management_care**	management care activities of intervention	Describe the management activities associated with the privileged approach (enabling structures).	**management of care delivery and environment** (group versus individual care, coordination of care amongst different provider, quality and safety systems, physical or sensory environment, outreach services, site of service delivery, size of organisations, …)<br>**management of care workers** (role expansion, self-management, length of consultation, staffing models, movement of health workers, between public and private care, recruitment and retention, …)<br>**management of care processes** (care pathways, case management, communication between provider	Source : Taxonomie EPOC 2015 : https://epoc.cochrane.org/sites/epoc.cochrane.org/files/public/uploads/taxonomy/epoc_taxonomy.pdf<br><br>Write nd if not declared.

Appendix 3. Coding grid

VARIABLE OF INTEREST (or component)	NAME OF VARIABLES	DESCRIPTION	MODES	COMMENTS (explanation or clarification)
			continuity of care, health and wellness management, integration, procurement and distribution, referrals system, shared care, shared decision-making, teams, transition of care, management with a people centred-care perspective, …)<br>**other** (specify)	
**governance_intervention_strategies**	governance of intervention strategy	Describe the preferred intervention strategy in terms of governance (implementation, partnership, ICTs, financing and accountability).	**strategies** targeted at **care organisations** (change of organisational culture)<br>**strategies** targeted at **care workers** (monitoring performance of care delivery, communities of practice, continuous quality, improvement, educational meetings, educational outreach, inter-professional education, local consensus processes, local opinion leaders, public release of performance data, tailored interventions, …)<br>**strategies** targeted at **health and social care governance and partnership** (care delivery, care environment, care workers, care processes, …)<br>**strategies** targeted at **cross-sectoral governance and partnership** (care delivery, care environment, care workers, care processes, …)<br>**information and communication technology** (health information systems, use of information and communication technology, telemedecine, …)<br>**financial arrangements and accountability** (financial resources for health and social care, payment of health and social care services, target financial incentives for health and social workers and organisations, health assurance, …)<br>**other** (specify)	**Source** : Taxonomie EPOC 2015 : https://epoc.cochrane.org/sites/epoc.cochrane.org/files/public/uploads/taxonomy/epoc_taxonomy.pdf<br><br>Write nd if not declared.

Appendix 3. Coding grid

PART 2 - DATA RELATED TO EMPIRICAL CONFIGURATIONS CONTEXT-MECHANISM-OUTCOME (CMOc) – FOR THE REALIST SYNTHESIS

CMO configurations statements outline the relationship between particular characteristics of contexts, mechanisms and effects in the following form: "In an 'X' context, the 'Y' mechanism generates the 'Z' effect".

As with the rough theories, the presentation of statements identified as potential empirical evidence distinguishes between CMO configurations that are intervention and management based. Furthermore, as they are extracted, the empirical data are analysed a posteriori to identify families of mechanisms in order to facilitate the identification of possible recurrences.

Empiricals specifics contexts (C)
The contextual elements identified and specific to a given intervention are directly extracted, if necessary the more general contextual elements (general context, challenges related to access, availability, continuity and quality of care, issues) and the elements related to the intervention (activities,intervention, management and governance strategies) previously recorded in the extraction grid are used to improve the analysis.
**Empiricals identified or hidden mecanisms (M)**
**Human reasoning of the actors** (ideas, choices and decisions) to how individual and collective change will be achieved and **reaction of the actors** (capacity to put their ideas, choices or decisions into practice) to the resources provided by the intervention produce effect in a given context.<br><br>Examples of potential mechanisms extracted from the raw theories that explain why and how the intervention works, from the practitioner's perspective:<br>- Openness and willingness to understand the area of intervention, the actors present and its population.<br>- Desire to share and exchange with other actors on the global vision of the characteristics and needs of the area and its population.<br>- Interest in deploying a proximity approach (spatial and relational proximity).<br>- Base its action on a proximity approach<br>- Support the individual empowerment of citizens<br>- Supporting the strengthening of citizens' collective power to act<br>- Intervene in proximity, in a flexible and adapted manner in synergy<br>- Openness to the involvement of citizens/users and their relatives in decisions concerning their care and services.<br>- valuing the contribution of the knowledge of all: practitioners, citizens/users and relatives<br>- Openness to partnership approaches<br>- Linking and dynamically adapting the practices and actions of the various actors involved in the same community<br>- other

Appendix 3. Coding grid

**Empiricals health and well-being outcomes (O)**
The effects of an intervention are generated by mechanisms linked to strategies deployed in a given context.<br>Examples of outcomes based on rough theories that explain the potential changes generated by the intervention mechanisms:<br>- holistic knowledge and understanding<br>- improve access to care services<br>- improve availability of care services<br>- improve continuity of care services<br>- improve quality and safety of care services<br>- multi-disciplinary team workers<br>- cross-sectoral partnership<br>- shared leadership and governance<br>- upgrade care services delivery (enabling structures)<br>- care practices approach (people centred care, gaining trust, outreach, …)<br>- information and communication<br>- other
