# Supplementary material for: Integrated Community Care Delivered by Public Health-Care and Social-Care Systems: Results from a Realist Synthesis
Source: Int J Integr Care. 2024 Feb 16;24(1):11. doi: 10.5334/ijic.7042 (PMC10870956; doi:10.5334/ijic.7042)
Supplement: Appendix 4. — List of included and excluded studies. [file ijic-24-1-7042-s4.pdf]

# Appendix 4. List of included and excluded studies

## 4.1 - List of eighteen scientific studies included

| First author (Paper publication) | Year | Title | Language of publication | Country of publication | Source | Approach | Targets all or a large part of the population of a given local area | Deployed by the public health and social care network, with or without collaboration |
|---|---|---|---|---|---|---|---|---|
| Shukor (The Permanente Journal) | 2018 | Developing Community-Based Primary Health Care for Complex and Vulnerable Populations in the Vancouver Coastal Health Region: HealthConnection Clinic | English | Canada | Databases | Integrated community care (health and social care, localized, cross-sectoral) | Yes | Yes |
| Hatano (International Journal of Integrated Care) | 2017 | The Vanguard of Community-based Integrated Care in Japan: The Effect of a Rural Town on National Policy | English | Japan | Databases | Integrated community care (health and social care, localized, cross-sectoral) | Yes | Yes |
| Eastwood (International Journal of Integrated Care) | 2019 | Designing an Integrated Care Initiative for Vulnerable Families: Operationalisation of Realist Causal and Programme Theory, Sydney Australia | English | Australia | Databases | Integrated community care (health and social care, localized, cross-sectoral) | Yes | Yes |
| Eastwood (International Journal of Integrated Care) | 2018 | The Healthy Homes and Neighbourhoods Integrated Care Initiative (conference resume, considred concurrently with 29) | English | Australia | Databases | Integrated community care (health and social care, localized, cross-sectoral) | Yes | Yes |
| Eastwood (International Journal of Integrated Care) | 2020 | Care Coordination for Vulnerable Families in the Sydney Local Health District: What Works for Whom, under What Circumstances, | English | Australia | Author follow-up | Integrated community care (health and social care, localized, cross-sectoral) | Yes | Yes |

| | | | | | | | | |
|---|---|---|---|---|---|---|---|---|
| | | and Why? | | | | | | |
| Tennant (BMC Health Services Research) | 2020 | A critical realist evaluation of an integrated care project for vulnerable families in Sydney, Australia | English | Australia | Databases | Integrated community care (health and social care, localized, cross-sectoral) | Yes | Yes |
| Eastwood (International Journal of Integrated Care) | 2020 | Refining Program Theory for a Place-Based Integrated Care Initiative in Sydney, Australia | English | Australia | Databases | Integrated community care (health and social care, localized, cross-sectoral) | Yes | Yes |
| Hamiti (Norois) | 2014 | Territorialisation des soins primaires : initiatives des collectivités et des professionnels de santé sur le Massif central pour une autre gouvernance | French | France | Databases | Localized and integrated cross-sectoral health care | Yes | Yes |
| Segura (Education for Health) | 2007 | Towards Unity for Health in the Barceloneta: An Innovative Experience in Community-Based Primary Health Care | English | Spain | Databases | Localized and integrated cross-sectoral health care | Yes | Yes |
| Fournier (Sciences Sociales et Santé) | 2014 | Concevoir une maison de santé pluri-professionnelle : paradoxes et enseignements d'une initiative en actes | French | France | Databases | Localized and integrated cross-sectoral health care | Yes | Yes |

| | | | | | | | | |
|---|---|---|---|---|---|---|---|---|
| Dalton (International journal of integrated care) | 2018 | Using the Project Integrate Framework for assessing progress towards care integration: results from a formative evaluation of a complex intervention in Central Coast Local Health D… (conference resume considered concurrently with S9-b) | English | Australia | Databases | Integrated community care (health and social care, localized, cross-sectoral) | Yes | Yes |
| Dalton (International Journal of Integrated Care) | 2019 | Formative Evaluation of the Central Coast Integrated Care Program (CCICP), NSW Australia | English | Australia | Author follow-up | Integrated community care (health and social care, localized, cross-sectoral) | Yes | Yes |
| Read (International Journal of Integrated Care) | 2019 | Using the Project INTEGRATE Framework in Practice in Central Coast, Australia | English | Australia | Author follow-up | Integrated community care (health and social care, localized, cross-sectoral) | Yes | Yes |
| Dalton (Evaluation report) | 2018 | Central Coast Integrated Care Program: Formative Evaluation Report for the Central Coast Local Health District (report considered concurrently with S9-b) | English | Australia | Author follow-up | Integrated community care (health and social care, localized, cross-sectoral) | Yes | Yes |
| Self (Canadian nurse) | 2005 | Street Outreach with No Streets | English | Canada | Databases | Localized and integrated cross-sectoral health care | Yes | Yes |
| Castriotta (BMJ Open) | 2020 | Measuring the impact of a social programme on healthcare: a 10-year retrospective cohort study in Trieste, Italy | English | Italy | Other | Integrated community care (health and social care, localized, cross-sectoral) | Yes | Yes |

| | | | | | | | | |
|---|---|---|---|---|---|---|---|---|
| Di Monaco (SSM Population Health) | 2020 | Promoting health equity through social capital in deprived communities: a natural policy experiment in Trieste, Italy | English | Italy | Other | Integrated community care (health and social care, localized, cross-sectoral) | Yes | Yes |
| Leask (AIMS Public Health) | 2019 | Implementation of a neighbourhood care model in a Scottish integrated context—views from patients | English | Scotland | Databases | Integrated community care (health and social care, localized, cross-sectoral) | Yes | Yes |

## 4.2 - List of selected grey publications (N=8)

| First author (paper publication) | Year | Title | Langage of publication | Country of publication | Source (Google scholar, Website, snowbal, other) | Approach | Targets all or a large part of the population of a given territory | Deployed by the public health and social care network with or without collaboration |
|---|---|---|---|---|---|---|---|---|
| Bufilco (Politecnico di Milano) | 2008 | The reflective Institution and the city : Space based policies upsetting organisations in TRIESTE | English | Italy | Other | Integrated community care (health and social care, localized, cross-sectoral) | Yes | Yes |
| De Titta (Collection « Recherches » du PUCA) | 2008 | TRIESTE : La sécurité comme effet collatéral dans la région Frioul Vénétie julienne | French or Italian | Italy | Other | Integrated community care (health and social care, localized, cross-sectoral) | Yes | Yes |
| Bricocoli (European Spatial Research and Policy) | 2012 | Growing old in cities. Council housing Estates in Trieste as laboratories for new perspectives in urban planning | English | Italy | Other | Integrated community care (health and social care, localized, cross-sectoral) | Yes | Yes |

| First author (paper publication) | Year | Title | Langage of publication | Country of publication | Source (Google scholar, Website, snowbal, other) | Approach | Targets all or a large part of the population of a given territory | Deployed by the public health and social care network with or without collaboration |
|---|---|---|---|---|---|---|---|---|
| Colle (Trieste Prima) | 2018 | La comunità che fa salute le microaree di Trieste per l'equità (treated concurently with 25) | Italian | Italy | Other | Integrated community care (health and social care, localized, cross-sectoral) | Yes | Yes |
| Morin (IUPLSSS du CIUSSS de l'Estrie - CHUS) | 2017 | Évaluation du déploiement des intervenants dédiés et des changements associés au sein de l'équipe quartier : Intervention de quartier (Sherbrooke) du CIUSSS de l'Estrie - CHUS | French | Canada | Website | Localized and integrated cross-sectoral social care | Yes | Yes |
| Morin (IUPLSSS du CIUSSS de l'Estrie - CHUS) | 2011 | L'Intervention de quartier au CSSS-IUGS une recherche évaluative | French | Canada | Website | Localized and integrated cross-sectoral social care | Yes | Yes |
| Morin (IUPLSSS du CIUSSS de l'Estrie - CHUS) | 2015 | L'intervention intersectorielle en santé et services sociaux dans ses liens avec les dimensions de l'habitation et le logement social | French | Canada | Website | Integrated community care (health and social care, localized, cross-sectoral) | Yes | Yes |
| Allaire (Private report) | 2021 | Évaluation Lac-Mégantic (CMO) | French | Canada | Author follow-up | Integrated community care (health and social care, localized, cross-sectoral) | Yes | Yes |

## 4.3 – List of excluded scientific studies (N=36)

| First author | Year | Title | Exclusion reason |
|---|---|---|---|
| **Robertson** | 2017 | Space, time and demographic change A geographical approach to integrating health and social care | Not an empirical study (theoretical study) |
| **Molina** | 2016 | From Design to Action in Clinical and Social Complexity: Continous Assesment of the Alt Penedés Program | Targets a population subgroup (patients with chronic diseases) |
| **Shaw** | 2012 | Extending primary care: potential learning from Italy | Targets a population subgroup (elderly patients with long-term illnesses) |
| **Pearson** | 2015 | Providing effective and preferred care closer to home: a realist review of intermediate care | Targets a population subgroup (long-term acute care patients) |
| **Morton-Chang** | 2017 | iCOACH: Integrated care as boundary spanning: Organizational workarounds in the delivery of community based primary health care | Initiative not deployed by the public health and social care network |
| **Meloche** | 2017 | INTEGRATE: How One Community is Advancing Integrated Care for Seniors | Targets a population subgroup (seniors suffering from chronic diseases) |
| **Leemrijse** | 2018 | Providing integrated health and social care to vulnerable populations in the community | Targets a population subgroup (patients with multiple health and psychosocial problems) |
| **Madsen** | 2018 | Implementation of innovative integrated models of Community-based Primary Healthcare CBPHC in Denmark iCoachDK - a study protocol | Not an empirical study (realist review) |
| **Phelan** | 2018 | Challenges in care co-ordination: missed care in community nursing | Issue of the specific local area of the intervention |
| **Tuner** | 2019 | Evaluating models of neighbourhood care | Targets a population subgroup (elderly people at the end of their lives and socially vulnerable) |
| **Pickett** | 2015 | American Journal of Psychiatric Rehabilitation | Targets a population subgroup (homeless people) and specific local area issues |
| **Eastwood** | 2019 | Sig health and social care working meeting | not an empirical study (reflection on the scope of the foundations of health and social care integration) |
| **Tumiel-Berhalter** | 2011 | The Implementation of Good for the Neighborhood: A Participatory Community Health Program Model in Four Minority Underserved Communities | Private initiatiave |
| **Cole** | 2008 | The Health Action Zone Initiative: Lessons from Plymouth | Multiple projects evaluation |
| **Arole** | 2009 | Sustainable transformation of communities: The Jamkhed experience- "We have done it ourselves!" [References] | NGO initiative. Accepted approach as an international standard. |

| First author | Year | Title | Exclusion reason |
|---|---|---|---|
| **DeHaven** | 2020 | Designing health care: A community health science solution for reducing health disparities by integrating social determinants and the effects of place | Targets a population subgroup and a specific research methodology (community-based participatory research (CBPR) practices for developing place-based initiatives) |
| **McCrory** | 2019 | An overview of the role of the district nurse caring for individuals with complex needs | Description of the practice of a single home care worker |
| **Henderson** | 2018 | Regional responses to the challenge of delivering integrated care to older people with mental health problems in rural Australia | Regional area level, remote from the local area |
| **Inoue** | 2019 | Implementation of a neighbourhood care model in a Scottish integrated context—views from patients | Analyses the issues that lead to having health zones closer to the populations to meet the needs. Also, strongly hospital-centred |
| **Stone** | 2019 | Investing in the Social Dimensions of Health: Community-based programs can bridge the medical and social worlds to meet older adults' needs | General discussion paper (not an empirical study) |
| **Aligué** | 2016 | Towards a Collaborative Integrated Healthcare Model in Tona | Conference abstract without follow-up information |
| **McLachlan** | 2016 | Waluwin - an integrated approach towards health and wellbeing in Western NSW, Australia | Conference abstract without follow-up information |
| **McLachlan** | 2018 | It Pays to be Well Connected - Delivering an integrated system of care in Western New South Wales, Australia | Conference abstract without follow-up information |
| **Becker** | 2005 | An innovative geographical approach: health promotion and empowerment in a context of extreme urban poverty | Initiative by an NGO |
| **Schulte** | 2017 | Conducting a Health Needs Assessment to Identify Potentials for Population-based Integrated Care Models in Socially Deprived Urban Regions – The Example of the Integrated Care Project "Billstedt-Horn" in Hamburg, Germany | Conference abstract without follow-up information |
| **McLachlan** | 2017 | Delivering an integrated system of care in Western New South Wales, Australia | Conference abstract without follow-up information |
| **Stairmand** | 2018 | Prototyping Innovation in Place-Based Integrated Care Teams in Eastern Cheshire | Conference abstract without follow-up information |
| **Allworth** | 2018 | An exploration of models of care coordination to meet the needs of families requiring health and social care in Sydney, Australia' | Conference abstract without follow-up information |

| First author | Year | Title | Exclusion reason |
|---|---|---|---|
| **van de Schoot** | 2018 | Building Blocks of Knowledge on integrated care for professionals in neigborhood teams | Conference abstract without follow-up information |
| **Charles** | 2019 | Designing integrated primary care in Toronto Canada | Conference abstract without follow-up information |
| **D'Ambrosio** | 2017 | Coordinated care for older adults utilizing the flourish model - A shared care approach | Conference abstract without follow-up information |
| **Gray** | 2018 | Mapping for Conceptual Clarity: Exploring, Implementation of Integrated Community-Based Primary - Health Care from a Whole Systems Perspective | Targets a population subgroup (elderly with complex needs) |
| **Anderson** | 2012 | Community Health Centers and the Patient-Centered Medical Home: Challenges and Opportunities to Reduce Health Care Disparities in America | Issue of the specific territory of the intervention |
| **Morikawa** | 2014 | Towards community-based integrated care: trends and issues in Japan's long-term care policy | Issue of the specific local area of the intervention and no empirical results |
| **Robelet** | 2005 | La coordination dans les réseaux de santé : entre logiques gestionnaires et dynamiques professionnelles | Issue of the specific local area of the intervention |
| **Eastwood** | 2019 | Implementation, Mechanisms of Effect and Context of an Integrated Care Intervention for Vulnerable Families in Central Sydney Australia: A Research and Evaluation Protocol | Not an empirical study |

## 4.4 – List of grey literature excluded (N=9)

| First author | Year | Title | Exclusion reason |
|---|---|---|---|
| Le collège des médecins de familes du Canada (CMFC) | 2018 | Innovation in Primary Care: Caring for Unattached and Marginalized Patients | Target population subgroups |
| Hass | 2013 | Les collectivités territoriales et l'offre de soins de proximité | Issue of the specific local area of the intervention |
| Healthcare Improvement Scotland | 2019 | Learning from neighbourhood care test sites in Scotland | Issue of the specific local area of the intervention |
| Morin | 2019 | Cadre de référence à l'intention des établissements : Améliorer l'accès, la qualité et la continuité des services de proximité généraux et de santé mentale | Not an empirical study |
| Morin | 2015 | Intervention de proximité en CSSS, une pratique de pointe du CSSS-IUGS : guide d'accompagnement | Not an empirical study |

| First author | Year | Title | Exclusion reason |
|---|---|---|---|
| NSW Ministry of Health (Australie) | 2018 | NSW Health: Strategic Framework for Integrating Care | Issue of the specific local area of the intervention |
| TransForm Integrated Community Care (Transnational Forum on ICC) | 2018 | 1st Transnational Conference on Integrated Community Care: 'Making the case for Integrated Community Care' (Hamburg, Germany) | Summary report of the conference papers, discussions and lessons learned |
| TransForm Integrated Community Care (Transnational Forum on ICC) | 2019 | 2nd Transnational Conference on Integrated Community Care: “Towards People-Driven Care” Engaging and Empowering Individuals, Carers and Families through Integrated Community Care (Turin, Italy) | Preparatory document and summary report of the conference papers, discussions and lessons learned |
| TransForm Integrated Community Care (Transnational Forum on ICC) | 2019 | 3rd Transnational Conference on Integrated Community Care: ‘Building stronger communities through Integrated Community Care’ (Vancouver – Canada) | Preparatory document and summary report of the conference |
