# Supplementary material for: Integrated Community Care Delivered by Public Health-Care and Social-Care Systems: Results from a Realist Synthesis
Source: Int J Integr Care. 2024 Feb 16;24(1):11. doi: 10.5334/ijic.7042 (PMC10870956; doi:10.5334/ijic.7042)
Supplement: Appendix 5. — MMAT. [file ijic-24-1-7042-s5.pdf]

Appendix 5. Assessment of study methodological quality (MMAT)

				SCREENING QUESTIONS		1. QUALITATIVE STUDIES					Overall assessment of methodological quality or rigor (poor/adequate/average/good/excellent and comment)	Type of approach (territorialized community health care, territorialized community social services, or territorialized and integrated community health and social services)
RefID	First author	Year	Citation	S1. Are there clear research questions?	S2. Do the collected data allow to address the research questions?	1.1. Is the qualitative approach appropriate to answer the research question?	1.2. Are the qualitative data collection methods adequate to address the research question?	1.3. Are the findings adequately derived from the data?	1.4. Is the interpretation of results sufficiently substantiated by data?	1.5. Is there coherence between qualitative data sources, collection, analysis and interpretation?		
						5. MIXED METHODS STUDIES						
						5.1. Is there an adequate rationale for using a mixed methods design to address the research question?	5.2. Are the different components of the study effectively integrated to answer the research question?	5.3. Are the outputs of the integration of qualitative and quantitative components adequately interpreted?	5.4. Are divergences and inconsistencies between quantitative and qualitative results adequately addressed?	5.5. Do the different components of the study adhere to the quality criteria of each tradition of the methods involved?		
S1	**2. Shukor** (The Permanente Journal)	2018	Developing Community-Based Primary Health Care for Complex and Vulnerable Populations in the Vancouver Coastal Health Region: HealthConnection Clinic	Yes<br>This case study contributes to provide a contextual narrative of the development of HealthConnection Clinic and a systematic descriptive evaluation of its structures and processes using the Building Blocks as a framework.	Yes<br>**Primary and secondary data**	Yes<br>**Appropriate link between the mixed methods approach used and the research question**	Yes<br>**Data collection techniques/methods and appropriate data sources**<br>* Qualitative data collected through participatory observation, document analysis, emails, and a series of iterative meetings and analytical processes with clinic team members.<br>* Quantitative data derived from the primary care EMR, the Patient Automated Records Information Systems, and hospital administrative databases were derived using VCH's client experience survey tool.	Yes	Yes<br>**Interpretation of results**<br>Supported by the data collected	Yes<br>**Clear links**	Excellent<br>**High quality study**	Localized and integrated health care and social services
S3	**3. Hatano** : (International Journal of Integrated Care)	2017	The Vanguard of Community-based Integrated Care in Japan: The Effect of a Rural Town on National Policy	Yes<br>What are the challenges of implementing a community-based integrated health and social services system and introducing the community-based approach of an integrated community-based care system into national policy to address the consequences of an aging population?	Yes<br>**Primary and secondary data**	Yes<br>**Appropriate link between the mixed methods approach used and the research question**<br>Empirical study based on the exploration of a model of integrated community-based health and social services experienced in rural areas	Yes<br>**Techniques/methods of data collection and suitable sources.**<br>* Baseline survey of elderly people living at home, Hiroshima Prefecture. 1975-1990 * Health and welfare activities in Mitsugi City, Mitsugi General Hospital,1980-2004 * Various archival and reference materials (Integrated community care system in Mitsugi City, Onomichi City, ... Mitsugi General Hospital, 2012, ...)	Do not know<br>**Data analysis methods**	Do not know<br>**Interpretation of results**	Do not know<br>**if clear links**	Appropriate<br>**Average quality study**	Localized and integrated health care and social services
S5-a	**4. Eastwood** (International Journal of Integrated Care)	2019	Designing an Integrated Care Initiative for Vulnerable Families: Operationalisation of Realist Causal and Programme Theory, Sydney Australia	Yes<br>Describe the development of an interagency care coordination network design for vulnerable families, inspired by earlier work on causal realist and program theory	Yes<br>**Primary and secondary data**	Yes<br>**Appropriate link between mixed methods approach used and research question** Evidence-based theory associated with an interagency integrated health and social care coordination initiative	Yes<br>**Appropriate data collection techniques/methods and data sources**<br>* Realistic, critical, longitudinal, and multi-level design<br>* Evaluation of applied program interventions	Yes<br>**Data Analysis Methods**<br>4 phases of analysis: 1) operationalization of program theory and intervention development and planning; 2) intervention evaluation; 3) theory testing studies; 4) dissemination of results	Yes<br>**Interpretation of results**<br>Supported by the data collected	Yes<br>**Clear links**	Excellent<br>**High quality study**	Localized and integrated health care and social services

Appendix 5. Assessment of study methodological quality (MMAT)

S5-b	**4.22. Eastwood** (International journal of integrated care)	2019	Implementation, Mechanisms of Effect and Context of an Integrated Care Intervention for Vulnerable Families in Central Sydney Australia: A Research and Evaluation Protocol	Yes<br>Process evaluation of the operationalization, contextualization, implementation, and effects of a local integrated care initiative for healthy homes and neighborhoods	Yes<br>**Primary data**	Yes<br>**Appropriate linkage between mixed methods approach used and research question**<br>Formative and summative evaluation	Yes<br>**Techniques/methods of data collection and appropriate data sources** Combination of several methodological approaches: critical realism, evaluation of complex interventions, theory of change, logic models, scientific approaches to continuous improvement, mixed methods research	Yes<br>**Clear data analysis methods**<br>* Iterative approach to continuous design, testing and evaluation<br>* Realistic mixed-method studies and quantitative modeling (evaluation)	No<br>**Interpretation of results**<br>Not applicable to the research and evaluation protocol	No<br>Clear links<br>**Does not apply to the research and evaluation protocol**	**Conference abstrat - no evaluation**	Localized and integrated health care and social services
S5-c	**30. Eastwood** (International Journal of Integrated Care)	2018	The Healthy Homes and Neighbourhoods Integrated Care Initiative (conference summary)	Yes<br>How to break the intergenerational cycle of disadvantage for vulnerable families through the co-design, by diverse cross-sector partners, of services and the implementation of an evidence-informed integrated care initiative	Does not apply	Does not apply	Does not apply	Does not apply	Does not apply	Does not apply	Does not apply	Localized and integrated health care and social services
S5-d	**Eastwood** (International Journal of Integrated Care)	2020	Care Coordination for Vulnerable Families in the Sydney Local Health District: What Works for Whom, under What Circumstances, and Why?	Yes<br>For whom, when and why the care coordination component of HHAN works, and establish the reported outcomes for clients, service-providers and partner organisations	Yes<br>**Primary and secondary data**	Yes<br>**Appropriate linkage between qualitative approach used and research question**<br>A thematic analysis was undertaken to identify key modes of HHAN intervention, and data representing these elements were coded and summarized under the headings of contexts, mechanisms, and outcomes.	Yes<br>**Appropriate data collection techniques/methods and data sources**<br>A qualitative evaluation of the impact of care coordination. Purposive sampling was used to select a total of 37 participants for interview, including consumers, service-providers and key stakeholders.	Yes<br>**Clear data analysis methods**	Yes<br>**Interpretation of results**<br>Supported by the data collected	Yes<br>**Clear links**	**Excellent**<br>**High quality study**	Localized and integrated health care and social services
S5-e	**Tennant_Eastwood** (BMC Health Services Research**)**	2020	A critical realist evaluation of an integrated care project for vulnerable families in Sydney, Australia	Yes<br>Longitudinal development and evaluation of complex integrated health and social care interventions in Sydney, Australia.	Yes<br>**Primary data**	Yes<br>**Appropriate link between the qualitative approach used and the research question**<br>describe the qualitative component of a critical realist pilot case study aimed at exploring, explaining and refining emerging HHAN programme theories in relation to care coordination.	Yes<br>**Appropriate data collection techniques/methods and data sources**<br>Qualitative interviews were undertaken with HHAN clients (n=12), staff and other stakeholders (n=21). Interviews and coding used a context (C), mechanism (M) and outcome (O) framework.	Yes<br>**Clear Data Analysis Methods**<br>Inductive, deductive, retroductive, and abductive modes of reasoning were used with the CMO heuristic tool to inform the developing program theory.	Yes<br>**Interpretation of results**<br>Supported by the data collected	Yes<br>**Clear links**	**Excellent**<br>**High quality study**	Localized and integrated health care and social services

Appendix 5. Assessment of study methodological quality (MMAT)

S5-f	**Eastwood** (International Journal of Integrated Care)	2020	Refining Program Theory for a Place-Based Integrated Care Initiative in Sydney, Australia	Yes<br>Hence, this study aims to evaluate for whom, when and why HHAN's place-based initiatives (PBI) at Redfern works, or not, and what are its outcomes.	Yes<br>**Primary data**	Yes<br>**Appropriate link between qualitative approach used and research question**<br>The project uses a critical realist methodology to undertake a qualitative assessment of the impact of the BIP on clients, services, and the community.	Yes<br>**Appropriate data collection techniques/methods and data sources**<br>Purposive sampling was used to identify 21 participants including HHAN clients, HHAN staff and stakeholders (HHAN partners). In-depth, semi-structured interviews were audio-recorded, transcribed, coded and analysed.	Yes<br>**Clear data analysis methods**<br>In-depth, semi- structured interviews were audio-recorded, transcribed, coded and analysed using a context (C), intervention (I), mechanism (M) outcome (O) (CIMO) approach to abductive analysis.	Yes<br>**Interpretation of results**<br>Supported by the data collected	Yes<br>**Clear links**	Excellent<br>**High quality study**	Localized and integrated health care and social services
S6	**25. Hamiti** (Presses universitaires de Rennes)	2014	Territorialisation des soins primaires : initiatives des collectivités et des professionnels de santé sur le Massif central (plusieurs villes de la France, incluant Clermont-Ferrand, Limonges et Saint-Étienne) pour une autre gouvernance [Territorialization of primary care: initiatives of communities and health professionals in the Massif Central (several cities in France, including Clermont-Ferrand, Limonges and Saint-Étienne) for a different governance]	Yes<br>Describe the contexts in which community initiatives emerge, their motivations, their actions, their difficulties and the impact of their involvement on local health professionals	Yes<br>**Primary and secondary data**	Yes<br>**Appropriate link between qualitative approach used and research question**<br>Empirical study based on the experience of a territorialized primary health care initiative, tested in fragile rural territories (socio-economic dynamics)	Yes<br>**Techniques/methods of data collection and appropriate data sources**<br>* Individual/collective interviews recorded and transcribed, note-taking from project meetings, participant observation<br>* Participants: testimonies of elected officials, community technicians, health professionals	Yes<br>**Clear data analysis methods**<br>Comparative analysis, analysis of recorded conversations	Yes<br>**Interpretation of results**<br>Supported by the data collected	Yes<br>**Clear links**	Excellent<br>**High quality study**	Localized primary health care
S7	**27. Segura** (Education for Health)	2007	Towards Unity for Health in the Barceloneta: An Innovative Experience in Community-Based Primary Health Care	Yes<br>Describe the innovative aspects of the project, the evidence of its impact on the population, analyze its successes and some of the reasons for its eventual termination in light of the principles of the health union	yes<br>**Primary and secondary data**	yes<br>**Appropriate link between the qualitative approach used and the research question**<br>Qualitative description of a professional coordination model of community-based primary health care, involving the active participation of community members (individuals and groups)	don't know<br>**Data collection techniques/methods and data sources**	don't know<br>**Data analysis methods**	yes<br>**Interpretation of results**<br>Supported by the data collected	do not know<br>**if clear links**	Weak<br>**Study of doubtful quality**	Localized community health care
S8	**29. Fournier** (Sciences Sociales et Santé)	2014	Concevoir une maison de santé pluri-professionnelle : paradoxes et enseignements d'une initiatives en actes [Designing a multi-professional health centre: paradoxes and lessons learned from an initiative in action]	Yes<br>Data on their motivations, their actions, their difficulties and the impact of their involvement on local health professionals	Yes<br>**Primary and secondary data**	Yes<br>**Appropriate link between the mixed methods approach used and the research question**<br>case study of an innovation project bringing together primary care professionals in a multi-professional health centre, with a view to encouraging the emergence of new social practices	Yes<br>**Data collection techniques/methods and appropriate data sources**<br>* Participatory observation from March 2011 to June 2013 - Repeated in-depth interviews with the project's initiating team.<br>* Notes taken during informal discussions, email threads and documents produced by professionals around the project	Yes<br>**Clear data analysis methods**<br>Material analyzed according to a comprehensive and inductive sociology approach, coded according to 3 dimensions: by open categories, then transversally by types of personnel, then selectively according to the sociology of translation analysis framework	Yes<br>**Interpretation of results Supported by the data collected**<br>Theoretical coding, conversation analysis, document analysis, process description supported by data (verbatims)	Yes<br>**Clear links**	Excellent<br>**High quality study**	Localized community health care

Appendix 5. Assessment of study methodological quality (MMAT)

S9-a	**32. Dalton** (International journal of integrated care)	2018	Using the Project Integrate Framework for assessing progress towards care integration: results from a formative evaluation of a complex intervention in Central Coast Local Health D… (conference summary)	Does not apply	Does not apply	Does not apply	Does not apply	Does not apply	Does not apply	Does not apply	Does not apply	Localized primary health care
S9-b	**32.1. Dalton** (International Journal of Integrated Care)	2019	INTEGRATED CARE CASE : **Formative Evaluation** of the Central Coast Integrated Care Program (CCICP), NSW Australia	Yes<br>Evaluation of progress toward integration of health and social services to disseminate and strengthen the sustainability of the integrated care project	Yes<br>**Primary data**	Yes<br>**Appropriate link between the mixed methods approach used and the research question**<br>Formative evaluation	Yes<br>**Data collection techniques/methods and appropriate data sources**<br>* Online survey, assessment workshop, interviews<br>* Project integration framework used to assess progress toward integration of care	Yes<br>**Clear data analysis method**<br>comparative analysis	Yes<br>**Interpretation of results**<br>Supported by the data collected	Yes<br>**Clear links**	Excellent<br>**High quality study**	Localized primary health care
S9-c	**32.2. Read_Dalton** (International Journal of Integrated Care)	2019	RESEARCH AND THEORY : Using the **Project INTEGRATE Framework in Practice in Central Coast,** Australia	Yes<br>reports the first formative evaluation to provide a detailed description of the implementation of the CCICP, after two years of activity, and the current progress towards integrated care.	Yes<br>**Primary data**	Yes<br>**Appropriate link between the mixed methods approach used and the research question**<br>Formative evaluation: mixed methods approach	Yes<br>**Appropriate data collection techniques/methods and data sources**<br>* Progress towards integrated care achieved by the CCICP was evaluated using the Project INTEGRATE Framework data in a mixed methods approach included semi-structured interviews (n = 23) and Project INTEGRATE Framework based surveys (n = 27).<br>* All data collected involved key stakeholders, with close involvement in the program, self-reporting.	Yes<br>**Clear data analysis method**	Yes<br>**Interpretation of results**<br>Supported by the data collected	Yes<br>Clear links	Excellent<br>**High quality study**	Localized primary health care
S9-d	**32.3. Dalton** (evaluation report)	2018	Central Coast Integrated Care Program : Formative Evaluation Report for the Central Coast Local Health District	Yes<br>Assessing the progress of the local movement toward patient- and population-centered care	Yes<br>**Primary data**	Yes<br>**Appropriate link between the mixed methods approach used and the research question**<br>Formative evaluation	yes<br>**Appropriate data collection techniques/methods and data sources**<br>* The evaluation method included the analysis of a variety of governance, administrative, project documents and "in house" analyses from population health and others.<br>* A framework derived from a multi-country European study named Project Integrate was used as a survey instrument, the basis of key informant interviews and a series of review meetings by the project team.	Yes<br>**Clear data analysis method**<br>Two international experts advised the evaluation and the project team throughout	Yes<br>**Interpretation of results**<br>Supported by the data collected	Yes<br>**Clear links**	Excellent<br>**High quality study**	Localized primary health care
S10	**35. Self** (Canadian nurse)	2005	Street Outreach with No Streets	Yes<br>In what contexts, how, for whom and to what effect does the outreach and street nursing program work in rural areas?	Yes<br>**Primary data**	Yes<br>**Appropriate link between qualitative approach used and research question**<br>Empirical study based on the experience of a successful street outreach and nursing program deployed in small rural communities to address the needs of people living on the street or marginalized	Do not know<br>**Data collection techniques/methods and data sources**	Do not know<br>**Data analysis methods**	Yes<br>**Interpretation of results**<br>Supported by the data collected	Do not know if clear links	**Weak**<br>**Study of doubtful quality**	Localized community health care

Appendix 5. Assessment of study methodological quality (MMAT)

S12	**Castriotta** (BMJ Open)	2020	Measuring the impact of a social programme on healthcare: a 10-year retrospective cohort study in Trieste, Italy	yes<br>measure the impact of a social programme on healthcare: a 10- year retrospective cohort study in Trieste, Italy	yes<br>**Primary and secondary data**	Yes<br>**Appropriate linkage between mixed methods approach used and research question**<br>A retrospective cohort study to evaluate the effectiveness of this community-based social intervention in terms of reducing urgent hospital and emergency room admissions.	Yes<br>**Data collection techniques/methods and data sources** Datas : linkage with the administrative databases of the Regional Repository of MicroData of the Friuli Venezia Giulia region, using an anonymous identifier.	Yes<br>**Clear data analysis method**<br>Design Retrospective cohort study	Yes<br>**Interpretation of results**<br>Supported by the data collected	Yes<br>**Clear links**	Excellent<br>**High quality study**	Community-based intervention
S13	**Di Monaco** (Elsevier Ltd.)	2020	Promoting health equity through social capital in deprived communities: a natural policy experiment in Trieste, Italy	Yes<br>Empirically evaluate of the social mechanisms through which social capital may have an impact on health inequalities (evaluate the presence of causal links between social capital and health indicators in two converging ways)	yes<br>**Primary and secondary data**	Yes<br>**Appropriate link between the mixed methods approach used and the research question**<br>The empirically evaluation was carried out during 2016–2018 and used both qualitative and quantitative methods. The qualitative part investigated the field experience of the HMP through interviews, focus groups and workshops with HMP professionals. The quantitative part assessed the effect HMP might have on these properties and the capacity to face health risks of more vulnerable individuals.	Yes<br>**Appropriate data collection techniques/methods and data sources**<br>Three samples, each of 200 individuals, residing in the target and in control areas were interviewed using a semi-structured questionnaire. One control sample was matched to the 200 treated subjects using a Propensity Score Matching.	Yes<br>**Clear data analysis method**<br>Research strategy: a mix of qualitative and quantitative methods focused to causal links to gain a deeper insight :<br>* A chronological and logical analysis (qualitative)<br>* A descriptive analyses compared (quantitative)	Yes<br>**Interpretation of results**<br>Supported by the data collected	Yes<br>**Clear links**	Excellent<br>**High quality study**	Health and social capital
S14	Least (AIMS Public Health	2019	Implementation of a neighbourhood care model in a Scottish<br>integrated context—views from patients	Yes<br>The aim of this paper is to explore the acceptability of a new neighbourhood care model by understanding the experiences of patients being supported by a self-managing, community-based, integrated, health and social care team.	Yes<br>**Primary and secondary data**	Yes<br>**Appropriate link between the qualitative methods approach used and the research question**<br>evaluation of the acceptability of a new model of community-based, integrated, health and social care to patients by understanding their experience (thematic analysis : 1) Service Operation; 2) Staff Qualities; 3) Acceptability & Assets; 4) Confounding Factors.	Yes<br>**Appropriate data collection techniques/methods and data sources**<br>In total, 43 patients were supported over the first four months of implementation, with individual interviews conducted with eight patients from across both sites. Demographic information collated included: age; sex; primary referral reason and referral source. Satisfaction questionnaires were also administered, assessing components. Interviews were based on a semi-structured topic guide. Discussions were based on a series of exploratory questions regarding patients' experience of being supported by the INCA teams. Fieldnotes were also taken during discussions and used as a reference point during analysis.	Yes<br>**Clear data analysis method**<br>Audio recordings were transcribed verbatim and analysed thematically using NVIVO Version 11 (QSR International, Doncaster). Thematic analysis is useful towards understanding patterns occurring in the data in order to improve understanding on a particular topic [10], such as the experience of being cared for by a self-managing, integrated, health and social care team. Analysis followed the six step framework previously described by Braun and Clark [11¨. The data were analysed independently by two researchers and then findings compared and adapted if required	Yes<br>**Interpretation of results**<br>Supported by the data collected	Yes<br>**Clear links**	Excellent<br>**High quality study**	Community-based, integrated, health and social
