# Supplementary figures and images for: Integrated Community Care Delivered by Public Health-Care and Social-Care Systems: Results from a Realist Synthesis
Source: Int J Integr Care. 2024 Feb 16;24(1):11. doi: 10.5334/ijic.7042 (PMC10870956; doi:10.5334/ijic.7042)

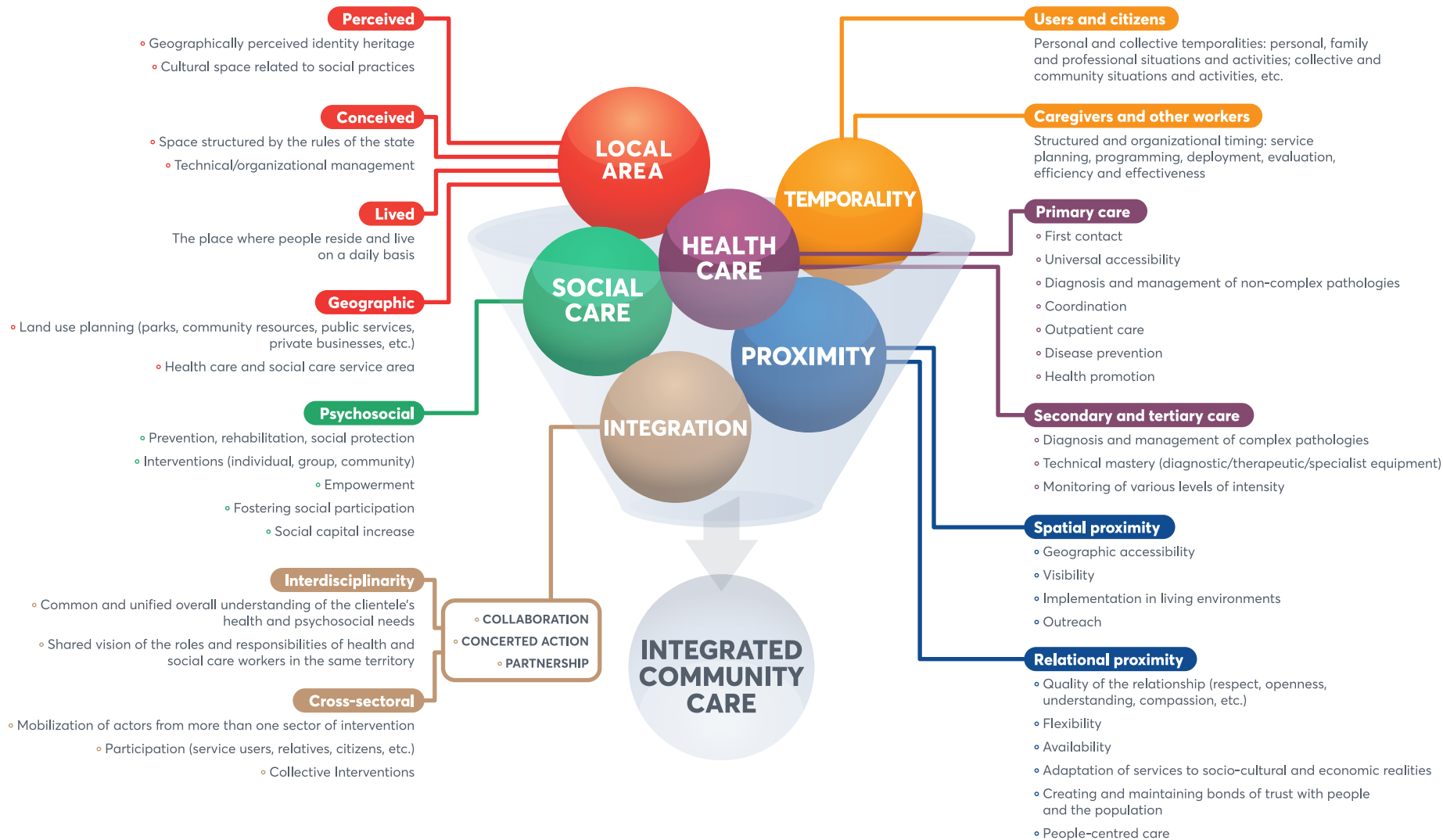

Supplement: Appendix 6. — First ICC model. [file ijic-24-1-7042-s6.pdf]
